# The PROTAC selectively degrading Bcl-x_L_ represents a novel Hedgehog pathway inhibitor with capacity of combating resistance to Smoothened inhibitors while sparing bone growth

**DOI:** 10.7150/thno.75421

**Published:** 2022-10-24

**Authors:** Shaoqing Zhang, Yue Chen, Zhongli Xu, Jun Yang, Renhong Sun, Juan Wang, Yiming Sun, Biao Jiang, Xiaobao Yang, Wenfu Tan

**Affiliations:** 1 Department of Pharmacology, School of Pharmacy, Fudan University, Shanghai 201203, P.R. China.; 2 Shanghai Institute for Advanced Immunochemical Studies, ShanghaiTech University, Shanghai 201210, P.R. China.; 3 Gluetacs Therapeutics (Shanghai) Co., Ltd., No. 99 Haike Road, Zhangjiang Hi-Tech Park, Shanghai, 201210, P. R. China.; 4 Division of Antitumor Pharmacology, State Key Laboratory of Drug Research, Shanghai Institute of Materia Medica, Chinese Academy of Sciences, 555 Zuchongzhi Road, Shanghai, 201203, P. R. China.

**Keywords:** Hedgehog, Bcl-x_L_, PROTAC, drug resistance, bone growth

## Abstract

**Rationale:** Primary and acquired resistance to Smoothened (Smo) inhibitors largely hampered their clinical efficacy. Given the important functions of hedgehog (Hh) pathway in bone formation and development, the permanent defects in bone growth caused by Smo inhibitors further restrict the use of Smo inhibitors for pediatric tumor patients. Anti-apoptotic Bcl-2 proteins regulate Hh activity by engaging a Bcl-2 homology (BH) domain sequence found in suppressor of fused (Sufu). In this study, we tested the effect of SIAIS361034, a Proteolysis Targeting Chimera (PROTAC) specifically targeting B-cell lymphoma extra large (Bcl-x_L_) to the celeblon (CRBN) E3 ligase for degradation, on combating the resistance and reducing the toxicity of bone growth caused by Hh inhibition.

**Methods:** Fluorescence polarization, homogeneous time-resolved fluorescence (HTRF) assay, immunoblot, and immunoprecipitation (IP) were used to evaluate whether SIAIS361034 is an appropriate Bcl-x_L_ PROTAC. Dual luciferase reporter assay, real-time quantitative PCR (RT-qPCR), depilatory model, and SmoA1 model were established to assess the effect of SIAIS361034 on the activity of Hh signaling pathway and its ability to overcome drug resistance* in vitro* and *in vivo*. Molecular mechanisms of SIAIS361034 for inhibiting Hh activity were demonstrated by dual luciferase reporter assay, immunoblot, and immunofluorescence staining. PET-CT and histopathology of bone tissues were used to assess the effects of SIAIS361034 on bone growth.

**Results:** We observed that SIAIS361034 efficiently and selectively inhibits the activity of the Hh pathway *in vitro* and *in vivo*, by interrupting Bcl-x_L_/Sufu interaction, therefore, promoting the interaction of Sufu with Gli1. Moreover, SIAIS361034 possesses the ability of combating resistance to current Smo inhibitors caused by *Smo* mutations and *Gli2* amplification and remarkably inhibits the growth of SmoA1 tumors *in vivo*. In contrast to von Hippel-Lindau (VHL) E3 ligase, our result further reveals little detectable expression of CRBN in two types of cells critical for bone development, human articular chondrocytes and human fetal osteoblastic cells. Moreover, treatment with SIAIS361034 results in no impairment on the bone growth of young mice, accompanying no alteration of the expression of Bcl-x_L_ and Gli1 proteins.

**Conclusion:** Our findings demonstrate that selectively targeting Bcl-x_L_ by PROTAC is a promising strategy for combating resistance to Smo inhibitors without causing on-target drug toxicities of bone growth.

## Introduction

Hedgehog (Hh) signaling plays a pivotal role in a variety of developmental events in mammals, such as embryogenesis and postnatal tissue homeostasis 1-3. In vertebrate organisms, three Hh ligands (Shh, Dhh, and Ihh) have been identified to bind to the same receptor Patched 1 (Ptch1), a multi-pass cell surface receptor. Without Hh ligands stimulation, Ptch1 suppresses the activity of seven-transmembrane G protein-coupled receptor, Smoothened (Smo). Upon ligands binding, however, this inhibition is relieved, allowing Smo to accumulate in the primary cilium and positively regulating the mobilization of zinc-finger transcription factors Gli proteins to the nucleus. Ultimately, the activation of transcriptional factors Gli induces the expression of Hh target genes, which are involved in proliferation and apoptosis, such as *Gli1*, *Ptch1*, and *Bcl2* 3, 4.

Inappropriate activation of Hh signaling has been shown to be involved in several types of tumors formation, including basal cell carcinoma (BCC) and medulloblastoma (MB) 5-7. The mechanisms associated with constitutive activation of Hh pathway for cancer development can be classified into ligand-independent and ligand-dependent manners. Ligand-independent activation of Hh pathway is characterized by sporadic somatic mutations in *Ptch1*, *Smo*, and *Suppressor of fused* (*Sufu*) or genomic amplification of *Gli2*, which were found in the overwhelming majority of Hh-driven BCC and MB 8-11. In addition, the irregular elevation of Hh ligands from tumors or stromal cells, namely ligand-dependent manner, has been documented in a wide range of cancers 12, 13. Larger scale efforts have been devoted in recent years to developing Hh inhibitors by targeting Smo for the treatment of tumors addiction to Hh pathway. GDC-0449, LDE-225, and PF-04449913 have been approved successively by the FDA for the treatment of locally advanced and metastatic BCC and acute myeloid leukemia (AML), respectively 14-18. However, the high incidence of tumor recurrence with the clinical application of GDC-0449 and LDE-225 limits their enduring efficacy 19, 20. Resistance to current Smo inhibitors can be acquired by *Smo* mutations as well as alterations in critical molecules downstream of Smo, including *Sufu* deletions and *Gli2* amplification 21-23. Moreover, due to the importance of Hh signaling pathway in bone development, Hh inhibitors suppress proliferation and differentiation of chondrocytes and lead to a dramatic expansion of the hypertrophic zone and premature bone growth plate closure 24-26. The aberrant bone structures formed appear to be irreversible and may not be removed by bone remodeling 27. Following treatment with GDC-0449 and LDE-225, pediatric patients whose bones have not achieved full maturity were found to develop premature widespread growth plate fusions 28-30. Therefore, alternative strategies are highly needed to overcome resistance and reduce bone growth toxicity of current Hh inhibitors.

Wu et al. recently reported that anti-apoptotic Bcl-2 families engage a Bcl-2 homology (BH) domain sequence discovered in Sufu, a critical negative regulator of Hh activity. This engagement promotes Sufu turnover and liberates Gli by interrupting Sufu-Gli interaction, thereby activating the Hh pathway and inducing the expression of Hh target genes 31. Hence, targeting anti-apoptotic Bcl-2 families may be a promising strategy for inhibiting Hh pathway and countering Smo inhibitors resistance. Proteolysis Targeting Chimera (PROTAC) is an emerging protein degradation technology that degrades protein of interest through the ubiquitin proteasomes system (UPS) by recruiting the target protein to E3 ligase for ubiquitination and eventually degradation by 26S proteasome 32, 33. Moreover, it has been shown that PROTAC is a good strategy for combating on-target toxicity, such as thrombocytopenia caused by Bcl-x_L_ inhibition, by taking advantage of the differential context expression of E3 ligase, von Hippel-Lindau (VHL) and celeblon (CRBN) 34-36. Therefore, it conceivably prompted us to develop PROTACs targeting Bcl-2 family proteins to inhibit Hh activity while sparing its target toxicity, bone growth.

Here we reported a selective Bcl-x_L_ PROTAC degrader, termed SIAIS361034, targeting Bcl-x_L_ to the CRBN E3 ligase for degradation, which is minimally expressed in bone tissues. We have shown that SIAIS361034 selectively degrades Bcl-x_L_ and significantly inhibits the Hh pathway activity with the capacity of overcoming the resistance to Smo inhibitors caused by mutations in *Smo* and amplification of *Gli2*. In addition, we provided evidence that SIAIS361034 exhibits no influence on bone growth. These findings reveal a novel strategy to combat resistance and reduce the toxicities of bone growth associated with current Smo inhibitors.

## Materials and Methods

### Chemical synthesis

The chemical structures and synthetic schemes for SIAIS361034 and SIAIS361034NC are presented in Figure S1C. Detailed synthetic procedures are provided in Supplementary data 1.

### Cell lines and reagents

The NIH-3T3, light II, 293T, and LS174T cells were purchased from the American Type Culture Collection (Manassas, VA, USA) and routinely cultured according to the guidelines from ATCC. The human fetal osteoblastic cell line hFOB1.19 was obtained from the Cell Bank of the Chinese Academy of Sciences (Shanghai, China). The human articular chondrocytes were purchased from Jining Shiye (Shanghai, China). SAG, JQ1, Pomalidomide, GDC-0449, DT2216, and ABT-263 were obtained from Selleck Chemicals (Houston, TX, USA). TNF-α, BAY-11-7082, H-89, MTT, and MG132 were purchased from Beyotime (Suzhou, China). PGE2 was obtained from Sigma-Aldrich (St. Louis, MO, USA).

### Plasmids

The TCF/LEF luciferase reporter plasmid, NF-κB luciferase reporter plasmid, and TK-*Renilla* luciferase plasmid were purchased from Promega (Madison, WI, USA). Smo plasmid was purchased from Origene (Rockville, MD, USA). The Smo mutant plasmids were generated from wild type Smo plasmid using QuickChange Site-Directed Mutagenesis kit from Agilent (Santa Clara, CA, USA) and confirmed by sequencing. Information on Smo mutants plasmids is provided in Supplementary data 2. The Gli1 plasmid and Gli2-HA plasmid were obtained from Addgene (Cambridge, MA, USA). Transient transfections were performed by Lipofectamine 2000 reagent from Invitrogen (Grand Island, NY, USA) according to the manufacturer's instructions.

### *In vitro* Cytotoxicity Assay

Cells were seeded in 96-well plates at an appropriate density and treated with compounds for 72 h. The effects on proliferation were determined using MTT according to the manufacturer's protocol.

### Real-time quantitative PCR (RT-qPCR)

Total RNA was extracted from cells or tissues using the NucleoSpin RNA kit (#740955.50, TaKaRa). RNA reverse transcriptions were performed with PrimeScript RT Reagent Kit with RNase inhibitor (#RR037B, TaKaRa). The quantitative PCR amplifications were performed in triplicate with TB Green Premix Ex Taq (Tli RNase H Plus) (#RR420A, TaKaRa) in an iCycler iQ system (Bio-Rad; Hercules, CA). Relative gene expression for interested genes was calculated using the 2^-ΔΔCt^ method and normalized to *mGUSB* expression. Primer sequences for RT-qPCR are as follows: *mGli1* forward (5′-GCAGTGGGTAACATGAGTGTCT-3′), reverse (5′-AGGCACTAGAGTTGAGGAATTGT-3′); *mPtch1* forward (5′-GCTACGACTATGTCTCTCACATCAACT-3′), reverse (5′-GGCGACACTTTGATGAACCA-3′); *mGUSB* forward (5′-CTGCCACGGCGATGGA-3′), reverse (5′-ACTGCATAATAATGGGCACTGTTG-3′).

### Dual luciferase reporter assay

Cells seeded in 96-well plates were treated with various treatments as indicated. The luciferase activity in the cell lysates was measured using Dual-Luciferase Reporter Assay System (Promega, Madison, WI, USA) according to the manufacturer's instructions in a luminometer (Molecular Devices, Sunnyvale, CA). The firefly luciferase values were normalized to *Renilla* values.

### Immunoblot analysis

Cells were lysed in cell lysis buffer (50 mM Tris pH 8.0, 150 mM NaCl, 10% glycerol, 1 mM EDTA, 50 mM NaF, and 0.1% NP-40) supplemented with protease and phosphatase inhibitors. Total protein extracts of tissues were generated in radioimmunoprecipitation (RIPA) assay lysis buffer containing complete protease and phosphatase inhibitor cocktail. Protein content was quantified using the Pierce BCA Protein Assay Kit (#23225, Thermo Fisher Scientific). The proteins were separated by SDS-PAGE, electrophoretically transferred to polyvinylidene difluoride (PVDF) blotting membranes (#LC2002, Thermo Fisher Scientific), and subjected to routine immunoblot analysis with primary antibodies as follows: anti-Bcl-2 (#15071), anti-Mcl-1 (#94296), anti-VHL (#68547), anti-CRBN (#71810), and anti-Sufu (#2522) were obtained from Cell Signaling Technology (Danvers, Massachusetts, USA); anti-Bcl-x_L_ (#ab32370), anti-Gli1 (#ab134906), and anti-Smoothened (#ab236465) were obtained from Abcam (Cambridge, CB2 0AX, UK); anti-GAPDH (#60004-1-Ig) and anti-β-tubulin (#10094-1-AP) were purchased from Proteintech Group, Inc. (Rosemont, IL 60018, USA). The results were quantified by densitometry using ImageJ software (NIH).

### Immunoprecipitation

Cells were lysed in immunoprecipitation lysis buffer (#87787, Thermo Fisher Scientific) supplemented with protease and phosphatase inhibitor cocktail. Cell lysates were incubated with the indicated antibodies at 4 °C overnight, followed by incubation with Protein A/G Plus-Agarose beads (Santa Cruz Biotechnology) at 4 °C for 4 h. Immunoprecipitates were collected by centrifugation and washed three times with washing buffer. The complex was separated from the beads and then boiled for 10 min. The immunoprecipitates and inputs were analyzed by SDS-PAGE and immunoblot using the antibodies as indicated.

### Immunofluorescence staining

Cells were fixed with 4% paraformaldehyde (PFA) for 15 min, permeabilized with 0.3% Triton X-100 in PBS for 10 min, and blocked with 1% BSA in PBS for 2 h. After that, the cells were further incubated with rabbit anti-Gli1 antibody (#101156-T10, SinoBiological) at 4 °C overnight. Then cells were stained with the Alexa Fluor®594-conjugated Goat Anti-rabbit IgG secondary antibody (red) for 1.5 h. Nuclei were counterstained with DAPI (blue). The immunofluorescent images of cells were captured under a fluorescence microscope (Leica).

### Fluorescence polarization

Competitive fluorescence polarization assays were carried out in black-walled plates in assay buffer (20 mM Na_2_HPO_4_ ·12H_2_O, 20 mM NaH_2_PO_4_·12H_2_O, 1 mM EDTA, 50 mM NaCl, 0.05% PF-68). The use of protein/peptide probe pairs was as follows: Bcl-x_L_ (5 nM) and FAM-Bad (5 nM), Bcl-2 (100 nM) and FAM-Bax (5 nM), Mcl-1 (100 nM) and FAM-Bim (5 nM). Assay buffer, proteins, drugs, and peptides were added to the assay plates sequentially. The assay plates were slightly shaken for 3 min and incubated for 30 min at room temperature. The fluorescence polarization was measured on a Synergy 2 reader (BioTek).

### Homogeneous Time-Resolved Fluorescence (HTRF) assay for ternary complex formation

Bcl-x_L_/CRBN ternary HTRF assay was used to monitor ternary complex formation between target protein, PROTAC and E3 ligase. Bcl-x_L_/CRBN PROTAC BINDING ASSAY KITS was purchased from Cisbio (#63ADK000CB37PEG). 200 nL compounds were transferred into 384 assay plate, followed by centrifuging at 1000 RPM. Then 5 µL Bcl-x_L_ and CRBN protein were added into each assay well. After incubation at 25 °C for 15 min, 10 µL Tag2-Eu&Tag1-d2 working solution was added into each assay well and incubated 25 °C for 120 min. The Relative HTRF value was read with BMG (PHERAstar FSX).

### *In vivo* mouse studies

Standard pharmacokinetic (PK) studies were conducted using male ICR Mice. A single 2 mg/kg intravenous (i.v.) injection, 10 mg/kg intraperitoneal (i.p.) injection or 10 mg/kg oral (p.o.) administration of SIAIS361034 was evaluated. Plasma concentrations of SIAIS361034 at each of the 7 time points (0.25 h, 0.5 h, 1 h, 2 h, 4 h, 8 h, and 24 h post dosing) are the average values from 3 animals.

For Depilatory model studies 37, male C57BL/6J mice (7 to 8 weeks of age) were purchased from Shanghai Lingchang Biotechnology Co. (Shanghai, China). After 2 days of acclimation, only healthy mice were used in the experiment. The back skin of the anesthetized mice was shaved and depilated using the depilatory Nair (Church & Dwight Co., Inc.). After 4 days, the depilated mice were randomized into groups for treatments as indicated. The back skin phenotypes were recorded by taking pictures every 3 days. Skin tissues were harvested 6 h after the last dosage to examining *Gli1* mRNA expression as well as Gli1 and Bcl-x_L_ protein levels.

For *in vivo* SmoA1 MB allograft studies, SmoA1 spontaneous MB tumors were obtained from *ND2:SmoA1* transgenic mice as previously described 38. Male nude mice (4 to 5 weeks of age) were purchased from Shanghai Lingchang Biotechnology Co. (Shanghai, China). Spontaneous tumors were harvested and subcutaneously allografted into nude mice. When the tumor volumes reached approximately 2000 mm^3^, they were harvested and further subcutaneously allografted into nude mice. When the tumor volumes reached about 150 to 200 mm^3^, the tumor-bearing mice were randomly assigned into vehicle and treatment groups. Mice were weighed every 3 days. Tumor size was measured once every 3 days using microcaliper. Tumor volume (V) was determined using the following formula: V = (*L×W*^2^) ×0.5, where *L* is length, and *W* is width in mm. Tumor tissues were harvested 6 h after the last dosage to examining *Gli1* mRNA expression and protein levels. SIAIS361034 for i.p. administration was formulated in 0.5% sodium carboxymethyl cellulose (CMC-Na). All *in vivo* studies above were approved by and conformed to the policies and regulations of the Animal Care and Use Committee of Fudan University, China.

### PET-CT

Skeleton Micro-CT images of mice were taken under anesthesia. The femurs were fixed and stored in 4% PFA at 4 °C. Mice and femurs were scanned using Inveon PET-CT (Siemens). Bone tissues were computed with Inveon Acquisition Workplace (IAW). Image data were visualized and analyzed by Inveon Viewer.

### Histopathology of bone tissues

Femurs were fixed in 4% PFA and decalcified in 14% EDTA for 2 weeks at 4 °C. Bone tissues were embedded in paraffin. Thin sections were stained with hematoxylin and eosin (H&E) according to the manufacturer's instructions. For immunohistochemistry (IHC), paraffin-embedded sections of femurs were baked at 60 °C for 20 min, deparaffinized by xylene for 25 min, rehydrated through ethanol series, and incubated with citrate buffer at 95 °C for 20 min for antigen retrieval. Sections were incubated with the primary antibodies as indicated overnight at 4 °C, followed by goat anti-rabbit secondary antibody. The primary antibodies used in this study include Bcl-x_L_ (1:2000, ab178844, Abcam) and Gli1 (1:100, 101156-T10, Sino Biological).

### Platelet toxicity assays

7 weeks of age C57BL/6J mice (Shanghai Lingchang Biotechnology Co.) were treated with single i.p. doses of SIAIS361034 (50 and 100 mg/kg) or single p.o. doses of ABT-263 (100 mg/kg). Blood was collected at different time points via retro-orbital bleeding in anticoagulation tube. Platelets or different blood cells were enumerated using BM800 blood analyzer (Bowlinman Sunshine).

### Statistical analysis

All the statistical analyses in this study were performed using GraphPad Prism 8 software (La Jolla, CA, USA). *P* values were calculated using one-way analysis of variance (ANOVA) (more than two groups) or two-sided unpaired Student's *t*-test (two groups). *P* value less than 0.05 was considered statistically significant. Asterisks denote statistical significance (^*^P < 0.05; ^**^P < 0.01; ^***^P < 0.001; ns, not significant).

### Data availability

The data generated in this study are available within the article and its Supplementary data files.

## Results

### SIAIS361034 is a selective Bcl-x_L_ PROTAC

**Figure 1:**
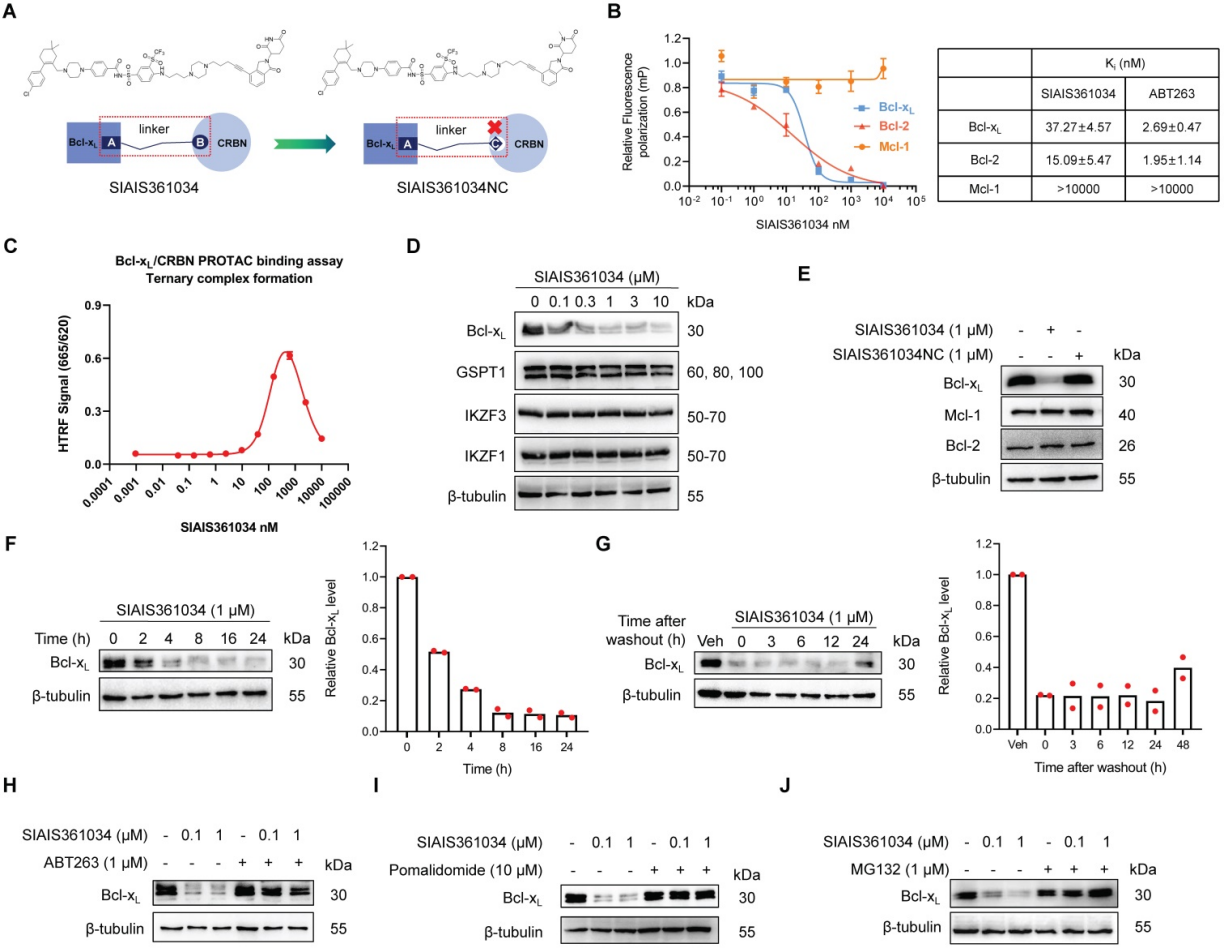
** SIAIS361034 is a selective Bcl-x_L_ PROTAC. (A)** The chemical structures of SIAIS361034 and negative control SIAIS361034NC showing a dual Bcl-2/Bcl-x_L_ ligand linked to a CRBN ligand via an optimized linker. SIAIS361034NC has the inactive CRBN ligand that does not bind to CRBN E3 ligase.** (B)** Binding affinities of SIAIS361034 and ABT-263 against Bcl-x_L_, Bcl-2, and Mcl-1 were measured by Fluorescence Polarization assay and are represented in terms of inhibition constant (K_i_). **(C)** Ternary complex formation of Bcl-x_L_ with SIAIS361034 and CRBN E3 ligase, determined by HTRF assay. Data represent mean ± SD (n = 2).** (D)** A representative western blot analysis of Bcl-x_L_, GSPT1, IKZF1, and IKZF3 in NIH-3T3 cells after being treated with SIAIS361034 as indicated for 24 h. **(E)** A representative western blot analysis of Bcl-x_L_, Bcl-2, and Mcl-1 in NIH-3T3 cells after being treated with SIAIS361034 (1 µM) and SIAIS361034NC (1 µM). **(F)** A representative western blot analysis of Bcl-x_L_ expression in NIH-3T3 cells after they were treated with SIAIS361034 (1 µM) for varied durations as indicated. Densitometric analysis of Bcl-x_L_ expressions is presented on the right. Each bar represents percentage data relative to 0 h. **(G)** A representative western blot analysis of Bcl-x_L_ expression in NIH-3T3 cells after they were treated with SIAIS361034 (1 µM) for 24 h followed by drug withdrawal and cultured for various durations as indicated without SIAIS361034. Densitometric analysis of Bcl-x_L_ expressions is presented on the right. Each bar represents percentage data relative to Vehicle. **(H)** Pretreatment with ABT-263 (1 µM) reverses the Bcl-x_L_ degradation induced by SIAIS361034. A representative western blot analysis of Bcl-x_L_ is shown. The NIH-3T3 cells were untreated or pretreated with ABT-263 for 2 h and then were treated with or without SIAIS361034 as indicated for 24 h. **(I, J)** Pretreatment with Pomalidomide (10 µM) or MG132 (1 µM) reverses the Bcl-x_L_ degradation induced by SIAIS361034. A representative western blot analysis of Bcl-x_L_ is shown. The NIH-3T3 cells were untreated or pretreated with Pomalidomide or MG132 for 2 h and then were treated with or without SIAIS361034 as indicated for 24 h. β-tubulin was used as a protein loading control in all western blot analyses shown in **D**, **E**, **F**, **G**, **H**,** I**, and** J**.

To assess whether Bcl-x_L_ is a promising therapeutic target for inhibiting Hh pathway activity, we reasonably designed and synthesized a series of PROTAC degraders targeting Bcl-x_L_ to CRBN E3 ubiquitin ligase through linking a dual Bcl-2/Bcl-x_L_ ligand derived from ABT-263 to a CRBN ligand and also screened the linker length via structure-activity relationship (SAR) study. Among those degraders, SIAIS361034, which had an extremely valid and specific degradation effect on Bcl-x_L_, was selected as the compound for this study. SIAIS361034NC was synthesized as a negative control of SIAIS361034 by changing the CRBN E3 ubiquitin ligase ligand in SIAIS361034 from an active state to an inactive state (Figure 1A and Figure S1A-C). We first measured the binding affinity of SIAIS361034 with Bcl-2 family proteins using Fluorescence polarization. The results showed that SIAIS361034 bound Bcl-x_L_ and Bcl-2 with K_i_ values of 37.27 nM and 15.09 nM, respectively, but exhibited little affinity to Mcl-1 (Figure 1B). Furthermore, the HTRF assay revealed that SIAIS361034 could form a stable ternary complex with Bcl-x_L_ and CRBN E3 ligase (Figure 1C). These results suggest that SIAIS361034 functions as a PROTAC. We next evaluated the effects of SIAIS361034 on the expression of Bcl-x_L_ in NIH-3T3 cells that respond well to the activation of the Hh signaling pathway 39. As shown in Figure 1D, SIAIS361034 dose-dependently degraded Bcl-x_L_ with a maximum degradation (D_max_) of 88.09% and exerted almost no effect on the Bcl-x_L_ mRNA levels (data not shown). SIAIS361034 did not affect abundance of all four known CRBN neo-substrates, including Ikaros family zinc finger proteins 1 (IKZF1) and 3 (IKZF3), developmental transcription factor SALL4, and the translation termination factor GSPT1, demonstrating that SIAIS361034 is a specific Bcl-x_L_ PROTAC with minimal off-target degradation 40-42 (Figure 1D and Figure S1D). Our analysis also revealed that SIAIS361034 significantly degraded Bcl-x_L_ and did not change the levels of Bcl-2 and Mcl-1 (Figure 1E). Furthermore, SIAIS361034NC, which is not able to bind to CRBN, failed to degrade Bcl-x_L_ (Figure 1E). SIAIS361034 degraded the Bcl-x_L_ in a time-dependent manner, and this degradation may last around 48 h (Figure 1F-G). We also observed that preincubation with ABT-263 abolished the SIAIS361034-induced Bcl-x_L_ degradation (Figure 1H). These results showed that SIAIS361034 is a specific PROTAC degrader targeting Bcl-x_L_. We used CRBN inhibitor Pomalidomide 43 and proteasome inhibitor MG132 to further confirm that SIAIS361034 induces Bcl-x_L_ degradation through CRBN and proteasome. We observed that both CRBN inhibitor Pomalidomide (Figure 1I) and proteasome inhibitor MG132 (Figure 1J) obviously abolished SIAIS361034-induced Bcl-x_L_ degradation, further confirming that SIAIS361034 acts as a PROTAC degrader using CRBN E3 ligase and proteasome system to degrade Bcl-x_L_. Co-immunoprecipitation assay revealed that SIAIS361034 induce Bcl-x_L_ ubiquitination (Figure S1E). Collectively, these data demonstrated that SIAIS361034 is a selective Bcl-x_L_ PROTAC that potently degrades Bcl-x_L_ via CRBN E3 ubiquitin ligase and ubiquitin proteasome system.

### SIAIS361034 significantly and selectively inhibits Hh pathway activity *in vitro*

**Figure 2:**
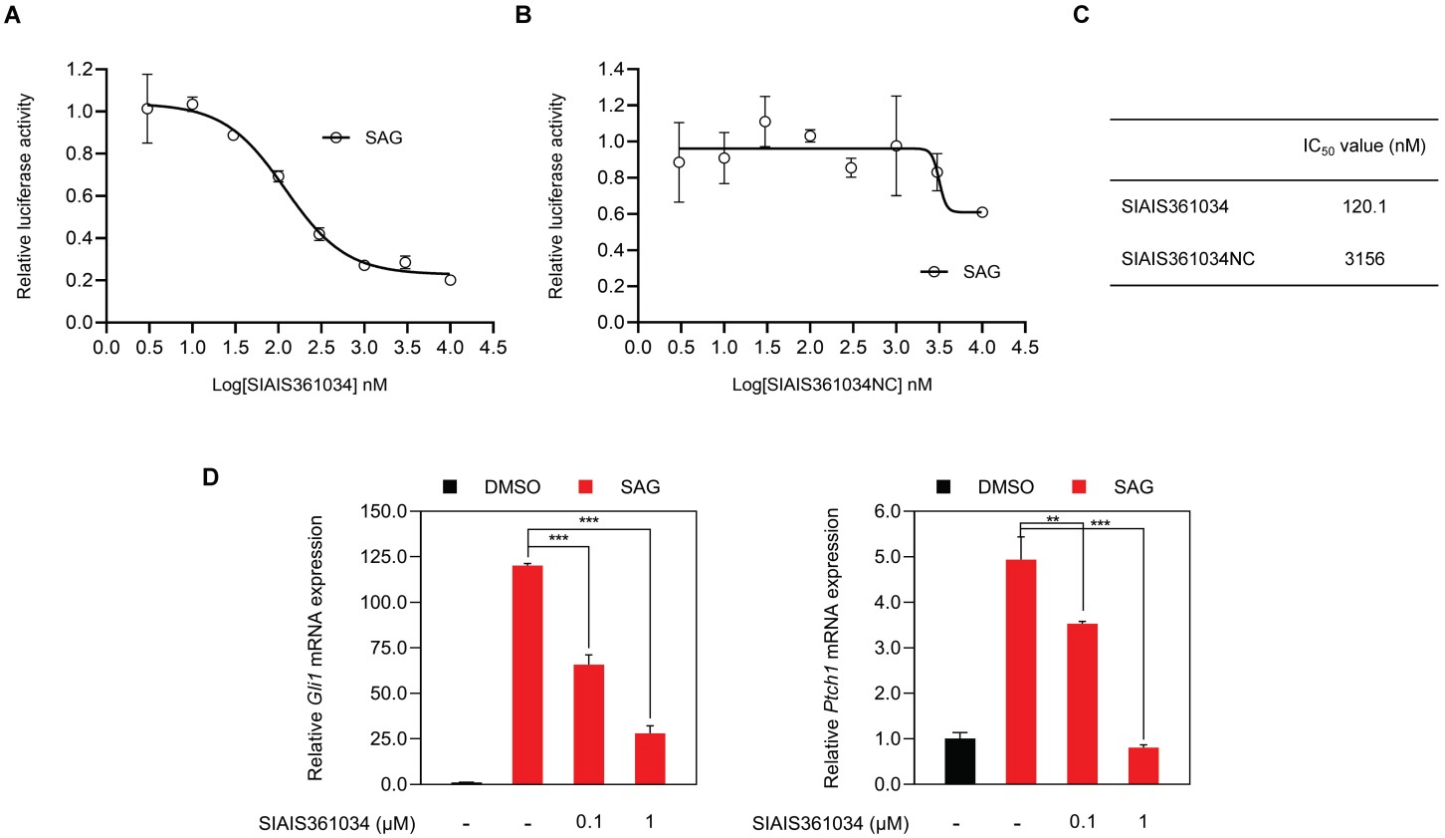
** SIAIS361034 significantly and selectively inhibits Hh pathway activity *in vitro*. (A, B)** Dual-luciferase reporter analysis of the inhibitory effect of SIAIS361034 and SIAIS361034NC against the Hh activity in light II cells exposed to SAG (100 nM) with or without various concentrations of SIAIS361034 and SIAIS361034NC as indicated for 36 h. Data represent mean ± SD (n = 2).** (C)** IC_50_ values of SIAIS361034 and SIAIS361034NC inhibiting the Gli-luciferase activity provoked by SAG. IC_50_ values were determined by nonlinear regression dose response fit in Graphpad prism 8. **(D)** RT-qPCR analysis of the effect of SIAIS361034 on the mRNA expression of Hh pathway target genes *Gli1* and *Ptch1* in the NIH-3T3 cells exposed to SAG with or without SIAIS361034 for 36 h as indicated. Data represent mean ± SD (n = 3).

Wu et al. have reported that BH3 mimetics inhibit Hh activity 31. To evaluate whether SIAIS361034 may inhibit the Hh pathway activity, we used SAG, a small molecule Smo agonist 44, to stimulate the Gli transcriptional factor activity in light II reporter cells, an NIH-3T3 cell line with stable expression of Gli-luciferase and TK-Renilla reporter genes 45. We found that SIAIS361034 obviously suppressed the activity of the Hh pathway provoked by SAG with an IC_50_ value of 120.1 nM (Figure 2A, 2C), while SIAIS361034NC exhibited no effect on the Hh signaling pathway, as reflected by its IC_50_ value of 3156 nM (Figure 2B-C). To further confirm the ability of SIAIS361034 on suppressing the Hh signaling pathway, we examined its effect on the mRNA expression of Hh target genes *Gli1* and *Ptch1*, which served as readout of the Hh pathway activity 46, in NIH-3T3 cells. Consistent with the results of Gli-luciferase assays, SIAIS361034 significantly blocked the expression levels of Hh target genes, *Gli1* and *Ptch1*, activated by SAG (Figure 2D). *In vitro* cytotoxicity assay showed that SIAIS361034 exhibited no toxic effects when it acted on light II and NIH-3T3 cells at a concentration of 10 μM for 72 h, suggesting the inhibitory effect of SIAIS361034 on Hh activity is not due to its cytotoxicity (Figure S2A). Collectively, our data indicated that SIAIS361034 may significantly inhibit the Hh signaling pathway *in vitro*.

To date, more and more evidence has shown the cross-talk between Hh signaling pathway and other tumor-related signaling pathways, such as NF-κB and Wnt signaling pathway, which in turn affect the activity of GLI transcription factor 47-49. To rule out the possibility that SIAIS361034 nonspecifically inhibits the Hh pathway activity, we next used the dual luciferase reporter assay to examine the effects of SIAIS361034 on other signaling pathways, such as NF-κB and Wnt signaling pathways 47-49. BAY 11-7082, a positive inhibitor of the NF-κB signaling pathway, significantly impeded the activity of NF-κB luciferase activated by TNF-α, while SIAIS361034 exhibited no inhibitory activity at a concentration of 10 μM (Figure S2B). We also found that SIAIS361034 failed to repress the activity of the Wnt signaling pathway activated by PGE2. In contrast, the Wnt signaling pathway positive inhibitor H-89 significantly inhibited the TCF/LEF luciferase activity, as shown in Figure S2C. Overall, these results indicated that SIAIS361034 may selectively inhibit the Hh signaling pathway.

### SIAIS361034 inhibits the Hh signaling pathway by disabling Bcl-x_L_/Sufu interaction

**Figure 3:**
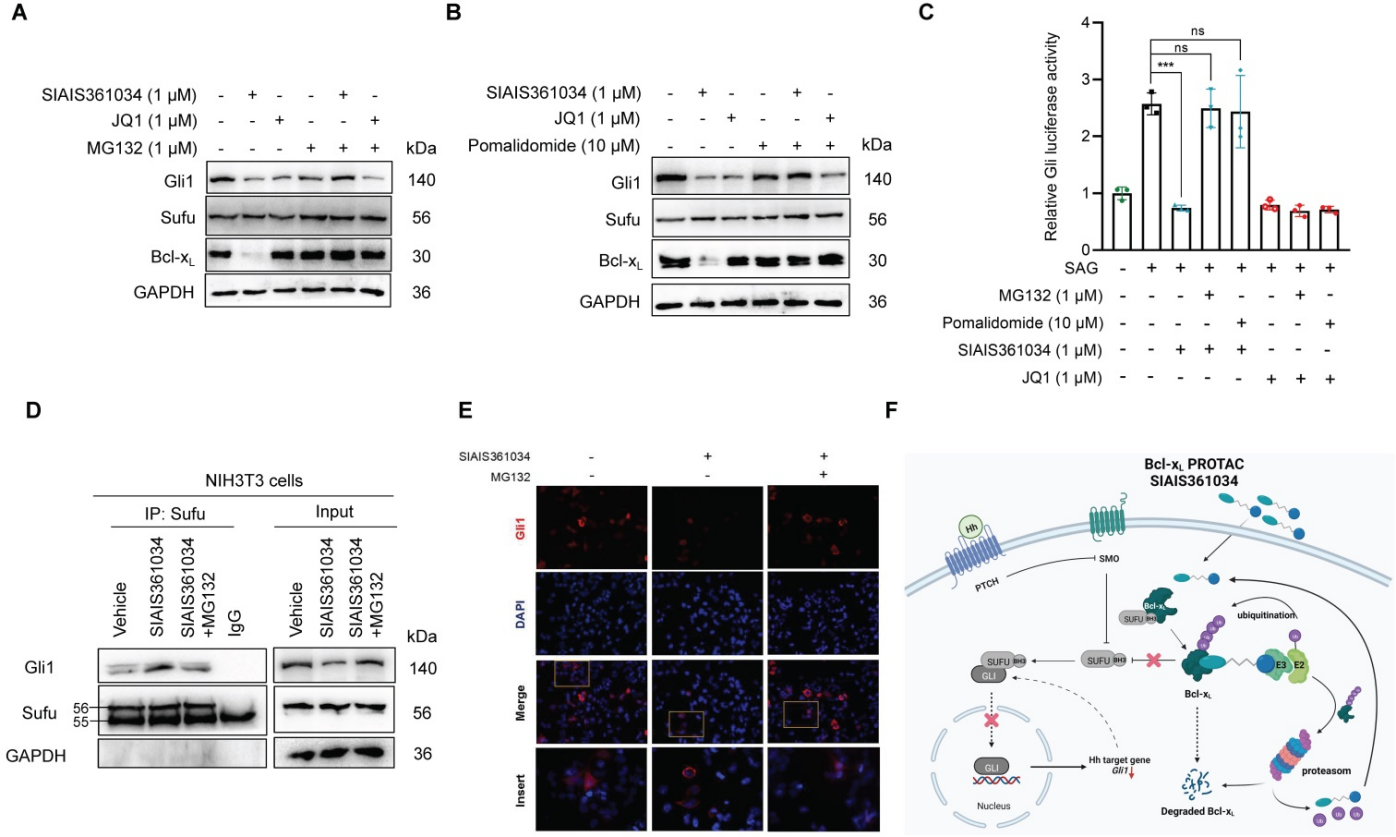
** SIAIS361034 inhibits the Hh signaling pathway by disabling pro-survival Bcl-2 family proteins/Sufu interactions. (A, B)** A representative western blot analysis of Gli1, Bcl-x_L_, and Sufu expression in light II cells pretreated with or without MG132 (1 µM) or Pomalidomide (10 µM) for 2 h, followed by treatment with or without SIAIS361034 (1 µM) and JQ1 (1 µM). **(C)** Dual-luciferase reporter analysis of the activity of SIAIS361034 against the Hh activity in light II cells exposed to SAG (100 nM) pretreated with or without MG132 (1 µM) or Pomalidomide (10 µM) for 2 h, followed by treatment with or without SIAIS361034 (1 µM) and JQ1 (1 µM). Data represent mean ± SD (n = 3). **(D)** A representative western blot analysis of Gli1 after immunoprecipitation with Sufu in NIH-3T3 cells pretreated with or without MG132 (1 µM) for 2 h, followed by treatment with or without SIAIS361034 (1 µM) and whole-cell lysates (Input) are shown from a single experiment. **(E)** Representative IF staining of Gli1 in 293T cells that were transfected with Gli1 and then were treated with or without SIAIS361034 (1 µM) and MG132 (1 µM) as indicated for 24 h.** (F)** Model of SIAIS361034 antagonizing Gli1 through degradation of Bcl-x_L_. This model image was created in BioRender.com. GAPDH was used as protein loading control in western blot analysis shown in **A**, **B**, and **D**.

Having demonstrated that SIAIS361034 may specifically inhibit the Hh pathway, we next set out to dissect its putative molecular mechanism for inhibiting Hh activity. Wu et al. previously reported that BH3 mimetics suppress Gli activity by disabling pro-survival Bcl-2 family proteins/Sufu interactions, therefore, enabling the engagement of Sufu with Gli and consequently prohibiting the translocation of Gli into nucleus 31. In this context, we hypothesized that SIAIS361034 may suppress the Hh signaling pathway by disabling pro-survival Bcl-x_L_/Sufu interactions via degrading Bcl-x_L_, similar to BH3 mimetics. To this end, we first investigate whether the inhibitory effect of SIAIS361034 on Hh pathway is mediated by the degradation of Bcl-x_L_. As expected, preincubation of MG132 and Pomalidomide (Figure 3A-B) retained the decreased Gli1 protein level caused by exposure to SIAIS361034. However, MG132 and Pomalidomide failed to recover the reduced Gli1 protein elicited by the BET inhibitor JQ1 (Figure 3A-B), which inhibits Hh pathway activity by decreasing the transcription of Gli 50. These observations were further confirmed by Gli-luciferase assay, MG132 and Pomalidomide also abolished the suppressed Gli-luciferase activity elicited by SIAIS361034, whereas no significant differences were found in the Gli-luciferase activity when treated with JQ1 alone, in combination with MG132 or Pomalidomide (Figure 3C). Collectively, these data indicated that SIAIS361034 may inhibit Hh activity by a mechanism of degradation for Bcl-x_L_ and consequently inhibition of Hh activity.

Considering that BH3 mimetics suppress Gli activity by disabling pro-survival Bcl-2 family proteins/Sufu interaction as previously reported 31, we further addressed whether SIAIS361034 may interfere with the interaction of Sufu with Gli1. Co-immunoprecipitation assay revealed that treatment with SIAIS361034 increased the interaction of Sufu with Gli1, whereas preincubation with MG132 abrogated this effect (Figure 3D). Immunofluorescence staining with Gli1 antibodies revealed that SIAIS361034 suppressed translocation of Gli1 from cytoplasm into nucleus (Figure 3E). These observations suggest that SIAIS361034 inhibits Hh activity by degrading Bcl-x_L_, thus relieving Sufu from Bcl-x_L_/Sufu interaction and allowing its binding with Gli1, therefore repressing translocation of Gli1 from cytoplasm into nucleus (Figure 3F).

### SIAIS361034 possesses the ability of combating resistance to Smo inhibitors caused by *Smo* mutations and *Gli2* amplification

**Figure 4:**
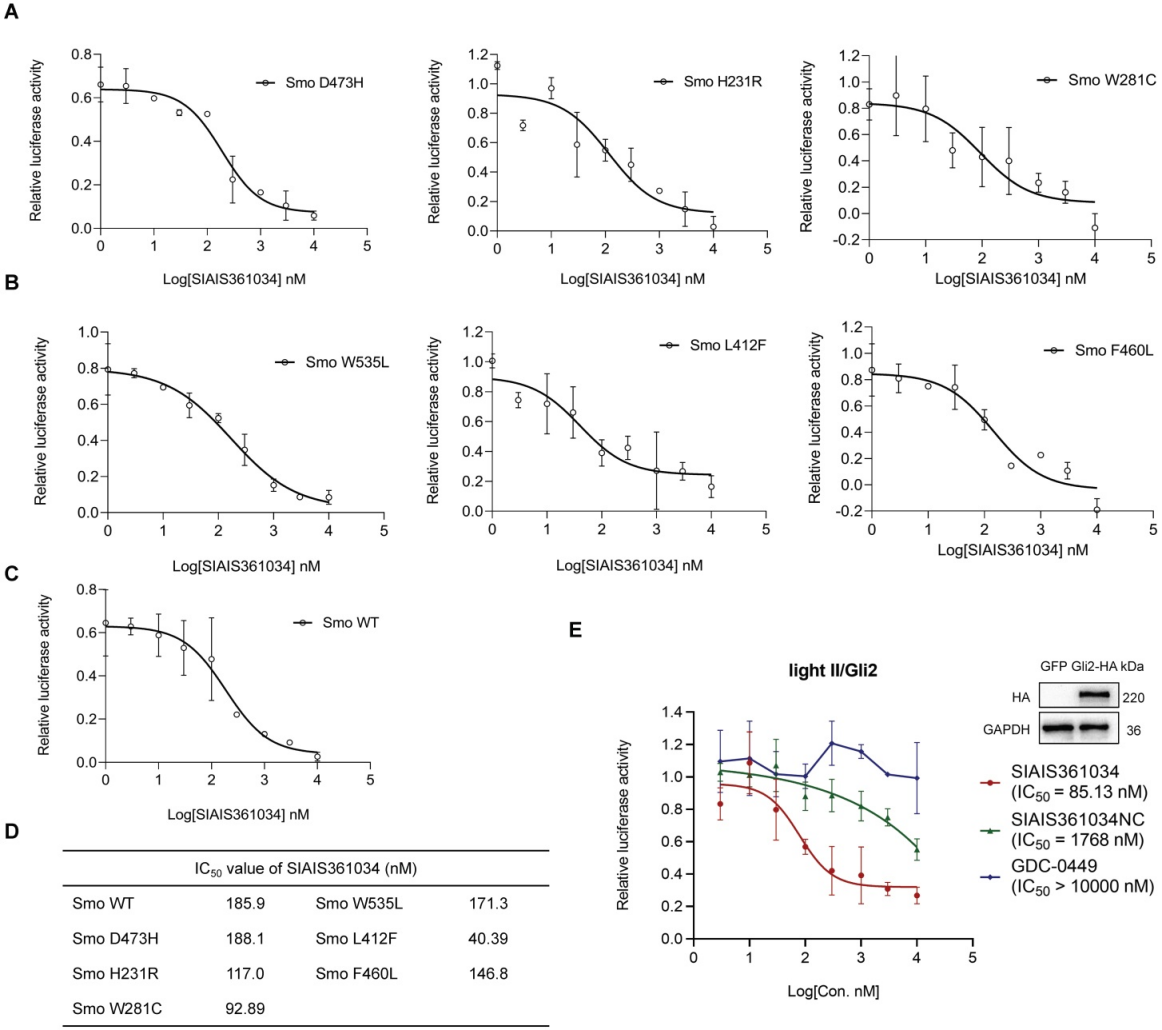
** SIAIS361034 possesses the ability to combat resistance to Smo inhibitors caused by *Smo* mutations and *Gli2* amplification. (A**-**C)** Dual-luciferase reporter analysis of the activity of SIAIS361034 against the Hh activity induced by ectopic expression of Smo D473H (**A**), Smo H231R (**A**), Smo W281C (**A**), Smo W535L (**B**), Smo L412F (**B**), Smo F460L (**B**), Smo WT (**C**) in light II cells, followed by treatment with various concentrations of SIAIS361034 as indicated for 36 h. Data represent mean ± SD (n = 2). **(D)** IC_50_ values of the inhibitory effect of SIAIS361034 on the dual-luciferase activity measured in Figure 4A-C. IC_50_ values were determined by nonlinear regression dose response fit in Graphpad prism 8. **(E)** Dual-luciferase reporter analysis of the inhibitory effect of SIAIS361034, SIAIS361034NC, and GDC-0449 on the Hh activity initiated by ectopic expression of Gli2-HA in light II cells, followed by treatment with various concentrations of SIAIS361034, SIAIS361034NC, and GDC-0449 as indicated for 36 h. Data represent mean ± SD (n = 2).

*Smo* mutations within its ligand binding pocket or in its pivotal residue, as well as amplification of *Gli2* are the predominant mechanisms responsible for the resistance to current Smo antagonists 20, 21, 23, 51. Having demonstrated that SIAIS361034 effectively inhibits the Hh activity by degrading Bcl-x_L_, we thus tested whether SIAIS361034 may overcome the drug resistance caused by *Smo* mutants and *Gli2* amplification. To verify this hypothesis, we first respectively transfected different Smo mutants plasmids into light II cells and tested the effect of SIAIS361034 on Hh activity provoked by these ectopic plasmids. The overexpression efficiency of ectopic plasmids was confirmed by western blot analysis (Figure S3A). SIAIS361034 obviously inhibited the Hh activity provoked by wild type Smo plasmid and various Smo mutants containing mutations in its ligand binding pocket 19, D473H, H231R, and W281C (Figure 4A), or mutations in its pivotal residue 52, W535L, L412F, and F460L (Figure 4B) in a dose-dependent manner. However, little appreciable effect was observed with Smo inhibitor GDC-0449 (Figure S3B-D). What stands out in these results is that no significant IC_50_ shift was observed for SIAIS361034 inhibiting Gli-luciferase stimulated by ectopic expressions of Smo wild-type and mutations (Figure 4C-D). We also observed that SIAIS361034 exhibited significant potency against Hh activity evoked by ectopic expression of *Gli2*. By contrast, administration of GDC-0449 had no effect on the Hh activity in response to *Gli2* amplification (Figure 4E). Taken together, these data suggested that SIAIS361034 is able to combat the resistance to current Smo inhibitors caused by *Smo* mutations and *Gli2* amplification.

### SIAIS361034 retards hair growth in a depilatory model by inhibiting the Hh pathway activity

**Figure 5:**
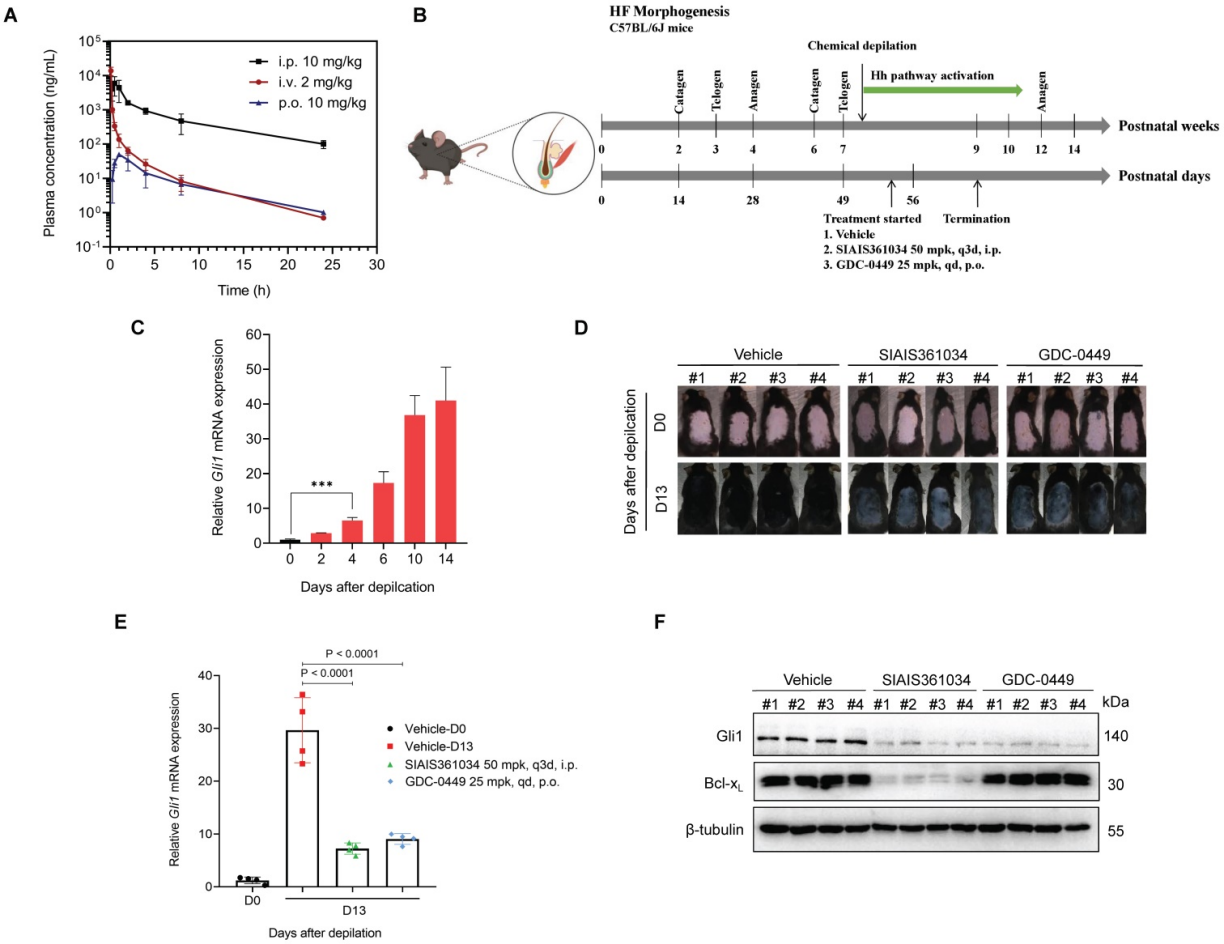
** SIAIS361034 retards hair growth in a depilatory model by inhibiting the Hh pathway activity. (A)** Plasma concentrations of SIAIS361034 after a single 2 mg/kg intravenous (i.v.) injection, 10 mg/kg intraperitoneal (i.p.) injection or 10 mg/kg oral (p.o.) administration. Data represent mean ± SD (n = 3).** (B)** Illustration of the experimental design of depilatory model. This model image was created in BioRender.com. **(C)** RT-qPCR was carried out to determine *Gli1* expression in the skin samples from mice on indicated days after depilatory treatment. Data represent mean ± SD (n = 3). **(D)** The gross phenotype of the back skin of the mice during the administration period. Pictures of mice are shown (n = 4). **(E)** The depilatory model mice were sacrificed for skin tissues 6 h after the last drug application. RT-qPCR was carried out to determine *Gli1* expression in the skin samples from mice. Data represent mean ± SD (n = 4). **(F)** A representative western blot analysis of Gli1 and Bcl-x_L_ in the skin samples from the mice at the end of the experiment (n = 4). β-tubulin was used as a protein loading control.

First, we assessed *in vivo* pharmacokinetic properties of SIAIS361034 in mice. Following a single 10 mg/kg intraperitoneal (i.p.) or 2 mg/kg intravenous (i.v.) injection of SIAIS361034, good plasma exposure of the compound was observed over 24 h. However, SIAIS361034 has a poor pharmacokinetic property via 10 mg/kg oral (p.o.) administration (Figure 5A, Table S1). Hence, we used i.p. to deliver SIAIS361034 for further *in vivo* study.

Hh signaling pathway is essential for the onset of the anagen phase of hair follicle (HF) morphogenesis and consequently for the formation of hair and proper control of HF morphogenesis 53, 54. To examine whether the potent *in vitro* ability of SIAIS361034 to inhibit Hh activity can be translated into *in vivo* efficacy, we established a depilatory mouse model by using chemical depilation to damage the hair follicles 37 (Figure 5B). As shown in Figure 5C, chemical cream-induced depilation significantly increased *Gli1* expression by day 4, and this increase in *Gli1* mRNA levels was maintained through day 14, indicating the Hh pathway activation during the hair growth. To determine whether SIAIS316034 can block Hh pathway activation and consequently retard hair growth in this model, SIAIS316034 and GDC-0449 were administered after 4 days of depilation. SIAIS316034 and GDC-0449 treatment significantly retarded hair growth during depilation-induced hair regeneration, and meanwhile, the hair of the vehicle group had completely returned to the level before depilation at the termination of administration (Figure 5D). Meanwhile, the inhibition of hair growth was comparable to the reduction in Hh activity, as evidenced by alterations in the expression of *Gli1* mRNA (Figure 5E), suggesting that SIAIS316034 suppressed the Hh activity and consequently the hair growth. To further confirm the effect of SIAIS361034 on hair growth and Hh activity associated with its degradation on Bcl-x_L_, we tested the Gli1 and Bcl-x_L_ protein levels in skin tissues. As is shown in Figure 5F, SIAIS361034 suppressed the expression of Gli1 and Bcl-x_L_, while GDC-0449 showed no effect on the levels of Bcl-x_L_. These findings supported that SIAIS361034 can block the Hh signaling pathway activation *in vivo* in the depilatory model by degrading Bcl-x_L_.

### SIAIS361034 potently inhibits the growth of medulloblastoma tumor carrying W539L mutation in Smo (Smo W539L; SmoA1)

**Figure 6:**
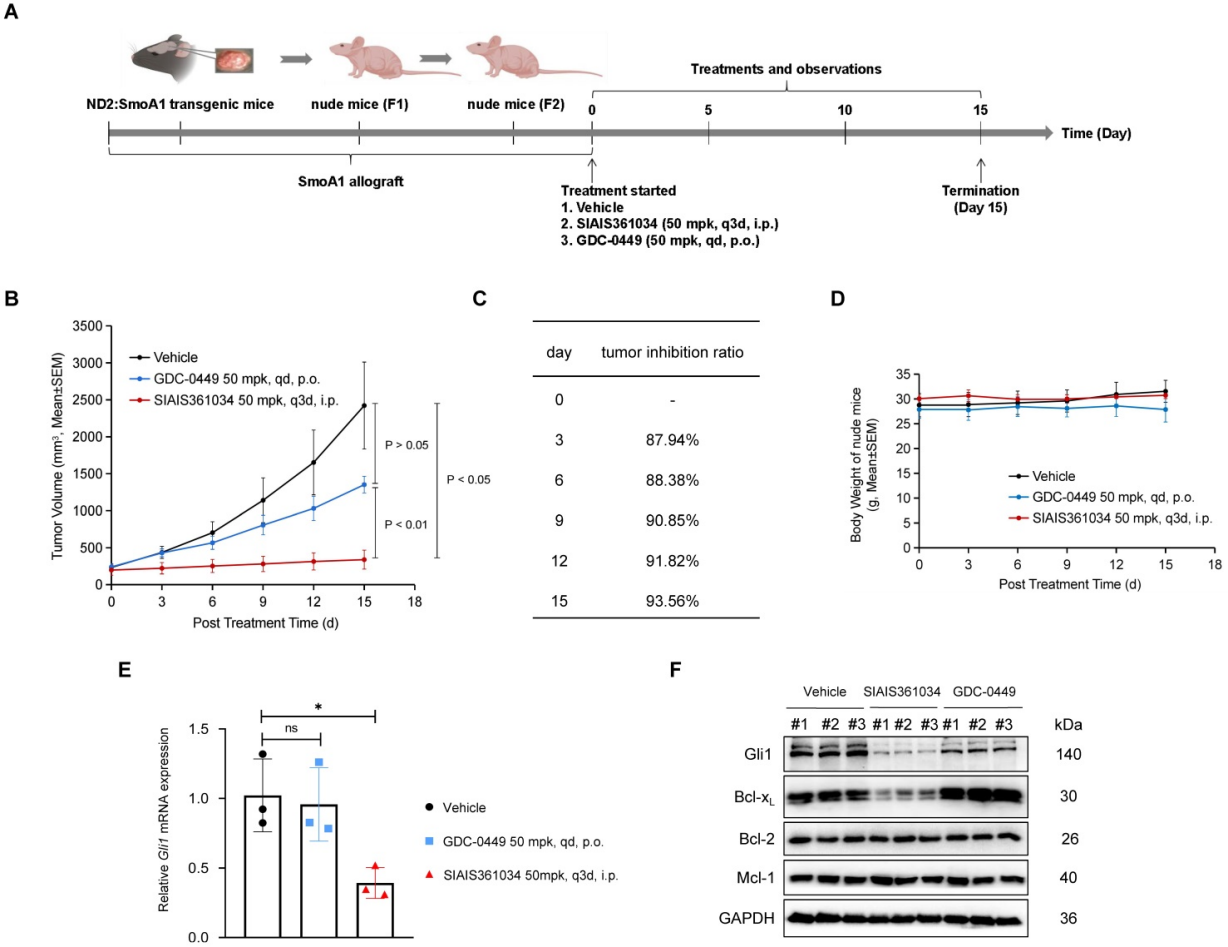
**SIAIS361034 potently inhibits the growth of medulloblastoma tumor carrying W539L mutation in Smo (Smo W539L; SmoA1). (A)** Illustration of the experimental design of SmoA1 MB xenografts model. This model image was created in BioRender.com. **(B)** Subcutaneous tumor growth in the SmoA1 MB xenografts tumor model treated with a single i.v. dose of vehicle or SIAIS361034 50 mg/kg and p.o. dose of GDC-0449 50 mg/kg. Tumor volumes are presented as mean ± SEM (n = 3). **(C)** Growth inhibition rate in the SmoA1 MB xenografts tumor model. **(D)** Body weight changes of mice during the administration period as shown in Figure 6B. Data represent mean ± SEM (n = 3). **(E)** RT-qPCR was carried out to determine *Gli1* expression in the harvested tumors. Data represent mean ± SD (n = 3). **(F)** A representative western blot analysis of Gli1, Bcl-x_L_, Bcl-2, and Mcl-1 in the harvested tumors at the end of the experiment (n = 3). GAPDH was used as a protein loading control.

To further evaluate the potency of SIAIS361034 in combating the resistance to Smo inhibitors *in vivo*, we isolated the spontaneous medulloblastoma from *ND2:SmoA1* transgenic mice, which carry a mutation of *Smo W539L* under the control of ND2 promoter 38, and allografted them into the nude mice. Nude mice harboring *ND2:SmoA1* medulloblastoma were treated with SIAIS361034 or GDC-0449 as indicated in Figure 6A. The tumor growth was remarkably suppressed by administration with SIAIS316034, resulting in 93.56% mean tumor growth inhibition (TGI) ratio at the termination of the experiment. However, GDC-0449 at the dose of 50 mg/kg, a concentration that can achieve 100% TGI in medulloblastoma sensitive to Smo inhibitors 14, showed obvious resistance to the growth of SmoA1 tumors (Figure 6B-D). Moreover, consistent with the ability of SIAIS361034 and GDC-0449 against tumor growth, RT-qPCR analysis revealed that SIAIS316034 significantly inhibited the expression of *Gli1* mRNA in tumor tissues, while no alteration of the *Gli1* mRNA levels was observed in tumors exposed to GDC-0449 (Figure 6E). These observations indicated that SIAIS316034 suppressed the growth of SmoA1 tumors by inhibiting the Hh signaling pathway. Furthermore, we found that the potent antitumor activity of SIAIS316034 corresponded well to its ability of inducing Bcl-x_L_ degradation and repressing Gli1 expression (Figure 6F). Therefore, these observations revealed that SIAIS316034 possesses the ability of combating resistance to Smo inhibitors caused by *Smo* mutations *in vivo*.

### SIAIS361034 has no impact on the platelets and bone growth

**Figure 7:**
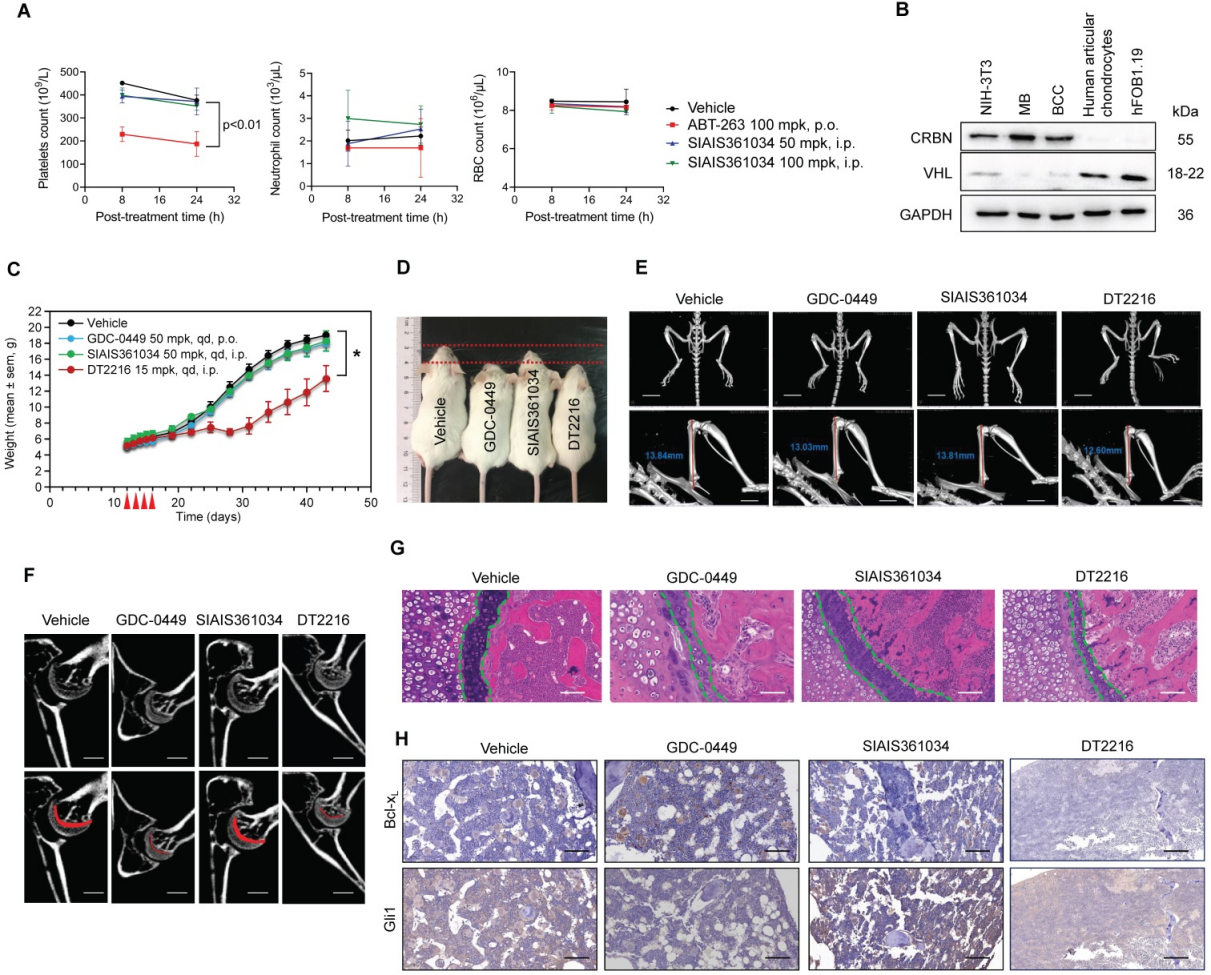
** SIAIS361034 has no impact on the platelet and bone growth. (A)** Numeration of platelets, neutrophils, and red blood cells at 8 h and 24 h after a single i.p. injection with SIAIS361034 or p.o. dosing with ABT-263 at the indicated doses. Data represent mean ± SD (n = 3). **(B)** A representative western blot analysis of CRBN and VHL in different cell lines and solid tumors. GAPDH was used as a protein loading control. **(C)** Changes in body weight in mice after the start of treatment with vehicle, SIAIS361034, GDC-0449, and DT2216. Data represent mean ± SEM (n = 4). Red arrows indicate the treatment with drugs. **(D)** Photograph of 5-week-old mice after drug treatment from P12 to P16. **(E)** Micro-CT analyses of overall skeleton and femurs of 6-week-old mice. Vertical red lines indicate the length of the femur. Scale bars indicate 10 mm (up) and 5 mm (down). **(F)** Longitudinal cross-section Micro-CT images of the femurs (up). Regions of growth plate were represented by the red area (down). Scale bars indicate 500 µm. **(G)** H&E staining of longitudinal cross-sections of femurs. The boundary of the growth plate was represented by green dashed lines. Scale bars indicate 100 µm. **(H)** IHC staining of longitudinal cross-sections of femurs. Scale bars indicate 100 µm.

It has been shown that Bcl-x_L_ is essential for the survival of platelets 55, 56, thus indicating the challenge of targeting Bcl-x_L_ with small molecules due to on-target toxicity of thrombocytopenia. Zhang et al. recently reported that targeting Bcl-x_L_ with PROTAC using CRBN E3 ubiquitin ligase may combat this on-target toxicity of thrombocytopenia due to their rare expression in platelets 36. Hence, it is conceivable that plates should be spared when treated with SIAIS361034. As anticipated, distinct doses of SIAIS361034 resulted in no appreciable alterations in the number of platelets, whereas the Bcl-2/Bcl-x_L_ dual inhibitor ABT-263 caused severe thrombocytopenia in mice after 8 h of administration (Figure 7A). In addition, SIAIS361034 showed no influence on other blood cells, such as neutrophils and red blood cells (RBC) (Figure 7A). These results indicated that SIAIS361034 is a platelet-sparing Bcl-x_L_ degrader.

Osteoblast differentiation and endochondral ossification are responsible for bone formation and resorption and are regulated by Hh signaling pathways 57. The impact of current Smo inhibitors on bone growth largely restricts their use in children patients 29, 30. By analyzing the RNA-sequencing data of human chondrocytes, we found that CRBN is rarely expressed in chondrocytes 58. This finding was confirmed by immunoblotting experiment, which revealed that CRBN expression was barely detectable in chondrocytes and osteoblasts (Figure 7B). However, VHL, another E3 ligase frequently used in PROTACs, was abundantly expressed in chondrocytes and osteoblasts (Figure 7B). These observations indicate the possibility that SIAIS361034 may spare permanent skeleton damage. To this end, we analyzed the effects of SIAIS361034 on bone morphology and structure after continuous administration of the young mice with SIAIS361034 from postnatal day 12 (P12) to postnatal day 16 (P16). At the termination of the experiment, the SIAIS361034-treated mice maintained a similar weight growth rate as control littermates. However, the reported Bcl-x_L_ PROTAC degrader DT2216 34, targeting Bcl-x_L_ to VHL E3 ligase, decelerated the rate of weight growth (Figure 7C). Moreover, the mice administrated with DT2216 and GDC-0449 exhibited noticeable and smaller body sizes when compared to vehicle mice and SIAIS361034-treated mice (Figure 7D). Micro-CT examination showed that no abnormalities in the skeleton were found in SIAIS361034-treated mice at 6 weeks of age (Figure 7E). The overall bone structure, especially the femur and tibia, of the administered mice was intact and normal. However, we observed an obvious shortening of the femur after treatment with DT2216 and GDC-0449 (Figure 7E). Micro-CT analyses of femurs indicated that in contrast to GDC-0449 and DT2216, SIAIS361034 exhibited no significant difference in the growth plate when compared with Vehicle (Figure 7F). Consistent with the Micro-CT analysis observations, histological investigations of cross sections of the femur showed that the columnar organization of chondrocytes in the growth plate was not destroyed in mice administered with SIAIS361034 when compared to those treated with GDC-0449 and DT2216 (Figure 7G). IHC assays showed that the expression levels of Gli1 were significantly suppressed in femurs after treatment with GDC-0449 or DT2216, while treatment with SIAIS361034 resulted in no alteration when compared with Vehicle. Moreover, levels of Bcl-x_L_ were reduced in femurs following treatment with DT2216. No change of Bcl-x_L_ was observed when treated with GDC-0449 and SIAIS361034 (Figure 7H). SIAIS361034 showed no side effects on the platelets and other indispensable blood cells at the end of the experiment (Figure S4A-C). Collectively, these results showed that compared with Smo inhibitors GDC-0449 and VHL-based PROTAC DT2216, treatment with SIAIS361034 had no influence on the bone growth of young mice.

## Discussion

Similar to other targeted cancer therapeutics, the drug resistance is a common phenomenon for Smo inhibitors. The primary and acquired resistance to current Smo inhibitors largely hampered their clinical efficacy, thus, raising the critical need to develop novel strategies with capacity of combating the resistance to Smo inhibitors. The mechanisms of primary and acquired resistance to Smo inhibitors include mutations of *Smo*, amplification of *Gli2*, and upregulation of noncanonical Gli signaling 4. In this context, targeting the signaling components downstream of Smo, especially the transcriptional factors Gli, represents a promising strategy for combating the resistance to Smo inhibitors 50, 59-62.

Wu et al. reported that BH3 mimetics may inhibit the Hh signaling pathway by disrupting the Bcl-2 family proteins/Sufu binding to suppress the Gli activity, thus overcoming the resistance of Smo inhibitors 31. In this study, we determined that the Bcl-x_L_ specific PROTAC, SIAIS361034, significantly and selectively inhibited the Hh pathway activity *in vitro* and *in vivo*. Mechanistic investigations revealed that SIAIS361034 degraded Bcl-x_L_ and disabled Bcl-x_L_/Sufu interaction, therefore, allowing the engagement of Sufu with Gli1. Specifically, SIAIS361034 possessed the ability to combat the resistance to current Smo inhibitors caused by *Smo* mutations or amplification of *Gli2*. Hence, this study indicates the possibility of using SIAIS361034 for the treatment of tumors driven by the Hh pathway, especially for those resistant to current Smo inhibitors.

In limb development, Hh pathway is required for normal skeletal development and plays a vital role in bone formation during endochondral ossification 25, 63, 64. It has been well documented that inhibition of the Hh pathway with Smo inhibitors may cause defects in bone growth and premature bone growth plate closure 27, 30, 65. Therefore, such on-target toxicity in bone growth limits the use of Hh inhibitors in pediatric patients. Here, we established that SIAIS361034 significantly suppressed Hh activity without causing defects in bone development because it targets Bcl-x_L_ to the CRBN E3 ligase that is minimally expressed in chondrocytes and osteoblasts. We also observed severe defects in bone growth and dramatic loss of body weight after treatment with DT2216, which could be due to the highly expressed VHL E3 ligase in chondrocytes and osteoblasts. Besides similar ability in degrading Bcl-x_L_ protein and sparing platelets in comparison with the previous reported Bcl-x_L_ PROTAC DT2216, our PROTAC SIAIS361034 simultaneously possesses capacity of sparing bone growth due to the distinct E3 ligase we used. These results raise concerns about the use of VHL-recruiting PROTACs in pediatric patients and suggest that CRBN-recruiting PROTACs are considerably less toxic to bone growth than VHL-recruiting PROTACs.

Among the pro-survival Bcl-2 proteins, Bcl-x_L_ is the most predominantly overexpressed protein in many leukemia cells and solid tumors 66, 67. Additionally, acquired resistance to anticancer therapies in human cancers has been identified to be positively correlated with Bcl-x_L_ expression 68, 69. These findings identified Bcl-x_L_ as a good target for the treatment of hematological malignancies, such as acute lymphoblastic leukemia and chronic lymphocytic leukemia. Pediatric leukemia is the most common malignancy affecting children, accounting for 30% of all pediatric cancers 70-72. However, targeting Bcl-x_L_ for the treatment of young children with hematological malignancies carries the risk of skeletal depression because of Hh inhibition. Therefore, our findings raise concerns about the potential toxicities in bone growth associated with cancer therapies targeting Bcl-x_L_ with VHL-based PROTACs in children.

## Supplementary Material

Supplementary figures and table.

Supplementary data 1.

Supplementary data 2.

## Abbreviations

AML: acute myeloid leukemia
BCC: basal cell carcinoma
Bcl-x_L_: B-cell lymphoma extra large
BH: Bcl-2 homology
CMC-Na: sodium carboxymethyl cellulose
CRBN: celeblon
D_max_: maximum degradation
GAPDH: glyceraldehyde-3-phosphate dehydrogenase
H&E: hematoxylin and eosin
HF: hair follicle
Hh: hedgehog
HTRF: homogeneous time-resolved fluorescence
IC_50_: half maximal inhibitory concentration
IHC: immunohistochemistry
IKZF1: ikaros family zinc finger proteins 1
IKZF3: ikaros family zinc finger proteins 3
IP: immunoprecipitation
MB: medulloblastoma
PFA: paraformaldehyde
PK: pharmacokinetic
PROTAC: proteolysis targeting chimera
Ptch1: patched 1
PVDF: polyvinylidene difluoride
RBC: red blood cells
RIPA: radioimmunoprecipitation
RT-qPCR: real-time quantitative PCR
SAR: structure-activity relationship
Smo: smoothened
Sufu: suppressor of fused
TGI: tumor growth inhibition
UPS: ubiquitin proteasomes system
VHL: von Hippel-Lindau
