# Supplementary material for: The PROTAC selectively degrading Bcl-xL represents a novel Hedgehog pathway inhibitor with capacity of combating resistance to Smoothened inhibitors while sparing bone growth
Source: Theranostics. 2022 Oct 24;12(17):7476–90. doi: 10.7150/thno.75421 (PMC9691367; doi:10.7150/thno.75421)
Supplement: Supplementary file 1 — Supplementary figures and table. [file thnov12p7476s1.pdf]

The PROTAC selectively degrading Bcl-x_L represents a novel Hedgehog pathway inhibitor with capacity of combating resistance to Smoothened inhibitors while sparing bone growth

Supplementary Figure 1

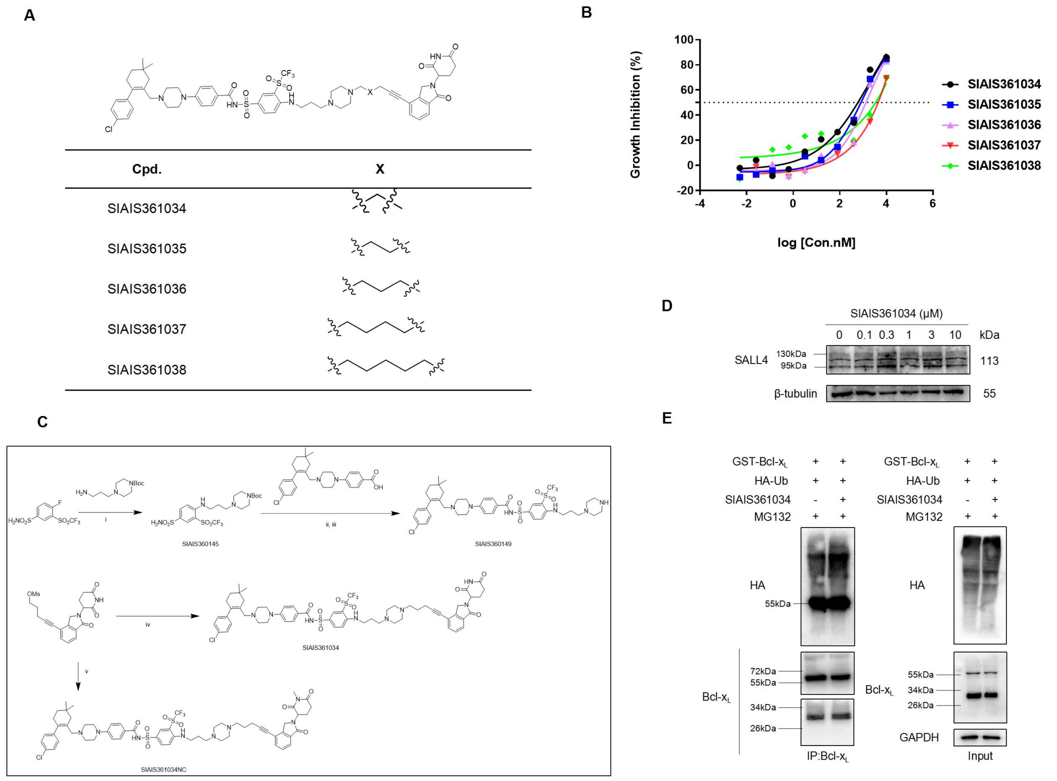

Figure S1. Optimization of linker length with linear aliphatic chains, synthetic schemes of SIAIS361034 and SIAIS361034NC. (A) Structures of Bcl-x_L degraders. **(B)** Cell viability of RS4;11 cells after treatment with increasing concentrations of Bcl-x_L degraders for 48 h. Data represent mean ± SD (n = 3). **(C)** Reagents and conditions: i) DIPEA, DCM, rt; ii) EDCI, DMAP, DCM, rt; iii) TFA, DCM; iv) SIAIS360149, K_2CO_3, NaI, DMF, 80 °C; v) NaH, MeI, DMF, 0 °C to rt; then SIAIS360149, K_2CO_3, NaI, DMF, 80 °C; DIPEA = *N*,*N*-diisopropylethylamine; DCM = dichloromethane; EDCI = 1-(3-dimethylaminopropyl)-3-ethylcarbodiimide

hydrochloride; DMAP = 4-dimethylaminopyridine; TFA = trifluoroacetic acid; DMF = *N*,*N*-dimethylformamide. **(D)** A representative western blot analysis of SALL4 in NIH-3T3 cells after being treated with SIAIS361034 as indicated for 24 h. **(E)** A representative western blot analysis of hemagglutinin (HA), Bcl-x_L, and GAPDH following GST-Bcl-x_L immunoprecipitation of protein extracts from 293T cells. Cells were cotransfected as indicated with GST-Bcl-x_L and HA-tagged ubiquitin (HA-Ub) and then were treated with or without SIAIS361034 (1 μM) and MG132 (10 μM) as indicated for 4 h.

Supplementary Figure 2

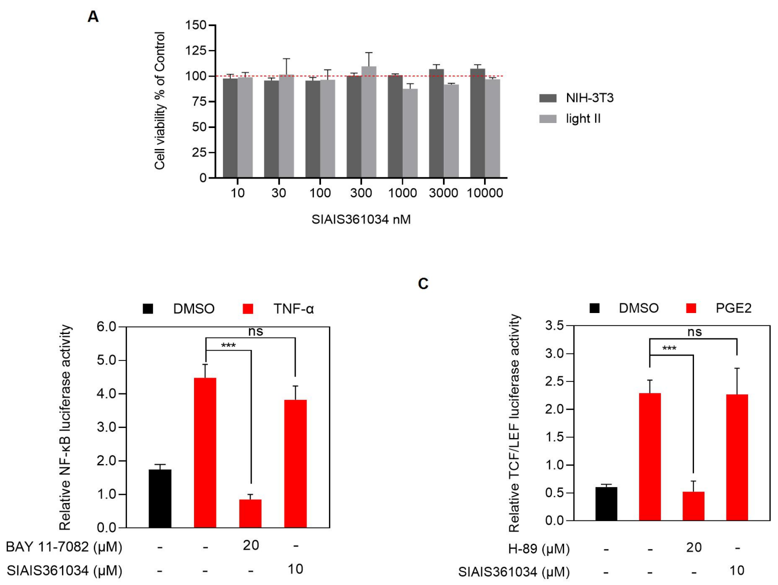

Figure S2. (A) Viability of NIH-3T3 cells and light II cells was determined by MTT cytotoxicity assays after incubation with various concentrations of SIAIS361034 as indicated for 72 h. Data represent mean ± SD (n = 3). **(B)** NF-κB luciferase analysis of the effect of SIAIS361034 on NF-κB signaling pathway in 293T cells. The 293T cells were transfected with NF-κB luciferase reporter and *Renilla*-TK plasmids and then exposed to TNF-α (10 ng/mL) with BAY 11-872 (20 μM) and SIAIS361034 (10 μM) for 6 h. Data represent mean ± SD (n = 3). **(C)** TCF/LEF luciferase analysis of the effect of SIAIS361034 on Wnt signaling pathway in LS174T cells. The LS174T cells were transfected with TCF/LEF luciferase reporter and *Renilla*-TK plasmids and then exposed to PGE2 (1 μM) with H-89 (20 μM) and SIAIS361034 (10 μM) for 24 h. Data represent mean ± SD (n = 3).

Supplementary Figure 3

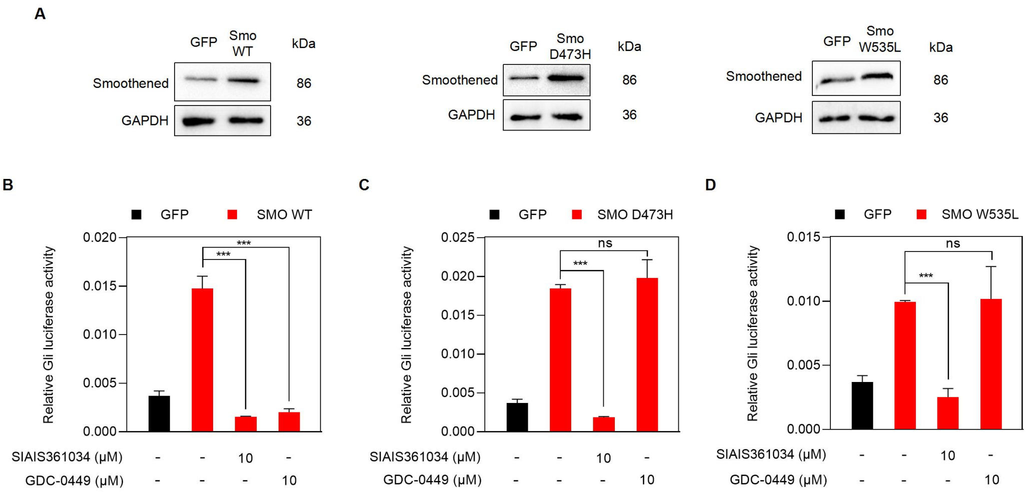

Figure S3. (A) A representative western blot analysis of Smo in light II cells after transfected with Smo-WT, Smo-D473H, Smo-W535L, and GFP plasmids. GAPDH was used as a protein loading control in western blot analysis. **(B-D)** The effect of SIAIS361034 (10 μM) and GDC-0449 (10 μM) on the Gli-luciferase activity initiated by ectopic expression of Smo-WT, Smo-D473H, and Smo-W535L plasmids, respectively. Data represent mean ± SD (n = 3).

Supplementary Figure 4

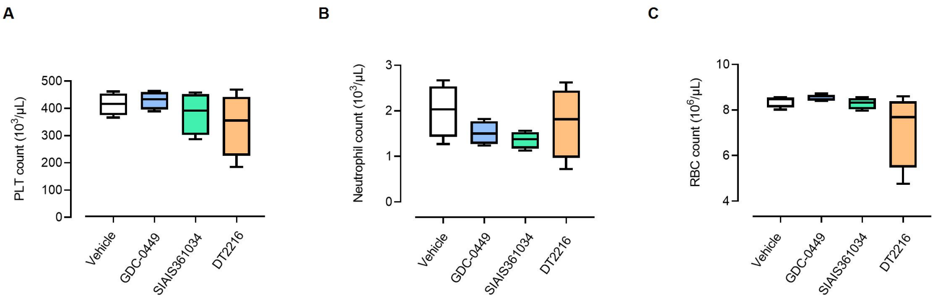

Figure S4. Numeration of platelets (**A**), neutrophils (**B**), and red blood cells (**C**) of 6-week-old mice after various treatments from P12 to P16. Data represent mean ± SD (n = 4).

Supplementary Table 1. Mouse PK parameters following IV/IP/PO Administration with SIAIS361034.

IV<br>Animal No.	Administration Route	Dose Level<br>mg/kg	$T_{1/2}$<br>h	T_{max}<br>h	C_{max}<br>ng/mL	$AUC_{(0-t)}$<br>h*ng/mL	$AUC_{(0-\infty)}$<br>h*ng/mL	$MRT_{(0-t)}$<br>h	$MRT_{(0-\infty)}$<br>h	C_0<br>ng/mL	V_{ss}<br>mL/kg	V_z<br>mL/kg	Cl<br>mL/h/kg
Mouse 1	IV	2.00	1.79	0.083	10300	3193	3205	0.22	0.26	34215	163	1609	624
Mouse 2	IV	2.00	6.38	0.083	17900	6203	6222	0.30	0.40	79513	130	2957	321
Mouse 3	IV	2.00	2.29	0.083	13200	4263	4305	0.31	0.42	43287	193	1535	465
n		3.00	3.00	3.00	3.00	3.00	3.00	3.00	3.00	3.00	3.00	3.00	3.00
Mean		2.00	3.49	0.083	13800	4553	4577	0.28	0.36	52339	162	2034	470
SD		0.000	2.52	0.00	3835	1526	1527	0.05	0.09	23967	32	801	151

IP<br>Animal No.	Administration Route	Dose Level<br>mg/kg	$T_{1/2}$<br>h	T_{max}<br>h	C_{max}<br>ng/mL	$AUC_{(0-t)}$<br>h*ng/mL	$AUC_{(0-\infty)}$<br>h*ng/mL	$MRT_{(0-t)}$<br>h	$MRT_{(0-\infty)}$<br>h	F<br>%
Mouse 1	IP	10	5.94	1.00	7630	15533	16333	3.84	5.25	71.4
Mouse 2	IP	10	6.44	0.50	9700	20871	22061	5.16	6.68	96.4
Mouse 3	IP	10	5.23	0.50	4920	15558	16141	4.24	5.22	70.5
n		3.00	3.00	3.00	3.00	3.00	3.00	3.00	3.00	3.00
Mean		10.0	5.87	0.67	7417	17321	18178	4.41	5.72	79.4
SD		0.00	0.61	0.29	2397	3075	3364	0.68	0.83	14.7

PO<br>Animal No.	Administration Route	Dose Level<br>mg/kg	$T_{1/2}$<br>h	T_{max}<br>h	C_{max}<br>ng/mL	$AUC_{(0-t)}$<br>h*ng/mL	$AUC_{(0-\infty)}$<br>h*ng/mL	$MRT_{(0-t)}$<br>h	$MRT_{(0-\infty)}$<br>h	F<br>%
Mouse 1	PO	10	8.98	1.00	50.9	265	305	6.29	10.30	1.33
Mouse 2	PO	10	NA	2.00	54.9	213	NA	2.88	NA	NA
Mouse 3	PO	10	2.39	1.00	49.1	101	111	2.01	2.82	0.484
n		3.00	3.00	3.00	3.00	3.00	3.00	3.00	3.00	3.00
Mean		10.0	5.68	1.33	51.6	193	208	3.73	6.56	NA
SD		0.00	4.66	0.58	2.97	83.7	137	2.27	5.28	0.60
