# Supplementary material for: The PROTAC selectively degrading Bcl-xL represents a novel Hedgehog pathway inhibitor with capacity of combating resistance to Smoothened inhibitors while sparing bone growth
Source: Theranostics. 2022 Oct 24;12(17):7476–90. doi: 10.7150/thno.75421 (PMC9691367; doi:10.7150/thno.75421)
Supplement: Supplementary file 2 — Supplementary data 1. [file thnov12p7476s2.pdf]

Supplementary data 1

Detailed synthetic procedures for SIAIS361034 and SIAIS361034NC

General methods.

Reagents and solvents were purchased from commercial sources without further purification, unless otherwise indicated. The final compounds were purified by prepared HPLC. The progress of reactions was monitored by thin-layer chromatography (TLC) and/or LC-MS. NMR spectra were obtained from an AVANCE III 500 MHz Bruker spectrometer (operating at 500 MHz for 1H NMR). Multiplicities of signals are described as follows: s --- singlet, br. s --- broad singlet, d --- doublet, t --- triplet, m --- multiple. High Resolution Mass spectra were recorded on AB Triple 4600 spectrometer with acetonitrile and water as solvents.

Preparation of *tert*-butyl 4-(3-((4-sulfamoyl-2-((trifluoromethyl)sulfonyl)phenyl)amino)propyl)piperazine-1-carboxylate (compound **SIAIS360145**). To a solution of 4-fluoro-3-((trifluoromethyl)sulfonyl)benzenesulfonamide (1.0 g, 3.25 mmol) in DCM (25 mL) were added tert-butyl 4-(3-aminopropyl)piperazine-1-carboxylate (0.87 g, 3.57 mmol) and DIPEA (0.85 mL, 4.87 mmol). The mixture solution was stirred at room temperature for 12 h and concentrated. The residue was purified by silica gel flash column chromatography (eluent: DCM – DMC/MeOH = 10/1). White solid (1.55 g, yield 90%). ^{1}H NMR (500 MHz, DMSO-d_6) δ 8.01 (d, J = 2.3 Hz, 1H), 7.95 (dd, J = 9.2, 2.4 Hz, 1H), 7.38 (s, 2H), 7.32 (t, J = 5.7 Hz, 1H), 7.22 (d, J = 9.3 Hz, 1H), 3.41 (q, J = 6.7 Hz, 2H), 3.32 (s, 4H), 2.36 – 2.32 (m, 2H), 2.29 (t, J = 5.1 Hz, 4H), 1.74-1.69 (m, 2H), 1.39 (s, 9H). MS (ESI) m/z: calcd. for $C_{19}H_{30}F_3N_4O_6S_2^+$ [M+H]$^+$, 531.2; found 531.4.

Preparation of 4-(4-((4'-chloro-4,4-dimethyl-3,4,5,6-tetrahydro-[1,1'-biphenyl]-2-yl)methyl)piperazin-1-yl)-*N*-((4-((3-(piperazin-1-yl)propyl)amino)-3-((trifluoromethyl)sulfonyl)phenyl)sulfonyl)benzamide (**SIAIS360149** TFA salt). To a solution of 4-(4-((4'-chloro-4,4-dimethyl-3,4,5,6-tetrahydro-[1,1'-biphenyl]-2-yl)methyl)piperazin-1-yl)benzoic acid (0.99 g, 2.26 mmol) in DCM (20 mL) were added EDCI (0.87 g, 4.52 mmol), SIAIS360145 (1.0 g, 1.88 mmol) and DMAP (0.28 g, 2.26 mmol). The reaction mixture was stirred at room temperature for 12 h and quenched with the saturated NH_4Cl aqueous solution (20 mL). The organic phase was separated and the aqueous phase was extracted with DCM (25 mL). The organic phases were combined and washed with brine. Concentrated and the residue was purified by silica gel flash column chromatography (eluent: DCM – DMC/MeOH = 10/1) to afford a white solid. The white solid was dissolved into DCM (10 mL), TFA (5 mL) was added and the reaction mixture was stirred at room temperature for 2 h. The solvent and TFA were removed under reduced pressure and lyophilized. White solid (1.39 g, yield 78%). ^{1}H NMR (500 MHz, DMSO-d_6) δ 9.28 (br. s, 1H), 8.24 (d, J = 2.3 Hz, 1H), 8.07 (dd, J = 9.2, 2.4 Hz, 1H), 7.76 (d, J = 8.9 Hz, 2H), 7.52 (t, J = 6.0 Hz, 1H), 7.42 (d, J = 8.4 Hz, 2H), 7.26 (d, J = 9.5 Hz, 1H), 7.17 (d, J = 8.4 Hz, 2H), 6.96 (d, J = 9.2 Hz, 2H), 3.93 (s, 2H), 3.60 (s, 2H), 3.47 (d, J = 6.5 Hz, 3H), 3.45 – 3.11 (m, 13H), 3.02 (s, 3H), 2.81 (s, 2H), 2.29 (s, 2H), 2.05 (s, 2H), 1.90-1.87 (m, 2H), 1.48 (t, J = 6.4 Hz, 2H), 1.00 (s, 6H). MS (ESI) m/z: calcd. for $C_{40}H_{51}ClF_3N_6O_5S_2^+$ [M+H]$^+$, 851.2997; found 851.2974.

Preparation of 4-(4-((4'-chloro-4,4-dimethyl-3,4,5,6-tetrahydro-[1,1'-biphenyl]-2-yl)methyl)piperazin-1-yl)-*N*-((4-((3-

(4-(5-(2-(2,6-dioxopiperidin-3-yl)-1-oxoisoindolin-4-yl)pent-4-yn-1-yl)piperazin-1-yl)propyl)amino)-3-((trifluoromethyl)sulfonyl)phenyl)sulfonyl)benzamide (**SIAIS361034**). To a solution of SIAIS 360149 (25 mg, 0.021 mmol) in DMF (2 mL) were added K_2CO_3 (8.7 mg, 0.063 mmol), NaI (4.7 mg, 0.032 mmol) and 5-(2-(2,6-dioxopiperidin-3-yl)-1-oxoisoindolin-4-yl)pent-4-yn-1-yl methanesulfonate (prepared according to patent WO 2018052949) (10.2 mg, 0.025mmol). The reaction mixture was stirred at 80 °C for 12 h and purified by prepared HPLC (acetonitrile/H_2O (containing 0.05% HCl)). White solid (6.4 mg, 24%). ^{1}H NMR (500 MHz, CD_3OD) δ 8.38 (d, *J* = 2.3 Hz, 1H), 8.18 (dd, *J* = 9.3, 2.3 Hz, 1H), 7.74 (t, *J* = 8.4 Hz, 3H), 7.63 (dd, *J* = 7.7, 1.0 Hz, 1H), 7.50 (t, *J* = 7.6 Hz, 1H), 7.39 (d, *J* = 8.4 Hz, 2H), 7.21 – 7.13 (m, 3H), 6.96 (d, *J* = 9.1 Hz, 2H), 5.16 (dd, *J* = 13.3, 5.2 Hz, 1H), 4.57 (d, *J* = 17.6 Hz, 1H), 4.50 (d, *J* = 17.5 Hz, 1H), 3.88 (d, *J* = 13.9 Hz, 2H), 3.71 (s, 2H), 3.68 – 3.36 (m, 10H), 3.29 – 3.14 (m, 6H), 3.01 (s, 2H), 2.95 – 2.81 (m, 3H), 2.79 - 2.72(m, 1H), 2.66 (t, *J* = 6.8 Hz, 2H), 2.56 (qd, *J* = 13.2, 4.6 Hz, 1H), 2.41 (t, *J* = 6.7 Hz, 2H), 2.20 - 2.13 (m, 1H), 2.13 – 2.07 (m, 4H), 2.06 – 1.97 (m, 2H), 1.57 (t, *J* = 6.4 Hz, 2H), 1.08 (s, 6H). ^{13}C NMR (126 MHz, DMSO-*d*$_6$) δ 173.02, 171.10, 167.74, 164.70, 152.79, 151.93, 144.12, 141.36, 140.30, 137.03, 134.83, 134.16, 132.03, 131.84, 130.34, 129.98, 128.74, 128.68, 125.92, 122.91, 122.36, 120.48, 119.89 (q, *J* = 327.6 Hz), 118.53, 114.58, 113.65, 106.62, 94.48, 77.35, 58.30, 54.83, 53.27, 51.80, 50.41, 48.06, 47.31, 43.30, 41.30, 40.17, 40.11, 39.95, 39.78, 34.65, 31.27, 30.94, 28.84, 27.80, 22.49, 16.36. HRMS (ESI) m/z: calcd. for $C_{58}H_{67}ClF_3N_8O_8S_2^+$ [M+H]$^+$, 1159.4158; found 1159.4188.

Preparation of 4-(4-((4'-chloro-4,4-dimethyl-3,4,5,6-tetrahydro-[1,1'-biphenyl]-2-yl)methyl)piperazin-1-yl)-*N*-((4-((3-(4-(5-(2-(1-methyl-2,6-dioxopiperidin-3-yl)-1-oxoisoindolin-4-yl)pent-4-yn-1-yl)piperazin-1-yl)propyl)amino)-3-((trifluoromethyl)sulfonyl)phenyl)sulfonyl)benzamide (**SIAIS361034NC**). To a solution of 5-(2-(2,6-dioxopiperidin-3-yl)-1-oxoisoindolin-4-yl)pent-4-yn-1-yl methanesulfonate (25 mg, 0.062 mmol) in DMF (2 mL) in 0°C was added NaH ((60%, dispersion in mineral oil) (10 mg, 0.24 mmol). The mixture was stirred for 15 min before MeI (5.8 μL, 0.093 mmol) was added. The reaction mixture was naturally warmed to room temperature and further stirred for 2 h until the reaction was completed (monitored by LC-MS). The reaction mixture was quenched with NH_4Cl aqueous solution (15 mL) and extracted with EA (20 mL). The organic phase was washed with water (20 mL) for 3 times and brine for 1 time, dried over anhydrous Na_2SO_4 and concentrated. The residue was purified by silica gel flash column chromatography (eluent: DCM – DMC/MeOH = 10/1) to afford the intermediate. To a solution of SIAIS 360149 (25 mg, 0.021 mmol) in DMF (2 mL) were added K_2CO_3 (8.7 mg, 0.063 mmol), NaI (4.7 mg, 0.032 mmol) and the above intermediate (10.5 mg, 0.025mmol). The reaction mixture was stirred at 80°C for 12 h and purified by prepared HPLC (eluent: acetonitrile/H_2O (containing 0.05% HCl)). White solid (10 mg, 37%). ^{1}H NMR (500 MHz, DMSO-*d*$_6$) δ 12.16 (br. s, 1H), 10.34 (br. s, 1H), 8.23 (d, *J* = 2.4 Hz, 1H), 8.06 (dd, *J* = 9.2, 2.4 Hz, 1H), 7.74 (dd, *J* = 10.4, 8.1 Hz, 3H), 7.67 (d, *J* = 7.6 Hz, 1H), 7.54 (t, *J* = 7.6 Hz, 2H), 7.41 (d, *J* = 8.4 Hz, 2H), 7.30 (d, *J* = 9.2 Hz, 1H), 7.17 (d, *J* = 8.4 Hz, 2H), 6.95 (d, *J* = 9.2 Hz, 2H), 5.22 (dd, *J* = 13.5, 5.1 Hz, 1H), 4.51 (d, *J* = 17.7 Hz, 1H), 4.36 (d, *J* = 17.7 Hz, 1H), 3.88 (d, *J* = 13.7 Hz, 2H), 3.83 – 3.44 (m, 10H), 3.40 – 3.00 (m, 12H), 2.99 (s, 3H), 2.80 - 2.70 (m, 3H), 2.60 (t, *J* = 6.9 Hz, 2H), 2.27 (t, *J* = 6.7 Hz, 2H), 2.16 (s, 2H), 2.06 – 1.86 (m, 5H), 1.46 (t, *J* = 6.5 Hz, 2H), 1.00 (s, 6H). ^{13}C NMR (126 MHz, DMSO-*d*$_6$) δ 171.98, 170.64, 167.79, 164.73, 152.76, 151.96, 144.06, 141.29, 140.29, 137.07, 134.80, 134.23, 132.02, 131.84, 130.30, 129.95, 128.72, 128.68, 125.94, 122.93, 122.38, 120.56, 119.89 (q, *J* = 327.5 Hz), 118.56, 114.46, 113.64, 106.54, 94.62, 77.25, 58.38, 54.82, 53.24, 52.25, 50.45, 48.54, 47.23, 43.40, 41.20, 40.37, 40.11, 39.94, 39.78, 34.64, 31.42, 30.92, 28.82, 27.79, 26.68, 21.74, 16.40. HRMS (ESI) m/z: calcd. for

$C_{59}H_{69}ClF_3N_8O_8S_2^+$ [M+H]$^+$, 1173.4315; found 1173.4312.

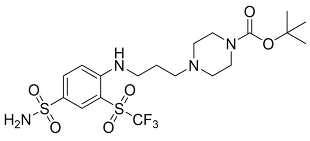

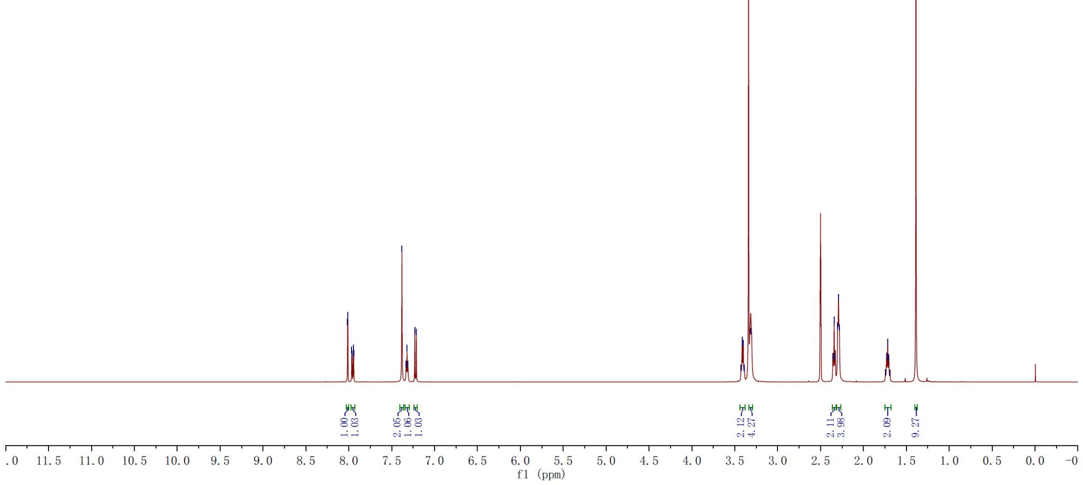

SIAIS360145

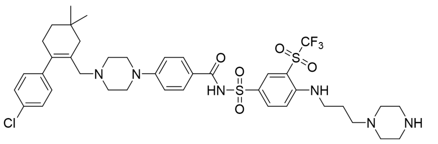

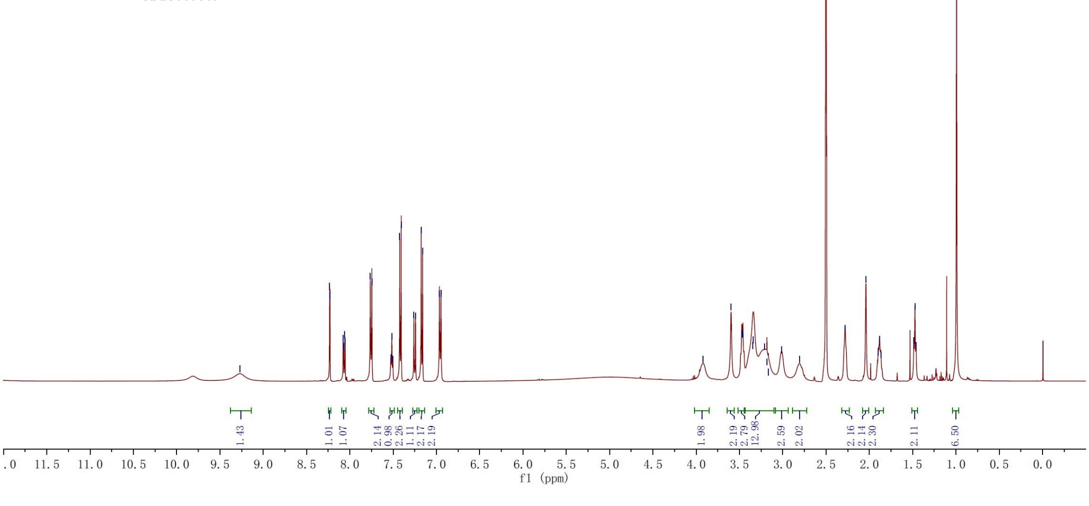

SIAIS360149

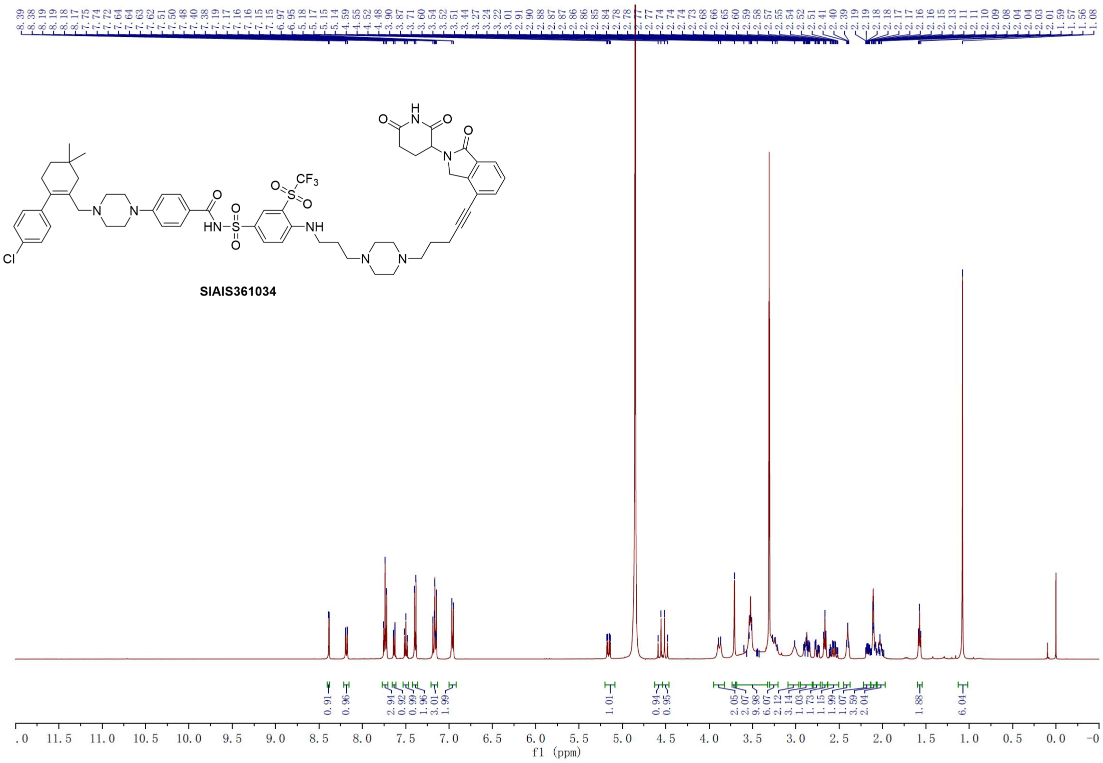
SIAIS361034
f1 (ppm)

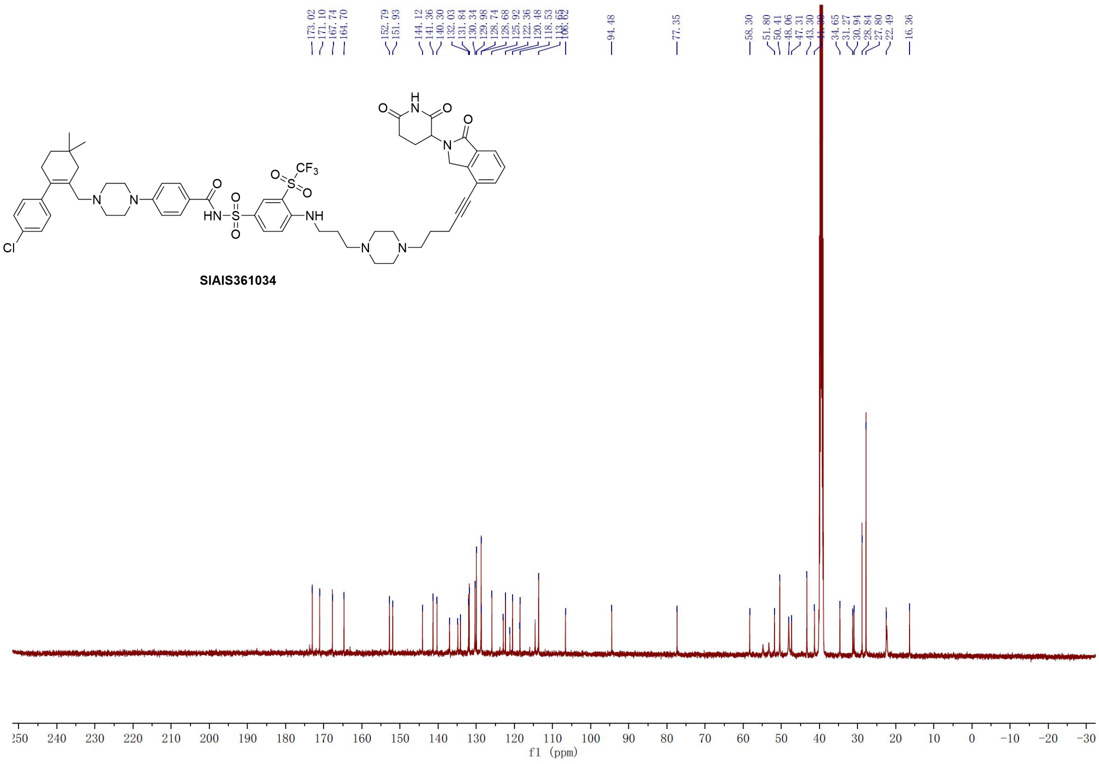
SIAIS361034
f1 (ppm)

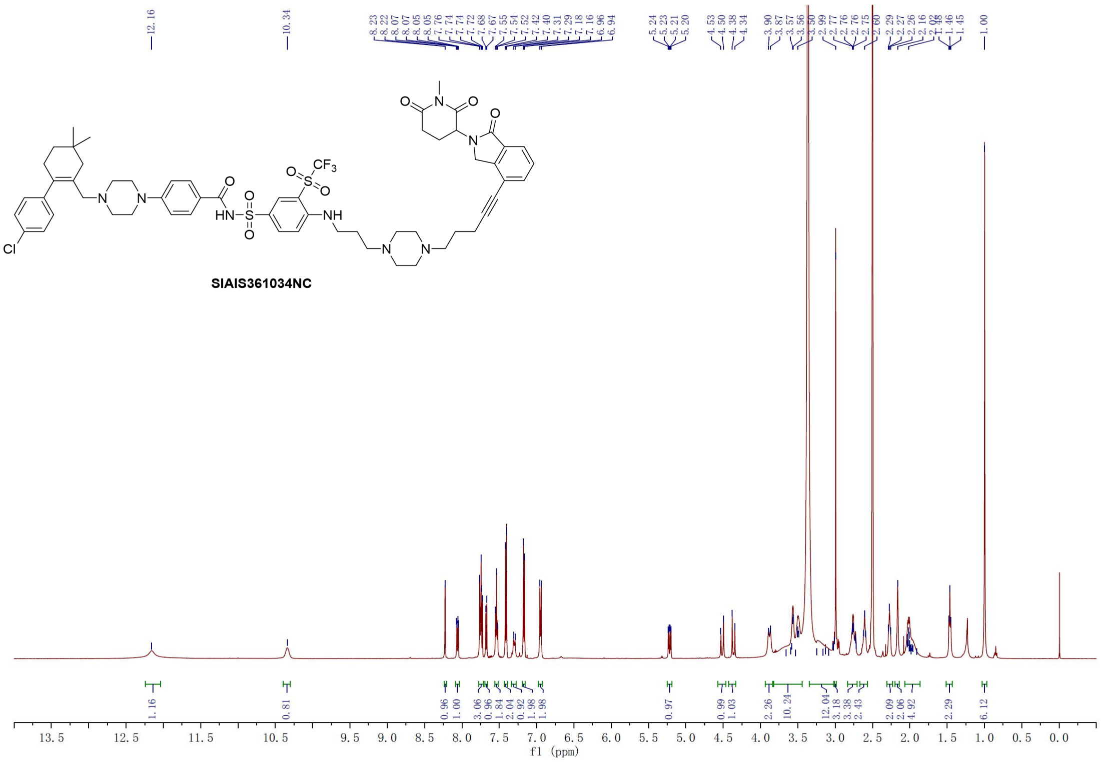
SIAIS361034NC
f1 (ppm)

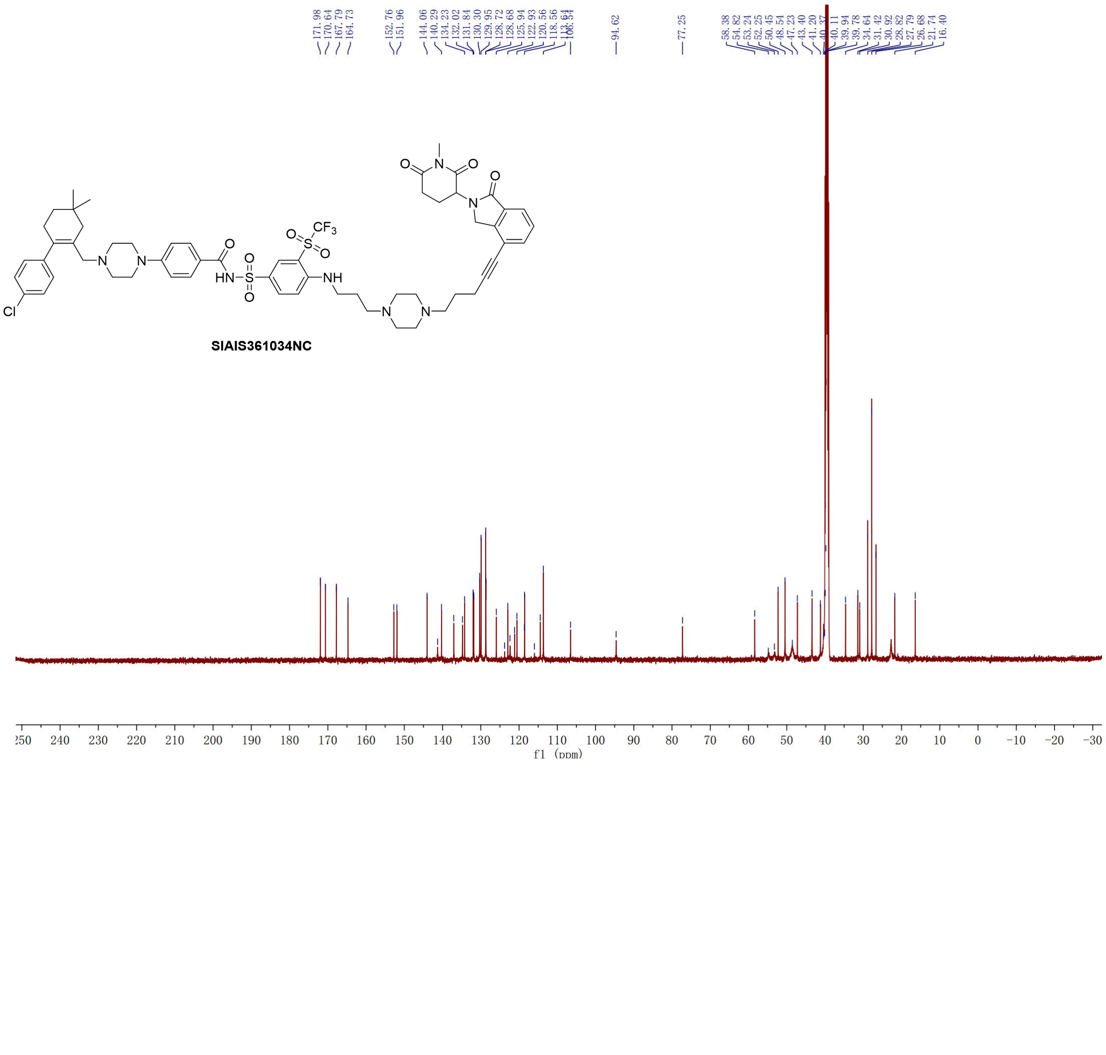
SIAIS361034NC
f1 (ppm)
