# Supplementary material for: The PROTAC selectively degrading Bcl-xL represents a novel Hedgehog pathway inhibitor with capacity of combating resistance to Smoothened inhibitors while sparing bone growth
Source: Theranostics. 2022 Oct 24;12(17):7476–90. doi: 10.7150/thno.75421 (PMC9691367; doi:10.7150/thno.75421)
Supplement: Supplementary file 3 — Supplementary data 2. [file thnov12p7476s3.pdf]

Supplementary data 2
Full sequence information of Smo mutants plasmids are provided below.

Smo D473H:
CAGAGCTCTCTGGCTAACTAGAGAACCCACTGCTTACTGGCTTATCGAAATTAATACGA
CTCACTATAGGGAGACCCAAGCTGGCTAGCGTTTAAACTTAAGCTTGGTACCGAGCTC
GGATCCGCCACCATGGCCGCTGCCCGCCCAGCGCGGGGGCCGGAGCTCCCGCTCCTG
GGGCTGCTGCTGCTGCTGCTGCTGGGGGACCCGGGCCGGGGGGCGGCCTCGAGCGGG
AACGCGACCGGGCCTGGGCCTCGGAGCGCGGGCGGGAGCGCGAGGAGGAGCGCGGC
GGTGACTGGCCCTCCGCCGCCGCTGAGCCACTGCGGCCGGGCTGCCCCCTGCGAGCC
GCTGCGCTACAACGTGTGCCTGGGCTCGGTGCTGCCCTACGGGGCCACCTCCACACTG
CTGGCCGGAGACTCGGACTCCCAGGAGGAAGCGCACGGCAAGCTCGTGCTCTGGTCG
GGCCTCCGGAATGCCCCCCGCTGCTGGGCAGTGATCCAGCCCCTGCTGTGTGCCGTAT
ACATGCCCAAGTGTGAGAATGACCGGGTGGAGCTGCCCAGCCGTACCCTCTGCCAGGC
CACCCGAGGCCCCTGTGCCATCGTGGAGAGGGAGCGGGGCTGGCCTGACTTCCTGCG
CTGCACTCCTGACCGCTTCCCTGAAGGCTGCACGAATGAGGTGCAGAACATCAAGTTC
AACAGTTCAGGCCAGTGCGAAGTGCCCTTGGTTCGGACAGACAACCCCAAGAGCTGG
TACGAGGACGTGGAGGGCTGCGGCATCCAGTGCCAGAACCCGCTCTTCACAGAGGCT
GAGCACCAGGACATGCACAGCTACATCGCGGCCTTCGGGGCCGTCACGGGCCTCTGCA
CGCTCTTCACCCTGGCCACATTCGTGGCTGACTGGCGGAACTCGAATCGCTACCCTGCT
GTTATTCTCTTCTACGTCAATGCGTGCTTCTTTGTGGGCAGCATTGGCTGGCTGGCCCA
GTTCATGGATGGTGCCCGCCGAGAGATCGTCTGCCGTGCAGATGGCACCATGAGGCTT
GGGGAGCCCACCTCCAATGAGACTCTGTCCTGCGTCATCATCTTTGTCATCGTGTACTA
CGCCCTGATGGCTGGTGTGGTTTGGTTTGTGGTCCTCACCTATGCCTGGCACACTTCCT
TCAAAGCCCTGGGCACCACCTACCAGCCTCTCTCGGGCAAGACCTCCTACTTCCACCT
GCTCACCTGGTCACTCCCCTTTGTCCTCACTGTGGCAATCCTTGCTGTGGCGCAGGTGG
ATGGGGACTCTGTGAGTGGCATTTGTTTTGTGGGCTACAAGAACTACCGATACCGTGCG
GGCTTCGTGCTGGCCCCAATCGGCCTGGTGCTCATCGTGGGAGGCTACTTCCTCATCCG
AGGAGTCATGACTCTGTTCTCCATCAAGAGCAACCACCCCGGGCTGCTGAGTGAGAA
GGCTGCCAGCAAGATCAACGAGACCATGCTGCGCCTGGGCATTTTTGGCTTCCTGGCC
TTTGGCTTTGTGCTCATTACCTTCAGCTGCCACTTCTACCACTTCTTCAACCAGGCTGA
GTGGGAGCGCAGCTTCCGGGACTATGTGCTATGTCAGGCCAATGTGACCATCGGGCTG
CCCACCAAGCAGCCCATCCCTGACTGTGAGATCAAGAATCGCCCGAGCCTTCTGGTGG
AGAAGATCAACCTGTTTGCCATGTTTGGAACTGGCATCGCCATGAGCACCTGGGTCTG
GACCAAGGCCACGCTGCTCATCTGGAGGCGTACCTGGTGCAGGTTGACTGGGCAGAG
TGACGATGAGCCAAAGCGGATCAAGAAGAGCAAGATGATTGCCAAGGCCTTCTCTAA
GCGGCACGAGCTCCTGCAGAACCCAGGCCAGGAGCTGTCCTTCAGCATGCACACTGT
GTCCCACGACGGGCCCGTGGCGGGCTTGGCCTTTGACCTCAATGAGCCCTCAGCTGAT
GTCTCCTCTGCCTGGGCCCAGCATGTCACCAAGATGGTGGCTCGGAGAGGAGCCATAC
TGCCCCAGGATATTTCTGTCACCCCTGTGGCAACTCCAGTGCCCCCAGAGGAACAAGC
CAACCTGTGGCTGGTTGAGGCAGAGATCTCCCCAGAGCTGCAGAAGCGCCTGGGCCG
GAAGAAGAAGAGGAGGAAGAGGAAGAAGGAGGTGTGCCCGCTGGCGCCGCCCCCTG
AGCTTCACCCCCCCTGCCCCTGCCCCCAGTACCATTCCTCGACTGCCTCAGCTGCCCCGG
CAGAAATGCCTGGTGGCTGCAGGTGCCTGGGGAGCTGGGGACTCTTGCCGACAGGGA

GCGTGGACCCTGGTCTCCAACCCATTCTGCCCAGAGCCCAGTCCCCCTCAGGATCCAT
TTCTGCCCAGTGCACCGGCCCCCGTGGCATGGGCTCATGGCCGCCGACAGGGCCTGGG
GCCTATTCACTCCCGCACCAACCTGATGGACACAGAACTCATGGATGCAGACTCGGAC
TTCTGACTCGAGTCT

Smo W535L:
TGCGATGGTGCAATAGCAGAGCTCGTTTAGTGACCGTCAGAATTTTGTAATACGACTCA
CTATAGGGCGGCCGGGAATTCGTCGACTGGATCCGGTACCGAGGAGATCTGCCGCCGC
GATCGCCATGGCCGCTGCCCGCCCAGCGCGGGGGCCGGAGCTCCCGCTCCTGGGGCT
GCTGCTGCTGCTGCTGCTGGGGGACCCGGGCCGGGGGGCGGCCTCGAGCGGGAACGC
GACCGGGCCTGGGCCTCGGAGCGCGGGCGGGAGCGCGAGGAGGAGCGCGGCGGTGA
CTGGCCCTCCGCCGCCGCTGAGCCACTGCGGCCGGGCTGCCCCCTGCGAGCCGCTGC
GCTACAACGTGTGCCTGGGCTCGGTGCTGCCCTACGGGGCCACCTCCACACTGCTGGC
CGGAGACTCGGACTCCCAGGAGGAAGCGCACGGCAAGCTCGTGCTCTGGTCGGGCCT
CCGGAATGCCCCCCGCTGCTGGGCAGTGATCCAGCCCCTGCTGTGTGCCGTATACATGC
CCAAGTGTGAGAATGACCGGGTGGAGCTGCCCAGCCGTACCCTCTGCCAGGCCACCC
GAGGCCCCTGTGCCATCGTGGAGAGGGAGCGGGGCTGGCCTGACTTCCTGCGCTGCA
CTCCTGACCGCTTCCCTGAAGGCTGCACGAATGAGGTGCAGAACATCAAGTTCAACAG
TTCAGGCCAGTGCGAAGTGCCCTTGGTTCGGACAGACAACCCCAAGAGCTGGTACGA
GGACGTGGAGGGCTGCGGCATCCAGTGCCAGAACCCGCTCTTCACAGAGGCTGAGCA
CCAGGACATGCACAGCTACATCGCGGCCTTCGGGGCCGTCACGGGCCTCTGCACGCTC
TTCACCCTGGCCACATTCGTGGCTGACTGGCGGAACTCGAATCGCTACCCTGCTGTTAT
TCTCTTCTACGTCAATGCGTGCTTCTTTGTGGGCAGCATTGGCTGGCTGGCCCAGTTCA
TGGATGGTGCCCGCCGAGAGATCGTCTGCCGTGCAGATGGCACCATGAGGCTTGGGGA
GCCCACCTCCAATGAGACTCTGTCCTGCGTCATCATCTTTGTCATCGTGTACTACGCCCT
GATGGCTGGTGTGGTTTGGTTTGTGGTCCTCACCTATGCCTGGCACACTTCCTTCAAAG
CCCTGGGCACCACCTACCAGCCTCTCTCGGGCAAGACCTCCTACTTCCACCTGCTCAC
CTGGTCACTCCCCTTTGTCCTCACTGTGGCAATCCTTGCTGTGGCGCAGGTGGATGGG
GACTCTGTGAGTGGCATTTGTTTTGTGGGCTACAAGAACTACCGATACCGTGCGGGCTT
CGTGCTGGCCCCAATCGGCCTGGTGCTCATCGTGGGAGGCTACTTCCTCATCCGAGGA
GTCATGACTCTGTTCTCCATCAAGAGCAACCACCCCGGGCTGCTGAGTGAGAAGGCTG
CCAGCAAGATCAACGAGACCATGCTGCGCCTGGGCATTTTTGGCTTCCTGGCCTTTGG
CTTTGTGCTCATTACCTTCAGCTGCCACTTCTACGACTTCTTCAACCAGGCTGAGTGGG
AGCGCAGCTTCCGGGACTATGTGCTATGTCAGGCCAATGTGACCATCGGGCTGCCCAC
CAAGCAGCCCATCCCTGACTGTGAGATCAAGAATCGCCCGAGCCTTCTGGTGGAGAAG
ATCAACCTGTTTGCCATGTTTGGAACTGGCATCGCCATGAGCACCCTCGTCTGGACCAA
GGCCACGCTGCTCATCTGGAGGCGTACCTGGTGCAGGTTGACTGGGCAGAGTGACGAT
GAGCCAAAGCGGATCAAGAAGAGCAAGATGATTGCCAAGGCCTTCTCTAAGCGGCAC
GAGCTCCTGCAGAACCCAGGCCAGGAGCTGTCCTTCAGCATGCACACTGTGTCCCACG
ACGGGCCCGTGGCGGGCTTGGCCTTTGACCTCAATGAGCCCTCAGCTGATGTCTCCTC
TGCCTGGGCCCAGCATGTCACCAAGATGGTGGCTCGGAGAGGAGCCATACTGCCCCAG
GATATTTCTGTCACCCCTGTGGCAACTCCAGTGCCCCCAGAGGAACAAGCCAACCTGT
GGCTGGTTGAGGCAGAGATCTCCCCAGAGCTGCAGAAGCGCCTGGGCCGGAAGAAGA
AGAGGAGGAAGAGGAAGAAGGAGGTGTGCCCGCTGGCGCCGCCCCCTGAGCTTCAC

CCCCCTGCCCCTGCCCCCAGTACCATTCCTCGACTGCCTCAGCTGCCCCGGCAGAAAT
GCCTGGTGGCTGCAGGTGCCTGGGGAGCTGGGGACTCTTGCCGACAGGGAGCGTGGA
CCCTGGTCTCCAACCCATTCTGCCCAGAGCCCAGTCCCCCTCAGGATCCATTTCTGCCC
AGTGCACCGGCCCCCGTGGCATGGGCTCATGGCCGCCGACAGGGCCTGGGGCCTATTC
ACTCCCGCACCAACCTGATGGACACAGAACTCATGGATGCAGACTCGGACTTCACGCG
TACGCGGCCGCTCGAGCAGAAACTCATCTCAGAAGAGGATCTGGCAGCAAATGATATC
CTGGATTACAAGGATGACGACGATAAGGTTTAAACGGCCGGCCGCGGTCATAGCTGTT
TCCTGAACAGATCCCGGGTGGCATCCCTGTGACCCCTCCCCAGTGCCTCTCCTGGCCCT
GGAAGTTGCCACTCCAGTGCCCACCAGCCTGTCCTAATAAATAGTCAACGT

Smo H231R:
CTAGAGAACCCACTGCTTACTGGCTTATCGAAATTAATACGACTCACTATAGGGAGACC
CAAGCTGGCTAGCGTTTAAACTTAAGCTTGGTACCGAGCTCGGATCCGCCACCATGGC
CGCTGCCCGCCCAGCGCGGGGGCCGGAGCTCCCGCTCCTGGGGCTGCTGCTGCTGCT
GCTGCTGGGGGACCCGGGCCGGGGGGCGGCCTCGAGCGGGAACGCGACCGGGCCTG
GGCCTCGGAGCGCGGGCGGGAGCGCGAGGAGGAGCGCGGCGGTGACTGGCCCTCCG
CCGCCGCTGAGCCACTGCGGCCGGGCTGCCCCCTGCGAGCCGCTGCGCTACAACGTGT
GCCTGGGCTCGGTGCTGCCCTACGGGGCCACCTCCACACTGCTGGCCGGAGACTCGG
ACTCCCAGGAGGAAGCGCACGGCAAGCTCGTGCTCTGGTCGGGCCTCCGGAATGCCC
CCCGCTGCTGGGCAGTGATCCAGCCCCTGCTGTGTGCCGTATACATGCCCAAGTGTGA
GAATGACCGGGTGGAGCTGCCCAGCCGTACCCTCTGCCAGGCCACCCGAGGCCCCTGT
GCCATCGTGGAGAGGGAGCGGGGCTGGCCTGACTTCCTGCGCTGCACTCCTGACCGCT
TCCCTGAAGGCTGCACGAATGAGGTGCAGAACATCAAGTTCAACAGTTCAGGCCAGT
GCGAAGTGCCCTTGGTTCGGACAGACAACCCCAAGAGCTGGTACGAGGACGTGGAGG
GCTGCGGCATCCAGTGCCAGAACCCGCTCTTCACAGAGGCTGAGCACCAGGACATGC
GCAGCTACATCGCGGCCTTCGGGGCCGTCACGGGCCTCTGCACGCTCTTCACCCTGGC
CACATTCGTGGCTGACTGGCGGAACTCGAATCGCTACCCTGCTGTTATTCTCTTCTACG
TCAATGCGTGCTTCTTTGTGGGCAGCATTGGCTGGCTGGCCCAGTTCATGGATGGTGCC
CGCCGAGAGATCGTCTGCCGTGCAGATGGCACCATGAGGCTTGGGGAGCCCACCTCCA
ATGAGACTCTGTCCTGCGTCATCATCTTTGTCATCGTGTACTACGCCCTGATGGCTGGTG
TGGTTTGGTTTGTGGTCCTCACCTATGCCTGGCACACTTCCTTCAAAGCCCTGGGCACC
ACCTACCAGCCTCTCTCGGGCAAGACCTCCTACTTCCACCTGCTCACCTGGTCACTCCC
CTTTGTCCTCACTGTGGCAATCCTTGCTGTGGCGCAGGTGGATGGGGACTCTGTGAGT
GGCATTTGTTTTGTGGGCTACAAGAACTACCGATACCGTGCGGGCTTCGTGCTGGCCCC
AATCGGCCTGGTGCTCATCGTGGGAGGCTACTTCCTCATCCGAGGAGTCATGACTCTGT
TCTCCATCAAGAGCAACCACCCCGGGCTGCTGAGTGAGAAGGCTGCCAGCAAGATCA
ACGAGACCATGCTGCGCCTGGGCATTTTTGGCTTCCTGGCCTTTGGCTTTGTGCTCATT
ACCTTCAGCTGCCACTTCTACGACTTCTTCAACCAGGCTGAGTGGGAGCGCAGCTTCC
GGGACTATGTGCTATGTCAGGCCAATGTGACCATCGGGCTGCCCACCAAGCAGCCCAT
CCCTGACTGTGAGATCAAGAATCGCCCGAGCCTTCTGGTGGAGAAGATCAACCTGTTT
GCCATGTTTGGAACTGGCATCGCCATGAGCACCTGGGTCTGGACCAAGGCCACGCTGC
TCATCTGGAGGCGTACCTGGTGCAGGTTGACTGGGCAGAGTGACGATGAGCCAAAGC
GGATCAAGAAGAGCAAGATGATTGCCAAGGCCTTCTCTAAGCGGCACGAGCTCCTGCA
GAACCCAGGCCAGGAGCTGTCCTTCAGCATGCACACTGTGTCCCACGACGGGCCCGT

GGCGGGCTTGGCCTTTGACCTCAATGAGCCCTCAGCTGATGTCTCCTCTGCCTGGGCC
CAGCATGTCACCAAGATGGTGGCTCGGAGAGGAGCCATACTGCCCCAGGATATTTCTG
TCACCCCTGTGGCAACTCCAGTGCCCCCAGAGGAACAAGCCAACCTGTGGCTGGTTG
AGGCAGAGATCTCCCCAGAGCTGCAGAAGCGCCTGGGCCGGAAGAAGAAGAGGAGG
AAGAGGAAGAAGGAGGTGTGCCCGCTGGCGCCGCCCCCTGAGCTTCACCCCCCTGCC
CCTGCCCCCAGTACCATTCCTCGACTGCCTCAGCTGCCCCGGCAGAAATGCCTGGTGG
CTGCAGGTGCCTGGGGAGCTGGGGACTCTTGCCGACAGGGAGCGTGGACCCTGGTCT
CCAACCCATTCTGCCCAGAGCCCAGTCCCCCTCAGGATCCATTTCTGCCCAGTGCACC
GGCCCCCGTGGCATGGGCTCATGGCCGCCGACAGGGCCTGGGGCCTATTCACTCCCGC
ACCAACCTGATGGACACAGAACTCATGGATGCAGACTCGGACTTCGAACAAAAACTC
ATCTCAGAAGAGGATCTGGACTACAAGGATGACGATGACAAGGATTACAAAGACGAC
GATGATAAGGACTATAAGGATGATGACGACAAATAATCTAGAGGGCCCGTTTAAACCCG
CTGATCAGCCTCGACTGTGCCTTCTAGTTGCCAGCCATCTGTTGTT

Smo W281C:
CTAGAGAACCCACTGCTTACTGGCTTATCGAAATTAATACGACTCACTATAGGGAGACC
CAAGCTGGCTAGCGTTTAAACTTAAGCTTGGTACCGAGCTCGGATCCGCCACCATGGC
CGCTGCCCGCCCAGCGCGGGGGCCGGAGCTCCCGCTCCTGGGGCTGCTGCTGCTGCT
GCTGCTGGGGGACCCGGGCCGGGGGGCGGCCTCGAGCGGGAACGCGACCGGGCCTG
GGCCTCGGAGCGCGGGCGGGAGCGCGAGGAGGAGCGCGGCGGTGACTGGCCCTCCG
CCGCCGCTGAGCCACTGCGGCCGGGCTGCCCCCTGCGAGCCGCTGCGCTACAACGTGT
GCCTGGGCTCGGTGCTGCCCTACGGGGCCACCTCCACACTGCTGGCCGGAGACTCGG
ACTCCCAGGAGGAAGCGCACGGCAAGCTCGTGCTCTGGTCGGGCCTCCGGAATGCCC
CCCGCTGCTGGGCAGTGATCCAGCCCCTGCTGTGTGCCGTATACATGCCCAAGTGTGA
GAATGACCGGGTGGAGCTGCCCAGCCGTACCCTCTGCCAGGCCACCCGAGGCCCCTGT
GCCATCGTGGAGAGGGAGCGGGGCTGGCCTGACTTCCTGCGCTGCACTCCTGACCGCT
TCCCTGAAGGCTGCACGAATGAGGTGCAGAACATCAAGTTCAACAGTTCAGGCCAGT
GCGAAGTGCCCTTGGTTCGGACAGACAACCCCAAGAGCTGGTACGAGGACGTGGAGG
GCTGCGGCATCCAGTGCCAGAACCCGCTCTTCACAGAGGCTGAGCACCAGGACATGC
ACAGCTACATCGCGGCCTTCGGGGCCGTCACGGGCCTCTGCACGCTCTTCACCCTGGC
CACATTCGTGGCTGACTGGCGGAACTCGAATCGCTACCCTGCTGTTATTCTCTTCTACG
TCAATGCGTGCTTCTTTGTGGGCAGCATTGGCTGCCTGGCCCAGTTCATGGATGGTGCC
CGCCGAGAGATCGTCTGCCGTGCAGATGGCACCATGAGGCTTGGGGAGCCCACCTCCA
ATGAGACTCTGTCCTGCGTCATCATCTTTGTCATCGTGTACTACGCCCTGATGGCTGGTG
TGGTTTGGTTTGTGGTCCTCACCTATGCCTGGCACACTTCCTTCAAAGCCCTGGGCACC
ACCTACCAGCCTCTCTCGGGCAAGACCTCCTACTTCCACCTGCTCACCTGGTCACTCCC
CTTTGTCCTCACTGTGGCAATCCTTGCTGTGGCGCAGGTGGATGGGGACTCTGTGAGT
GGCATTTGTTTTGTGGGCTACAAGAACTACCGATACCGTGCGGGCTTCGTGCTGGCCCC
AATCGGCCTGGTGCTCATCGTGGGAGGCTACTTCCTCATCCGAGGAGTCATGACTCTGT
TCTCCATCAAGAGCAACCACCCCGGGCTGCTGAGTGAGAAGGCTGCCAGCAAGATCA
ACGAGACCATGCTGCGCCTGGGCATTTTTGGCTTCCTGGCCTTTGGCTTTGTGCTCATT
ACCTTCAGCTGCCACTTCTACGACTTCTTCAACCAGGCTGAGTGGGAGCGCAGCTTCC
GGGACTATGTGCTATGTCAGGCCAATGTGACCATCGGGCTGCCCACCAAGCAGCCCAT
CCCTGACTGTGAGATCAAGAATCGCCCGAGCCTTCTGGTGGAGAAGATCAACCTGTTT

GCCATGTTTGGAACTGGCATCGCCATGAGCACCTGGGTCTGGACCAAGGCCACGCTGC
TCATCTGGAGGCGTACCTGGTGCAGGTTGACTGGGCAGAGTGACGATGAGCCAAAGC
GGATCAAGAAGAGCAAGATGATTGCCAAGGCCTTCTCTAAGCGGCACGAGCTCCTGCA
GAACCCAGGCCAGGAGCTGTCCTTCAGCATGCACACTGTGTCCCACGACGGGCCCGT
GGCGGGCTTGGCCTTTGACCTCAATGAGCCCTCAGCTGATGTCTCCTCTGCCTGGGCC
CAGCATGTCACCAAGATGGTGGCTCGGAGAGGAGCCATACTGCCCCAGGATATTTCTG
TCACCCCTGTGGCAACTCCAGTGCCCCCAGAGGAACAAGCCAACCTGTGGCTGGTTG
AGGCAGAGATCTCCCCAGAGCTGCAGAAGCGCCTGGGCCGGAAGAAGAAGAGGAGG
AAGAGGAAGAAGGAGGTGTGCCCGCTGGCGCCGCCCCCTGAGCTTCACCCCCCTGCC
CCTGCCCCCAGTACCATTCCTCGACTGCCTCAGCTGCCCCGGCAGAAATGCCTGGTGG
CTGCAGGTGCCTGGGGAGCTGGGGACTCTTGCCGACAGGGAGCGTGGACCCTGGTCT
CCAACCCATTCTGCCCAGAGCCCAGTCCCCCTCAGGATCCATTTCTGCCCAGTGCACC
GGCCCCCGTGGCATGGGCTCATGGCCGCCGACAGGGCCTGGGGCCTATTCACTCCCGC
ACCAACCTGATGGACACAGAACTCATGGATGCAGACTCGGACTTCGAACAAAAACTC
ATCTCAGAAGAGGATCTGGACTACAAGGATGACGATGACAAGGATTACAAAGACGAC
GATGATAAGGACTATAAGGATGATGACGACAAATAATCTAGAGGGCCCGTTTAAACCCG
CTGATCAGCCTCGACTGTGCCTTCTAGTTGCCAGCCATCTGTTGTT

Smo L412F:
CTAGAGAACCCACTGCTTACTGGCTTATCGAAATTAATACGACTCACTATAGGGAGACC
CAAGCTGGCTAGCGTTTAAACTTAAGCTTGGTACCGAGCTCGGATCCGCCACCATGGC
CGCTGCCCGCCCAGCGCGGGGGCCGGAGCTCCCGCTCCTGGGGCTGCTGCTGCTGCT
GCTGCTGGGGGACCCGGGCCGGGGGGCGGCCTCGAGCGGGAACGCGACCGGGCCTG
GGCCTCGGAGCGCGGGCGGGAGCGCGAGGAGGAGCGCGGCGGTGACTGGCCCTCCG
CCGCCGCTGAGCCACTGCGGCCGGGCTGCCCCCTGCGAGCCGCTGCGCTACAACGTGT
GCCTGGGCTCGGTGCTGCCCTACGGGGCCACCTCCACACTGCTGGCCGGAGACTCGG
ACTCCCAGGAGGAAGCGCACGGCAAGCTCGTGCTCTGGTCGGGCCTCCGGAATGCCC
CCCGCTGCTGGGCAGTGATCCAGCCCCTGCTGTGTGCCGTATACATGCCCAAGTGTGA
GAATGACCGGGTGGAGCTGCCCAGCCGTACCCTCTGCCAGGCCACCCGAGGCCCCTGT
GCCATCGTGGAGAGGGAGCGGGGCTGGCCTGACTTCCTGCGCTGCACTCCTGACCGCT
TCCCTGAAGGCTGCACGAATGAGGTGCAGAACATCAAGTTCAACAGTTCAGGCCAGT
GCGAAGTGCCCTTGGTTCGGACAGACAACCCCAAGAGCTGGTACGAGGACGTGGAGG
GCTGCGGCATCCAGTGCCAGAACCCGCTCTTCACAGAGGCTGAGCACCAGGACATGC
ACAGCTACATCGCGGCCTTCGGGGCCGTCACGGGCCTCTGCACGCTCTTCACCCTGGC
CACATTCGTGGCTGACTGGCGGAACTCGAATCGCTACCCTGCTGTTATTCTCTTCTACG
TCAATGCGTGCTTCTTTGTGGGCAGCATTGGCTGGCTGGCCCAGTTCATGGATGGTGCC
CGCCGAGAGATCGTCTGCCGTGCAGATGGCACCATGAGGCTTGGGGAGCCCACCTCCA
ATGAGACTCTGTCCTGCGTCATCATCTTTGTCATCGTGTACTACGCCCTGATGGCTGGTG
TGGTTTGGTTTGTGGTCCTCACCTATGCCTGGCACACTTCCTTCAAAGCCCTGGGCACC
ACCTACCAGCCTCTCTCGGGCAAGACCTCCTACTTCCACCTGCTCACCTGGTCACTCCC
CTTTGTCCTCACTGTGGCAATCCTTGCTGTGGCGCAGGTGGATGGGGACTCTGTGAGT
GGCATTTGTTTTGTGGGCTACAAGAACTACCGATACCGTGCGGGCTTCGTGCTGGCCCC
AATCGGCCTGGTGTTCATCGTGGGAGGCTACTTCCTCATCCGAGGAGTCATGACTCTGT
TCTCCATCAAGAGCAACCACCCCGGGCTGCTGAGTGAGAAGGCTGCCAGCAAGATCA

ACGAGACCATGCTGCGCCTGGGCATTTTTGGCTTCCTGGCCTTTGGCTTTGTGCTCATT
ACCTTCAGCTGCCACTTCTACGACTTCTTCAACCAGGCTGAGTGGGAGCGCAGCTTCC
GGGACTATGTGCTATGTCAGGCCAATGTGACCATCGGGCTGCCCACCAAGCAGCCCAT
CCCTGACTGTGAGATCAAGAATCGCCCGAGCCTTCTGGTGGAGAAGATCAACCTGTTT
GCCATGTTTGGAACTGGCATCGCCATGAGCACCTGGGTCTGGACCAAGGCCACGCTGC
TCATCTGGAGGCGTACCTGGTGCAGGTTGACTGGGCAGAGTGACGATGAGCCAAAGC
GGATCAAGAAGAGCAAGATGATTGCCAAGGCCTTCTCTAAGCGGCACGAGCTCCTGCA
GAACCCAGGCCAGGAGCTGTCCTTCAGCATGCACACTGTGTCCCACGACGGGCCCGT
GGCGGGCTTGGCCTTTGACCTCAATGAGCCCTCAGCTGATGTCTCCTCTGCCTGGGCC
CAGCATGTCACCAAGATGGTGGCTCGGAGAGGAGCCATACTGCCCCAGGATATTTCTG
TCACCCCTGTGGCAACTCCAGTGCCCCCAGAGGAACAAGCCAACCTGTGGCTGGTTG
AGGCAGAGATCTCCCCAGAGCTGCAGAAGCGCCTGGGCCGGAAGAAGAAGAGGAGG
AAGAGGAAGAAGGAGGTGTGCCCGCTGGCGCCGCCCCCTGAGCTTCACCCCCCTGCC
CCTGCCCCCAGTACCATTCCTCGACTGCCTCAGCTGCCCCGGCAGAAATGCCTGGTGG
CTGCAGGTGCCTGGGGAGCTGGGGACTCTTGCCGACAGGGAGCGTGGACCCTGGTCT
CCAACCCATTCTGCCCAGAGCCCAGTCCCCCTCAGGATCCATTTCTGCCCAGTGCACC
GGCCCCCGTGGCATGGGCTCATGGCCGCCGACAGGGCCTGGGGCCTATTCACTCCCGC
ACCAACCTGATGGACACAGAACTCATGGATGCAGACTCGGACTTCGAACAAAAACTC
ATCTCAGAAGAGGATCTGGACTACAAGGATGACGATGACAAGGATTACAAAGACGAC
GATGATAAGGACTATAAGGATGATGACGACAAATAATCTAGAGGGCCCGTTTAAACCCG
CTGATCAGCCTCGACTGTGC

Smo F460L:
CTAGAGAACCCACTGCTTACTGGCTTATCGAAATTAATACGACTCACTATAGGGAGACC
CAAGCTGGCTAGCGTTTAAACTTAAGCTTGGTACCGAGCTCGGATCCGCCACCATGGC
CGCTGCCCGCCCAGCGCGGGGGCCGGAGCTCCCGCTCCTGGGGCTGCTGCTGCTGCT
GCTGCTGGGGGACCCGGGCCGGGGGGCGGCCTCGAGCGGGAACGCGACCGGGCCTG
GGCCTCGGAGCGCGGGCGGGAGCGCGAGGAGGAGCGCGGCGGTGACTGGCCCTCCG
CCGCCGCTGAGCCACTGCGGCCGGGCTGCCCCCTGCGAGCCGCTGCGCTACAACGTGT
GCCTGGGCTCGGTGCTGCCCTACGGGGCCACCTCCACACTGCTGGCCGGAGACTCGG
ACTCCCAGGAGGAAGCGCACGGCAAGCTCGTGCTCTGGTCGGGCCTCCGGAATGCCC
CCCGCTGCTGGGCAGTGATCCAGCCCCTGCTGTGTGCCGTATACATGCCCAAGTGTGA
GAATGACCGGGTGGAGCTGCCCAGCCGTACCCTCTGCCAGGCCACCCGAGGCCCCTGT
GCCATCGTGGAGAGGGAGCGGGGCTGGCCTGACTTCCTGCGCTGCACTCCTGACCGCT
TCCCTGAAGGCTGCACGAATGAGGTGCAGAACATCAAGTTCAACAGTTCAGGCCAGT
GCGAAGTGCCCTTGGTTCGGACAGACAACCCCAAGAGCTGGTACGAGGACGTGGAGG
GCTGCGGCATCCAGTGCCAGAACCCGCTCTTCACAGAGGCTGAGCACCAGGACATGC
ACAGCTACATCGCGGCCTTCGGGGCCGTCACGGGCCTCTGCACGCTCTTCACCCTGGC
CACATTCGTGGCTGACTGGCGGAACTCGAATCGCTACCCTGCTGTTATTCTCTTCTACG
TCAATGCGTGCTTCTTTGTGGGCAGCATTGGCTGGCTGGCCCAGTTCATGGATGGTGCC
CGCCGAGAGATCGTCTGCCGTGCAGATGGCACCATGAGGCTTGGGGAGCCCACCTCCA
ATGAGACTCTGTCCTGCGTCATCATCTTTGTCATCGTGTACTACGCCCTGATGGCTGGTG
TGGTTTGGTTTGTGGTCCTCACCTATGCCTGGCACACTTCCTTCAAAGCCCTGGGCACC
ACCTACCAGCCTCTCTCGGGCAAGACCTCCTACTTCCACCTGCTCACCTGGTCACTCCC

CTTTGTCCTCACTGTGGCAATCCTTGCTGTGGCGCAGGTGGATGGGGACTCTGTGAGT
GGCATTTGTTTTGTGGGCTACAAGAACTACCGATACCGTGCGGGCTTCGTGCTGGCCCC
AATCGGCCTGGTGCTCATCGTGGGAGGCTACTTCCTCATCCGAGGAGTCATGACTCTGT
TCTCCATCAAGAGCAACCACCCCGGGCTGCTGAGTGAGAAGGCTGCCAGCAAGATCA
ACGAGACCATGCTGCGCCTGGGCATTTTTGGCTTCCTGGCCTTGGGCTTTGTGCTCATT
ACCTTCAGCTGCCACTTCTACGACTTCTTCAACCAGGCTGAGTGGGAGCGCAGCTTCC
GGGACTATGTGCTATGTCAGGCCAATGTGACCATCGGGCTGCCCACCAAGCAGCCCAT
CCCTGACTGTGAGATCAAGAATCGCCCGAGCCTTCTGGTGGAGAAGATCAACCTGTTT
GCCATGTTTGGAACTGGCATCGCCATGAGCACCTGGGTCTGGACCAAGGCCACGCTGC
TCATCTGGAGGCGTACCTGGTGCAGGTTGACTGGGCAGAGTGACGATGAGCCAAAGC
GGATCAAGAAGAGCAAGATGATTGCCAAGGCCTTCTCTAAGCGGCACGAGCTCCTGCA
GAACCCAGGCCAGGAGCTGTCCTTCAGCATGCACACTGTGTCCCACGACGGGCCCGT
GGCGGGCTTGGCCTTTGACCTCAATGAGCCCTCAGCTGATGTCTCCTCTGCCTGGGCC
CAGCATGTCACCAAGATGGTGGCTCGGAGAGGAGCCATACTGCCCCAGGATATTTCTG
TCACCCCTGTGGCAACTCCAGTGCCCCCAGAGGAACAAGCCAACCTGTGGCTGGTTG
AGGCAGAGATCTCCCCAGAGCTGCAGAAGCGCCTGGGCCGGAAGAAGAAGAGGAGG
AAGAGGAAGAAGGAGGTGTGCCCGCTGGCGCCGCCCCCTGAGCTTCACCCCCCTGCC
CCTGCCCCCAGTACCATTCCTCGACTGCCTCAGCTGCCCCGGCAGAAATGCCTGGTGG
CTGCAGGTGCCTGGGGAGCTGGGGACTCTTGCCGACAGGGAGCGTGGACCCTGGTCT
CCAACCCATTCTGCCCAGAGCCCAGTCCCCCTCAGGATCCATTTCTGCCCAGTGCACC
GGCCCCCGTGGCATGGGCTCATGGCCGCCGACAGGGCCTGGGGCCTATTCACTCCCGC
ACCAACCTGATGGACACAGAACTCATGGATGCAGACTCGGACTTCGAACAAAAACTC
ATCTCAGAAGAGGATCTGGACTACAAGGATGACGATGACAAGGATTACAAAGACGAC
GATGATAAGGACTATAAGGATGATGACGACAAATAATCTAGAGGGCCCGTTTAAACCCG
CTGATCAGCCTCGACTGTGCCTTCTAGTTGCCAGCCATCTGTTGTT

DNA sequencing results are provided below.

Smo D473H:

```
                               1                                                 50
       pSB1296-1     (1) CAGAGCTCTCTGGCTAACTAGAGAACCCACTGCTTACTGGCTTATCGAAA
NM_005631.4 CDS      (1) --------------------------------------------------
                               51                                               100
       pSB1296-1    (51) TTAATACGACTCACTATAGGGAGACCCAAGCTGGCTAGCGTTTAAACTTA
NM_005631.4 CDS      (1) --------------------------------------------------
                               101                                              150
       pSB1296-1   (101) AGCTTGGTACCGAGCTCGGATCCGCCACCATGGCCGCTGCCCGCCCAGCG
NM_005631.4 CDS      (1) -----------------------------ATGGCCGCTGCCCGCCCAGCG
                               151                                              200
       pSB1296-1   (151) CGGGGGCCGGAGCTCCCGCTCCTGGGGCTGCTGCTGCTGCTGCTGCTGGG
NM_005631.4 CDS     (22) CGGGGGCCGGAGCTCCCGCTCCTGGGGCTGCTGCTGCTGCTGCTGCTGGG
                               201                                              250
       pSB1296-1   (201) GGACCCGGGCCGGGGGGCGGCCTCGAGCGGGAACGCGACCGGGCCTGGGC
NM_005631.4 CDS     (72) GGACCCGGGCCGGGGGGCGGCCTCGAGCGGGAACGCGACCGGGCCTGGGC
                               251                                              300
       pSB1296-1   (251) CTCGGAGCGCGGGCGGGAGCGCGAGGAGGAGCGCGGCGGTGACTGGCCCT
NM_005631.4 CDS    (122) CTCGGAGCGCGGGCGGGAGCGCGAGGAGGAGCGCGGCGGTGACTGGCCCT
                               301                                              350
       pSB1296-1   (301) CCGCCGCCGCTGAGCCACTGCGGCCGGGCTGCCCCCTGCGAGCCGCTGCG
NM_005631.4 CDS    (172) CCGCCGCCGCTGAGCCACTGCGGCCGGGCTGCCCCCTGCGAGCCGCTGCG
                               351                                              400
       pSB1296-1   (351) CTACAACGTGTGCCTGGGCTCGGTGCTGCCCTACGGGGCCACCTCCACAC
NM_005631.4 CDS    (222) CTACAACGTGTGCCTGGGCTCGGTGCTGCCCTACGGGGCCACCTCCACAC
                               401                                              450
       pSB1296-1   (401) TGCTGGCCGGAGACTCGGACTCCCAGGAGGAAGCGCACGGCAAGCTCGTG
NM_005631.4 CDS    (272) TGCTGGCCGGAGACTCGGACTCCCAGGAGGAAGCGCACGGCAAGCTCGTG
                               451                                              500
       pSB1296-1   (451) CTCTGGTCGGGCCTCCGGAATGCCCCCCGCTGCTGGGCAGTGATCCAGCC
NM_005631.4 CDS    (322) CTCTGGTCGGGCCTCCGGAATGCCCCCCGCTGCTGGGCAGTGATCCAGCC
                               501                                              550
       pSB1296-1   (501) CCTGCTGTGTGCCGTATACATGCCCAAGTGTGAGAATGACCGGGTGGAGC
NM_005631.4 CDS    (372) CCTGCTGTGTGCCGTATACATGCCCAAGTGTGAGAATGACCGGGTGGAGC
                               551                                              600
       pSB1296-1   (551) TGCCCAGCCGTACCCTCTGCCAGGCCACCCGAGGCCCCTGTGCCATCGTG
NM_005631.4 CDS    (422) TGCCCAGCCGTACCCTCTGCCAGGCCACCCGAGGCCCCTGTGCCATCGTG
                               601                                              650
       pSB1296-1   (601) GAGAGGGAGCGGGGCTGGCCTGACTTCCTGCGCTGCACTCCTGACCGCTT
```

```
NM_005631.4 CDS    (472) GAGAGGGAGCGGGGCTGGCCTGACTTCCTGCGCTGCACTCCTGACCGCTT
                         651                                             700
      pSB1296-1    (651) CCCTGAAGGCTGCACGAATGAGGTGCAGAACATCAAGTTCAACAGTTCAG
NM_005631.4 CDS    (522) CCCTGAAGGCTGCACGAATGAGGTGCAGAACATCAAGTTCAACAGTTCAG
                         701                                             750
      pSB1296-1    (701) GCCAGTGCGAAGTGCCCTTGGTTCGGACAGACAACCCCAAGAGCTGGTAC
NM_005631.4 CDS    (572) GCCAGTGCGAAGTGCCCTTGGTTCGGACAGACAACCCCAAGAGCTGGTAC
                         751                                             800
      pSB1296-1    (751) GAGGACGTGGAGGGCTGCGGCATCCAGTGCCAGAACCCGCTCTTCACAGA
NM_005631.4 CDS    (622) GAGGACGTGGAGGGCTGCGGCATCCAGTGCCAGAACCCGCTCTTCACAGA
                         801                                             850
      pSB1296-1    (801) GGCTGAGCACCAGGACATGCACAGCTACATCGCGGCCTTCGGGGCCGTCA
NM_005631.4 CDS    (672) GGCTGAGCACCAGGACATGCACAGCTACATCGCGGCCTTCGGGGCCGTCA
                         851                                             900
      pSB1296-1    (851) CGGGCCTCTGCACGCTCTTCACCCTGGCCACATTCGTGGCTGACTGGCGG
NM_005631.4 CDS    (722) CGGGCCTCTGCACGCTCTTCACCCTGGCCACATTCGTGGCTGACTGGCGG
                         901                                             950
      pSB1296-1    (901) AACTCGAATCGCTACCCTGCTGTTATTCTCTTCTACGTCAATGCGTGCTT
NM_005631.4 CDS    (772) AACTCGAATCGCTACCCTGCTGTTATTCTCTTCTACGTCAATGCGTGCTT
                         951                                            1000
      pSB1296-1    (951) CTTTGTGGGCAGCATTGGCTGGCTGGCCCAGTTCATGGATGGTGCCCGCC
NM_005631.4 CDS    (822) CTTTGTGGGCAGCATTGGCTGGCTGGCCCAGTTCATGGATGGTGCCCGCC
                         1001                                           1050
      pSB1296-1   (1001) GAGAGATCGTCTGCCGTGCAGATGGCACCATGAGGCTTGGGGAGCCCACC
NM_005631.4 CDS    (872) GAGAGATCGTCTGCCGTGCAGATGGCACCATGAGGCTTGGGGAGCCCACC
                         1051                                           1100
      pSB1296-1   (1051) TCCAATGAGACTCTGTCCTGCGTCATCATCTTTGTCATCGTGTACTACGC
NM_005631.4 CDS    (922) TCCAATGAGACTCTGTCCTGCGTCATCATCTTTGTCATCGTGTACTACGC
                         1101                                           1150
      pSB1296-1   (1101) CCTGATGGCTGGTGTGGTTTGGTTTGTGGTCCTCACCTATGCCTGGCACA
NM_005631.4 CDS    (972) CCTGATGGCTGGTGTGGTTTGGTTTGTGGTCCTCACCTATGCCTGGCACA
                         1151                                           1200
      pSB1296-1   (1151) CTTCCTTCAAAGCCCTGGGCACCACCTACCAGCCTCTCTCGGGCAAGACC
NM_005631.4 CDS   (1022) CTTCCTTCAAAGCCCTGGGCACCACCTACCAGCCTCTCTCGGGCAAGACC
                         1201                                           1250
      pSB1296-1   (1201) TCCTACTTCCACCTGCTCACCTGGTCACTCCCCTTTGTCCTCACTGTGGC
NM_005631.4 CDS   (1072) TCCTACTTCCACCTGCTCACCTGGTCACTCCCCTTTGTCCTCACTGTGGC
                         1251                                           1300
      pSB1296-1   (1251) AATCCTTGCTGTGGCGCAGGTGGATGGGGACTCTGTGAGTGGCATTTGTT
NM_005631.4 CDS   (1122) AATCCTTGCTGTGGCGCAGGTGGATGGGGACTCTGTGAGTGGGATTTGTT
                         1301                                           1350
      pSB1296-1   (1301) TTGTGGGCTACAAGAACTACCGATACCGTGCGGGCTTCGTGCTGGCCCCA
NM_005631.4 CDS   (1172) TTGTGGGCTACAAGAACTACCGATACCGTGCGGGCTTCGTGCTGGCCCCA
                         1351                                           1400
```

```
pSB1296-1       (1351) ATCGGCCTGGTGCTCATCGTGGAGGCTACTTCCTCATCCGAGGAGTCAT
NM_005631.4 CDS (1222) ATCGGCCTGGTGCTCATCGTGGAGGCTACTTCCTCATCCGAGGAGTCAT
                       1401                                           1450
pSB1296-1       (1401) GACTCTGTTCTCCATCAAGAGCAACCACCCCGGGCTGCTGAGTGAGAAGG
NM_005631.4 CDS (1272) GACTCTGTTCTCCATCAAGAGCAACCACCCCGGGCTGCTGAGTGAGAAGG
                       1451                                           1500
pSB1296-1       (1451) CTGCCAGCAAGATCAACGAGACCATGCTGCGCCTGGGCATTTTTGGCTTC
NM_005631.4 CDS (1322) CTGCCAGCAAGATCAACGAGACCATGCTGCGCCTGGGCATTTTTGGCTTC
                       1501                                           1550
pSB1296-1       (1501) CTGGCCTTTGGCTTTGTGCTCATTACCTTCAGCTGCCACTTCTACCACTT
NM_005631.4 CDS (1372) CTGGCCTTTGGCTTTGTGCTCATTACCTTCAGCTGCCACTTCTACGACTT
                       1551                                           1600
pSB1296-1       (1551) CTTCAACCAGGCTGAGTGGGAGCGCAGCTTCCGGGACTATGTGCTATGTC
NM_005631.4 CDS (1422) CTTCAACCAGGCTGAGTGGGAGCGCAGCTTCCGGGACTATGTGCTATGTC
                       1601                                           1650
pSB1296-1       (1601) AGGCCAATGTGACCATCGGGCTGCCCACCAAGCAGCCCATCCCTGACTGT
NM_005631.4 CDS (1472) AGGCCAATGTGACCATCGGGCTGCCCACCAAGCAGCCCATCCCTGACTGT
                       1651                                           1700
pSB1296-1       (1651) GAGATCAAGAATCGCCCGAGCCTTCTGGTGGAGAAGATCAACCTGTTTGC
NM_005631.4 CDS (1522) GAGATCAAGAATCGCCCGAGCCTTCTGGTGGAGAAGATCAACCTGTTTGC
                       1701                                           1750
pSB1296-1       (1701) CATGTTTGGAACTGGCATCGCCATGAGCACCTGGGTCTGGACCAAGGCCA
NM_005631.4 CDS (1572) CATGTTTGGAACTGGCATCGCCATGAGCACCTGGGTCTGGACCAAGGCCA
                       1751                                           1800
pSB1296-1       (1751) CGCTGCTCATCTGGAGGCGTACCTGGTGCAGGTTGACTGGGCAGAGTGAC
NM_005631.4 CDS (1622) CGCTGCTCATCTGGAGGCGTACCTGGTGCAGGTTGACTGGGCAGAGTGAC
                       1801                                           1850
pSB1296-1       (1801) GATGAGCCAAAGCGGATCAAGAAGAGCAAGATGATTGCCAAGGCCTTCTC
NM_005631.4 CDS (1672) GATGAGCCAAAGCGGATCAAGAAGAGCAAGATGATTGCCAAGGCCTTCTC
                       1851                                           1900
pSB1296-1       (1851) TAAGCGGCACGAGCTCCTGCAGAACCCAGGCCAGGAGCTGTCCTTCAGCA
NM_005631.4 CDS (1722) TAAGCGGCACGAGCTCCTGCAGAACCCAGGCCAGGAGCTGTCCTTCAGCA
                       1901                                           1950
pSB1296-1       (1901) TGCACACTGTGTCCCACGACGGGCCCGTGGCGGGCTTGGCCTTTGACCTC
NM_005631.4 CDS (1772) TGCACACTGTGTCCCACGACGGGCCCGTGGCGGGCTTGGCCTTTGACCTC
                       1951                                           2000
pSB1296-1       (1951) AATGAGCCCTCAGCTGATGTCTCCTCTGCCTGGGCCCAGCATGTCACCAA
NM_005631.4 CDS (1822) AATGAGCCCTCAGCTGATGTCTCCTCTGCCTGGGCCCAGCATGTCACCAA
                       2001                                           2050
pSB1296-1       (2001) GATGGTGGCTCGGAGAGGAGCCATACTGCCCCAGGATATTTCTGTCACCC
NM_005631.4 CDS (1872) GATGGTGGCTCGGAGAGGAGCCATACTGCCCCAGGATATTTCTGTCACCC
                       2051                                           2100
pSB1296-1       (2051) CTGTGGCAACTCCAGTGCCCCCAGAGGAACAAGCCAACCTGTGGCTGGTT
NM_005631.4 CDS (1922) CTGTGGCAACTCCAGTGCCCCCAGAGGAACAAGCCAACCTGTGGCTGGTT
```

```
                                    2101                                              2150
        pSB1296-1  (2101) GAGGCAGAGATCTCCCCAGAGCTGCAGAAGCGCCTGGGCCGGAAGAAGAA
NM_005631.4 CDS  (1972) GAGGCAGAGATCTCCCCAGAGCTGCAGAAGCGCCTGGGCCGGAAGAAGAA
                                    2151                                              2200
        pSB1296-1  (2151) GAGGAGGAAGAGGAAGAAGGAGGTGTGCCCGCTGGCGCCGCCCCCTGAGC
NM_005631.4 CDS  (2022) GAGGAGGAAGAGGAAGAAGGAGGTGTGCCCGCTGGCGCCGCCCCCTGAGC
                                    2201                                              2250
        pSB1296-1  (2201) TTCACCCCCCTGCCCCTGCCCCCAGTACCATTCCTCGACTGCCTCAGCTG
NM_005631.4 CDS  (2072) TTCACCCCCCTGCCCCTGCCCCCAGTACCATTCCTCGACTGCCTCAGCTG
                                    2251                                              2300
        pSB1296-1  (2251) CCCCGGCAGAAATGCCTGGTGGCTGCAGGTGCCTGGGGAGCTGGGGACTC
NM_005631.4 CDS  (2122) CCCCGGCAGAAATGCCTGGTGGCTGCAGGTGCCTGGGGAGCTGGGGACTC
                                    2301                                              2350
        pSB1296-1  (2301) TTGCCGACAGGGAGCGTGGACCCTGGTCTCCAACCCATTCTGCCCAGAGC
NM_005631.4 CDS  (2172) TTGCCGACAGGGAGCGTGGACCCTGGTCTCCAACCCATTCTGCCCAGAGC
                                    2351                                              2400
        pSB1296-1  (2351) CCAGTCCCCCTCAGGATCCATTTCTGCCCAGTGCACCGGCCCCCGTGGCA
NM_005631.4 CDS  (2222) CCAGTCCCCCTCAGGATCCATTTCTGCCCAGTGCACCGGCCCCCGTGGCA
                                    2401                                              2450
        pSB1296-1  (2401) TGGGCTCATGGCCGCCGACAGGGCCTGGGGCCTATTCACTCCCGCACCAA
NM_005631.4 CDS  (2272) TGGGCTCATGGCCGCCGACAGGGCCTGGGGCCTATTCACTCCCGCACCAA
                                    2451                                              2500
        pSB1296-1  (2451) CCTGATGGACACAGAACTCATGGATGCAGACTCGGACTTCTGACTCGAGT
NM_005631.4 CDS  (2322) CCTGATGGACACAGAACTCATGGATGCAGACTCGGACTTCTGA-------
                                                                                  2501
                                                      pSB1296-1  (2501) CT
                                              NM_005631.4 CDS  (2365) --
```

Smo W535L

```
                                    1                                                50
        pSB936-4       (1)  TGCGATGGTGCAATAGCAGAGCTCGTTTAGTGACCGTCAGAATTTTGTAA
 NM_005631.4 CDS       (1)  --------------------------------------------------
                                    51                                              100
        pSB936-4      (51)  TACGACTCACTATAGGGCGGCCGGGAATTCGTCGACTGGATCCGGTACCG
 NM_005631.4 CDS       (1)  --------------------------------------------------
                                    101                                             150
        pSB936-4     (101)  AGGAGATCTGCCGCCGCGATCGCCATGGCCGCTGCCCGCCCAGCGCGGGG
 NM_005631.4 CDS       (1)  ------------------------ATGGCCGCTGCCCGCCCAGCGCGGGG
                                    151                                             200
        pSB936-4     (151)  GCCGGAGCTCCCGCTCCTGGGGCTGCTGCTGCTGCTGCTGGGGACC
 NM_005631.4 CDS      (27)  GCCGGAGCTCCCGCTCCTGGGGCTGCTGCTGCTGCTGCTGGGGACC
                                    201                                             250
        pSB936-4     (201)  CGGGCCGGGGGGCGGCCTCGAGCGGGAACGCGACCGGGCCTGGGCCTCGG
 NM_005631.4 CDS      (77)  CGGGCCGGGGGGCGGCCTCGAGCGGGAACGCGACCGGGCCTGGGCCTCGG
                                    251                                             300
        pSB936-4     (251)  AGCGCGGGCGGGAGCGCGAGGAGGAGCGCGGCGGTGACTGGCCCTCCGCC
 NM_005631.4 CDS     (127)  AGCGCGGGCGGGAGCGCGAGGAGGAGCGCGGCGGTGACTGGCCCTCCGCC
                                    301                                             350
        pSB936-4     (301)  GCCGCTGAGCCACTGCGGCCGGGCTGCCCCCTGCGAGCCGCTGCGCTACA
 NM_005631.4 CDS     (177)  GCCGCTGAGCCACTGCGGCCGGGCTGCCCCCTGCGAGCCGCTGCGCTACA
                                    351                                             400
        pSB936-4     (351)  ACGTGTGCCTGGGCTCGGTGCTGCCCTACGGGGCCACCTCCACACTGCTG
 NM_005631.4 CDS     (227)  ACGTGTGCCTGGGCTCGGTGCTGCCCTACGGGGCCACCTCCACACTGCTG
                                    401                                             450
        pSB936-4     (401)  GCCGGAGACTCGGACTCCCAGGAGGAAGCGCACGGCAAGCTCGTGCTCTG
 NM_005631.4 CDS     (277)  GCCGGAGACTCGGACTCCCAGGAGGAAGCGCACGGCAAGCTCGTGCTCTG
                                    451                                             500
        pSB936-4     (451)  GTCGGGCCTCCGGAATGCCCCCCGCTGCTGGGCAGTGATCCAGCCCCTGC
 NM_005631.4 CDS     (327)  GTCGGGCCTCCGGAATGCCCCCCGCTGCTGGGCAGTGATCCAGCCCCTGC
                                    501                                             550
        pSB936-4     (501)  TGTGTGCCGTATACATGCCCAAGTGTGAGAATGACCGGGTGGAGCTGCCC
 NM_005631.4 CDS     (377)  TGTGTGCCGTATACATGCCCAAGTGTGAGAATGACCGGGTGGAGCTGCCC
                                    551                                             600
        pSB936-4     (551)  AGCCGTACCCTCTGCCAGGCCACCCGAGGCCCCTGTGCCATCGTGGAGAG
 NM_005631.4 CDS     (427)  AGCCGTACCCTCTGCCAGGCCACCCGAGGCCCCTGTGCCATCGTGGAGAG
                                    601                                             650
        pSB936-4     (601)  GGAGCGGGGCTGGCCTGACTTCCTGCGCTGCACTCCTGACCGCTTCCCTG
```

```
NM_005631.4 CDS    (477)  GGAGCGGGGCTGGCCTGACTTCCTGCGCTGCACTCCTGACCGCTTCCCTG
                                    651                                    700
       pSB936-4    (651)  AAGGCTGCACGAATGAGGTGCAGAACATCAAGTTCAACAGTTCAGGCCAG
NM_005631.4 CDS    (527)  AAGGCTGCACGAATGAGGTGCAGAACATCAAGTTCAACAGTTCAGGCCAG
                                    701                                    750
       pSB936-4    (701)  TGCGAAGTGCCCTTGGTTCGGACAGACAACCCCAAGAGCTGGTACGAGGA
NM_005631.4 CDS    (577)  TGCGAAGTGCCCTTGGTTCGGACAGACAACCCCAAGAGCTGGTACGAGGA
                                    751                                    800
       pSB936-4    (751)  CGTGGAGGGCTGCGGCATCCAGTGCCAGAACCCGCTCTTCACAGAGGCTG
NM_005631.4 CDS    (627)  CGTGGAGGGCTGCGGCATCCAGTGCCAGAACCCGCTCTTCACAGAGGCTG
                                    801                                    850
       pSB936-4    (801)  AGCACCAGGACATGCACAGCTACATCGCGGCCTTCGGGGCCGTCACGGGC
NM_005631.4 CDS    (677)  AGCACCAGGACATGCACAGCTACATCGCGGCCTTCGGGGCCGTCACGGGC
                                    851                                    900
       pSB936-4    (851)  CTCTGCACGCTCTTCACCCTGGCCACATTCGTGGCTGACTGGCGGAACTC
NM_005631.4 CDS    (727)  CTCTGCACGCTCTTCACCCTGGCCACATTCGTGGCTGACTGGCGGAACTC
                                    901                                    950
       pSB936-4    (901)  GAATCGCTACCCTGCTGTTATTCTCTTCTACGTCAATGCGTGCTTCTTTG
NM_005631.4 CDS    (777)  GAATCGCTACCCTGCTGTTATTCTCTTCTACGTCAATGCGTGCTTCTTTG
                                    951                                   1000
       pSB936-4    (951)  TGGGCAGCATTGGCTGGCTGGCCCAGTTCATGGATGGTGCCCGCCGAGAG
NM_005631.4 CDS    (827)  TGGGCAGCATTGGCTGGCTGGCCCAGTTCATGGATGGTGCCCGCCGAGAG
                                   1001                                   1050
       pSB936-4   (1001)  ATCGTCTGCCGTGCAGATGGCACCATGAGGCTTGGGGAGCCCACCTCCAA
NM_005631.4 CDS    (877)  ATCGTCTGCCGTGCAGATGGCACCATGAGGCTTGGGGAGCCCACCTCCAA
                                   1051                                   1100
       pSB936-4   (1051)  TGAGACTCTGTCCTGCGTCATCATCTTTGTCATCGTGTACTACGCCCTGA
NM_005631.4 CDS    (927)  TGAGACTCTGTCCTGCGTCATCATCTTTGTCATCGTGTACTACGCCCTGA
                                   1101                                   1150
       pSB936-4   (1101)  TGGCTGGTGTGGTTTGGTTTGTGGTCCTCACCTATGCCTGGCACACTTCC
NM_005631.4 CDS    (977)  TGGCTGGTGTGGTTTGGTTTGTGGTCCTCACCTATGCCTGGCACACTTCC
                                   1151                                   1200
       pSB936-4   (1151)  TTCAAAGCCCTGGGCACCACCTACCAGCCTCTCTCGGGCAAGACCTCCTA
NM_005631.4 CDS   (1027)  TTCAAAGCCCTGGGCACCACCTACCAGCCTCTCTCGGGCAAGACCTCCTA
                                   1201                                   1250
       pSB936-4   (1201)  CTTCCACCTGCTCACCTGGTCACTCCCCTTTGTCCTCACTGTGGCAATCC
NM_005631.4 CDS   (1077)  CTTCCACCTGCTCACCTGGTCACTCCCCTTTGTCCTCACTGTGGCAATCC
                                   1251                                   1300
       pSB936-4   (1251)  TTGCTGTGGCGCAGGTGGATGGGGACTCTGTGAGTGGCATTTGTTTGTG
NM_005631.4 CDS   (1127)  TTGCTGTGGCGCAGGTGGATGGGGACTCTGTGAGTGGGATTTGTTTGTG
                                   1301                                   1350
       pSB936-4   (1301)  GGCTACAAGAACTACCGATACCGTGCGGGCTTCGTGCTGGCCCCAATCGG
NM_005631.4 CDS   (1177)  GGCTACAAGAACTACCGATACCGTGCGGGCTTCGTGCTGGCCCCAATCGG
                                   1351                                   1400
```

```
                pSB936-4   (1351) CCTGGTGCTCATCGTGGGAGGCTACTTCCTCATCCGAGGAGTCATGACTC
       NM_005631.4 CDS   (1227) CCTGGTGCTCATCGTGGGAGGCTACTTCCTCATCCGAGGAGTCATGACTC
                                        1401                                              1450
                pSB936-4   (1401) TGTTCTCCATCAAGAGCAACCACCCCGGGCTGCTGAGTGAGAAGGCTGCC
       NM_005631.4 CDS   (1277) TGTTCTCCATCAAGAGCAACCACCCCGGGCTGCTGAGTGAGAAGGCTGCC
                                        1451                                              1500
                pSB936-4   (1451) AGCAAGATCAACGAGACCATGCTGCGCCTGGGCATTTTTGGCTTCCTGGC
       NM_005631.4 CDS   (1327) AGCAAGATCAACGAGACCATGCTGCGCCTGGGCATTTTTGGCTTCCTGGC
                                        1501                                              1550
                pSB936-4   (1501) CTTTGGCTTTGTGCTCATTACCTTCAGCTGCCACTTCTACGACTTCTTCA
       NM_005631.4 CDS   (1377) CTTTGGCTTTGTGCTCATTACCTTCAGCTGCCACTTCTACGACTTCTTCA
                                        1551                                              1600
                pSB936-4   (1551) ACCAGGCTGAGTGGGAGCGCAGCTTCCGGGACTATGTGCTATGTCAGGCC
       NM_005631.4 CDS   (1427) ACCAGGCTGAGTGGGAGCGCAGCTTCCGGGACTATGTGCTATGTCAGGCC
                                        1601                                              1650
                pSB936-4   (1601) AATGTGACCATCGGGCTGCCCACCAAGCAGCCCATCCCTGACTGTGAGAT
       NM_005631.4 CDS   (1477) AATGTGACCATCGGGCTGCCCACCAAGCAGCCCATCCCTGACTGTGAGAT
                                        1651                                              1700
                pSB936-4   (1651) CAAGAATCGCCCGAGCCTTCTGGTGGAGAAGATCAACCTGTTTGCCATGT
       NM_005631.4 CDS   (1527) CAAGAATCGCCCGAGCCTTCTGGTGGAGAAGATCAACCTGTTTGCCATGT
                                        1701                                              1750
                pSB936-4   (1701) TTGGAACTGGCATCGCCATGAGCACCCTCGTCTGGACCAAGGCCACGCTG
       NM_005631.4 CDS   (1577) TTGGAACTGGCATCGCCATGAGCACCTGGGTCTGGACCAAGGCCACGCTG
                                        1751                                              1800
                pSB936-4   (1751) CTCATCTGGAGGCGTACCTGGTGCAGGTTGACTGGGCAGAGTGACGATGA
       NM_005631.4 CDS   (1627) CTCATCTGGAGGCGTACCTGGTGCAGGTTGACTGGGCAGAGTGACGATGA
                                        1801                                              1850
                pSB936-4   (1801) GCCAAAGCGGATCAAGAAGAGCAAGATGATTGCCAAGGCCTTCTCTAAGC
       NM_005631.4 CDS   (1677) GCCAAAGCGGATCAAGAAGAGCAAGATGATTGCCAAGGCCTTCTCTAAGC
                                        1851                                              1900
                pSB936-4   (1851) GGCACGAGCTCCTGCAGAACCCAGGCCAGGAGCTGTCCTTCAGCATGCAC
       NM_005631.4 CDS   (1727) GGCACGAGCTCCTGCAGAACCCAGGCCAGGAGCTGTCCTTCAGCATGCAC
                                        1901                                              1950
                pSB936-4   (1901) ACTGTGTCCCACGACGGGCCCGTGGCGGGCTTGGCCTTTGACCTCAATGA
       NM_005631.4 CDS   (1777) ACTGTGTCCCACGACGGGCCCGTGGCGGGCTTGGCCTTTGACCTCAATGA
                                        1951                                              2000
                pSB936-4   (1951) GCCCTCAGCTGATGTCTCCTCTGCCTGGGCCCAGCATGTCACCAAGATGG
       NM_005631.4 CDS   (1827) GCCCTCAGCTGATGTCTCCTCTGCCTGGGCCCAGCATGTCACCAAGATGG
                                        2001                                              2050
                pSB936-4   (2001) TGGCTCGGAGAGGAGCCATACTGCCCCAGGATATTTCTGTCACCCCTGTG
       NM_005631.4 CDS   (1877) TGGCTCGGAGAGGAGCCATACTGCCCCAGGATATTTCTGTCACCCCTGTG
                                        2051                                              2100
                pSB936-4   (2051) GCAACTCCAGTGCCCCCAGAGGAACAAGCCAACCTGTGGCTGGTTGAGGC
       NM_005631.4 CDS   (1927) GCAACTCCAGTGCCCCCAGAGGAACAAGCCAACCTGTGGCTGGTTGAGGC
```

```
                             2101                                            2150
        pSB936-4  (2101) AGAGATCTCCCCAGAGCTGCAGAAGCGCCTGGGCCGGAAGAAGAAGAGGA
NM_005631.4 CDS  (1977) AGAGATCTCCCCAGAGCTGCAGAAGCGCCTGGGCCGGAAGAAGAAGAGGA
                             2151                                            2200
        pSB936-4  (2151) GGAAGAGGAAGAAGGAGGTGTGCCCGCTGGCGCCGCCCCCTGAGCTTCAC
NM_005631.4 CDS  (2027) GGAAGAGGAAGAAGGAGGTGTGCCCGCTGGCGCCGCCCCCTGAGCTTCAC
                             2201                                            2250
        pSB936-4  (2201) CCCCCTGCCCCTGCCCCCAGTACCATTCCTCGACTGCCTCAGCTGCCCCG
NM_005631.4 CDS  (2077) CCCCCTGCCCCTGCCCCCAGTACCATTCCTCGACTGCCTCAGCTGCCCCG
                             2251                                            2300
        pSB936-4  (2251) GCAGAAATGCCTGGTGGCTGCAGGTGCCTGGGGAGCTGGGGACTCTTGCC
NM_005631.4 CDS  (2127) GCAGAAATGCCTGGTGGCTGCAGGTGCCTGGGGAGCTGGGGACTCTTGCC
                             2301                                            2350
        pSB936-4  (2301) GACAGGGAGCGTGGACCCTGGTCTCCAACCCATTCTGCCCAGAGCCCAGT
NM_005631.4 CDS  (2177) GACAGGGAGCGTGGACCCTGGTCTCCAACCCATTCTGCCCAGAGCCCAGT
                             2351                                            2400
        pSB936-4  (2351) CCCCCTCAGGATCCATTTCTGCCCAGTGCACCGGCCCCCGTGGCATGGGC
NM_005631.4 CDS  (2227) CCCCCTCAGGATCCATTTCTGCCCAGTGCACCGGCCCCCGTGGCATGGGC
                             2401                                            2450
        pSB936-4  (2401) TCATGGCCGCCGACAGGGCCTGGGGCCTATTCACTCCCGCACCAACCTGA
NM_005631.4 CDS  (2277) TCATGGCCGCCGACAGGGCCTGGGGCCTATTCACTCCCGCACCAACCTGA
                             2451                                            2500
        pSB936-4  (2451) TGGACACAGAACTCATGGATGCAGACTCGGACTTCACGCGTACGCGGCCG
NM_005631.4 CDS  (2327) TGGACACAGAACTCATGGATGCAGACTCGGACTTCTGA------------
                             2501                                            2550
        pSB936-4  (2501) CTCGAGCAGAAACTCATCTCAGAAGAGGATCTGGCAGCAAATGATATCCT
NM_005631.4 CDS  (2365) --------------------------------------------------
                             2551                                            2600
        pSB936-4  (2551) GGATTACAAGGATGACGACGATAAGGTTTAAACGGCCGGCCGCGGTCATA
NM_005631.4 CDS  (2365) --------------------------------------------------
                             2601                                            2650
        pSB936-4  (2601) GCTGTTTCCTGAACAGATCCCGGGTGGCATCCCTGTGACCCCTCCCCAGT
NM_005631.4 CDS  (2365) --------------------------------------------------
                             2651                                            2700
        pSB936-4  (2651) GCCTCTCCTGGCCCTGGAAGTTGCCACTCCAGTGCCCACCAGCCTGTCCT
NM_005631.4 CDS  (2365) --------------------------------------------------
                                                  2701        2716
                             pSB936-4  (2701) AATAAATAGTCAACGT
                     NM_005631.4 CDS  (2365) ----------------
```

Smo H231R:

```
                          829                                              878
PSE2260    (829)  CTAGAGAACCCACTGCTTACTGGCTTATCGAAATTAATACGACTCACTAT
                                     PSE2260 seq splicing     (1)
                  CTAGAGAACCCACTGCTTACTGGCTTATCGAAATTAATACGACTCACTAT
                          879                                              928
PSE2260    (879)  AGGGAGACCCAAGCTGGCTAGCGTTTAAACTTAAGCTTGGTACCGAGCTC
                                     PSE2260 seq splicing    (51)
                  AGGGAGACCCAAGCTGGCTAGCGTTTAAACTTAAGCTTGGTACCGAGCTC
                          929                                              978
PSE2260    (929)  GGATCCGCCACCATGGCCGCTGCCCGCCCAGCGCGGGGGCCGGAGCTCCC
                                     PSE2260 seq splicing   (101)
                  GGATCCGCCACCATGGCCGCTGCCCGCCCAGCGCGGGGGCCGGAGCTCCC
                          979                                             1028
PSE2260    (979)  GCTCCTGGGGCTGCTGCTGCTGCTGCTGCTGGGGGACCCGGGCCGGGGGG
                                     PSE2260 seq splicing   (151)
                  GCTCCTGGGGCTGCTGCTGCTGCTGCTGCTGGGGGACCCGGGCCGGGGGG
                         1029                                             1078
PSE2260   (1029)  CGGCCTCGAGCGGGAACGCGACCGGGCCTGGGCCTCGGAGCGCGGGCGGG
                                     PSE2260 seq splicing   (201)
                  CGGCCTCGAGCGGGAACGCGACCGGGCCTGGGCCTCGGAGCGCGGGCGGG
                         1079                                             1128
PSE2260   (1079)  AGCGCGAGGAGGAGCGCGGCGGTGACTGGCCCTCCGCCGCCGCTGAGCCA
                                     PSE2260 seq splicing   (251)
                  AGCGCGAGGAGGAGCGCGGCGGTGACTGGCCCTCCGCCGCCGCTGAGCCA
                         1129                                             1178
PSE2260   (1129)  CTGCGGCCGGGCTGCCCCCTGCGAGCCGCTGCGCTACAACGTGTGCCTGG
                                     PSE2260 seq splicing   (301)
                  CTGCGGCCGGGCTGCCCCCTGCGAGCCGCTGCGCTACAACGTGTGCCTGG
                         1179                                             1228
PSE2260   (1179)  GCTCGGTGCTGCCCTACGGGGCCACCTCCACACTGCTGGCCGGAGACTCG
                                     PSE2260 seq splicing   (351)
                  GCTCGGTGCTGCCCTACGGGGCCACCTCCACACTGCTGGCCGGAGACTCG
                         1229                                             1278
PSE2260   (1229)  GACTCCCAGGAGGAAGCGCACGGCAAGCTCGTGCTCTGGTCGGGCCTCCG
                                     PSE2260 seq splicing   (401)
                  GACTCCCAGGAGGAAGCGCACGGCAAGCTCGTGCTCTGGTCGGGCCTCCG
                         1279                                             1328
PSE2260   (1279)  GAATGCCCCCCGCTGCTGGGCAGTGATCCAGCCCCTGCTGTGTGCCGTAT
                                     PSE2260 seq splicing   (451)
                  GAATGCCCCCCGCTGCTGGGCAGTGATCCAGCCCCTGCTGTGTGCCGTAT
                         1329                                             1378
PSE2260   (1329)  ACATGCCCAAGTGTGAGAATGACCGGGTGGAGCTGCCCAGCCGTACCCTC
```

PSE2260 seq splicing (501)
ACATGCCCAAGTGTGAGAATGACCGGGTGGAGCTGCCCAGCCGTACCCTC
1379 1428
PSE2260 (1379) TGCCAGGCCACCCGAGGCCCCTGTGCCATCGTGGAGAGGGAGCGGGGCTG
PSE2260 seq splicing (551)
TGCCAGGCCACCCGAGGCCCCTGTGCCATCGTGGAGAGGGAGCGGGGCTG
1429 1478
PSE2260 (1429) GCCTGACTTCCTGCGCTGCACTCCTGACCGCTTCCCTGAAGGCTGCACGA
PSE2260 seq splicing (601)
GCCTGACTTCCTGCGCTGCACTCCTGACCGCTTCCCTGAAGGCTGCACGA
1479 1528
PSE2260 (1479) ATGAGGTGCAGAACATCAAGTTCAACAGTTCAGGCCAGTGCGAAGTGCCC
PSE2260 seq splicing (651)
ATGAGGTGCAGAACATCAAGTTCAACAGTTCAGGCCAGTGCGAAGTGCCC
1529 1578
PSE2260 (1529) TTGGTTCGGACAGACAACCCCAAGAGCTGGTACGAGGACGTGGAGGGCTG
PSE2260 seq splicing (701)
TTGGTTCGGACAGACAACCCCAAGAGCTGGTACGAGGACGTGGAGGGCTG
1579 1628
PSE2260 (1579) CGGCATCCAGTGCCAGAACCCGCTCTTCACAGAGGCTGAGCACCAGGACA
PSE2260 seq splicing (751)
CGGCATCCAGTGCCAGAACCCGCTCTTCACAGAGGCTGAGCACCAGGACA
1629 1678
PSE2260 (1629) TGCGCAGCTACATCGCGGCCTTCGGGGCCGTCACGGGCCTCTGCACGCTC
PSE2260 seq splicing (801)
TGCGCAGCTACATCGCGGCCTTCGGGGCCGTCACGGGCCTCTGCACGCTC
1679 1728
PSE2260 (1679) TTCACCCTGGCCACATTCGTGGCTGACTGGCGGAACTCGAATCGCTACCC
PSE2260 seq splicing (851)
TTCACCCTGGCCACATTCGTGGCTGACTGGCGGAACTCGAATCGCTACCC
1729 1778
PSE2260 (1729) TGCTGTTATTCTCTTCTACGTCAATGCGTGCTTCTTTGTGGGCAGCATTG
PSE2260 seq splicing (901)
TGCTGTTATTCTCTTCTACGTCAATGCGTGCTTCTTTGTGGGCAGCATTG
1779 1828
PSE2260 (1779) GCTGGCTGGCCCAGTTCATGGATGGTGCCCGCCGAGAGATCGTCTGCCGT
PSE2260 seq splicing (951)
GCTGGCTGGCCCAGTTCATGGATGGTGCCCGCCGAGAGATCGTCTGCCGT
1829 1878
PSE2260 (1829) GCAGATGGCACCATGAGGCTTGGGGAGCCCACCTCCAATGAGACTCTGTC
PSE2260 seq splicing (1001)
GCAGATGGCACCATGAGGCTTGGGGAGCCCACCTCCAATGAGACTCTGTC
1879 1928
PSE2260 (1879) CTGCGTCATCATCTTTGTCATCGTGTACTACGCCCTGATGGCTGGTGTGG

```
                         PSE2260 seq splicing  (1051)
                  CTGCGTCATCATCTTTGTCATCGTGTACTACGCCCTGATGGCTGGTGTGG
                        1929                                              1978
PSE2260  (1929)  TTTGGTTTGTGGTCCTCACCTATGCCTGGCACACTTCCTTCAAAGCCCTG
                         PSE2260 seq splicing  (1101)
                  TTTGGTTTGTGGTCCTCACCTATGCCTGGCACACTTCCTTCAAAGCCCTG
                        1979                                              2028
PSE2260  (1979)  GGCACCACCTACCAGCCTCTCTCGGGCAAGACCTCCTACTTCCACCTGCT
                         PSE2260 seq splicing  (1151)
                  GGCACCACCTACCAGCCTCTCTCGGGCAAGACCTCCTACTTCCACCTGCT
                        2029                                              2078
PSE2260  (2029)  CACCTGGTCACTCCCCTTTGTCCTCACTGTGGCAATCCTTGCTGTGGCGC
                         PSE2260 seq splicing  (1201)
                  CACCTGGTCACTCCCCTTTGTCCTCACTGTGGCAATCCTTGCTGTGGCGC
                        2079                                              2128
PSE2260  (2079)  AGGTGGATGGGGACTCTGTGAGTGGGATTTGTTTTGTGGGCTACAAGAAC
                         PSE2260 seq splicing  (1251)
                  AGGTGGATGGGGACTCTGTGAGTGGCATTTGTTTTGTGGGCTACAAGAAC
                        2129                                              2178
PSE2260  (2129)  TACCGATACCGTGCGGGCTTCGTGCTGGCCCCAATCGGCCTGGTGCTCAT
                         PSE2260 seq splicing  (1301)
                  TACCGATACCGTGCGGGCTTCGTGCTGGCCCCAATCGGCCTGGTGCTCAT
                        2179                                              2228
PSE2260  (2179)  CGTGGGAGGCTACTTCCTCATCCGAGGAGTCATGACTCTGTTCTCCATCA
                         PSE2260 seq splicing  (1351)
                  CGTGGGAGGCTACTTCCTCATCCGAGGAGTCATGACTCTGTTCTCCATCA
                        2229                                              2278
PSE2260  (2229)  AGAGCAACCACCCCGGGCTGCTGAGTGAGAAGGCTGCCAGCAAGATCAAC
                         PSE2260 seq splicing  (1401)
                  AGAGCAACCACCCCGGGCTGCTGAGTGAGAAGGCTGCCAGCAAGATCAAC
                        2279                                              2328
PSE2260  (2279)  GAGACCATGCTGCGCCTGGGCATTTTTGGCTTCCTGGCCTTTGGCTTTGT
                         PSE2260 seq splicing  (1451)
                  GAGACCATGCTGCGCCTGGGCATTTTTGGCTTCCTGGCCTTTGGCTTTGT
                        2329                                              2378
PSE2260  (2329)  GCTCATTACCTTCAGCTGCCACTTCTACGACTTCTTCAACCAGGCTGAGT
                         PSE2260 seq splicing  (1501)
                  GCTCATTACCTTCAGCTGCCACTTCTACGACTTCTTCAACCAGGCTGAGT
                        2379                                              2428
PSE2260  (2379)  GGGAGCGCAGCTTCCGGGACTATGTGCTATGTCAGGCCAATGTGACCATC
                         PSE2260 seq splicing  (1551)
                  GGGAGCGCAGCTTCCGGGACTATGTGCTATGTCAGGCCAATGTGACCATC
                        2429                                              2478
PSE2260  (2429)  GGGCTGCCCACCAAGCAGCCCATCCCTGACTGTGAGATCAAGAATCGCCC
```

PSE2260 seq splicing (1601)
GGGCTGCCCACCAAGCAGCCCATCCCTGACTGTGAGATCAAGAATCGCCC
2479 2528
PSE2260 (2479) GAGCCTTCTGGTGGAGAAGATCAACCTGTTTGCCATGTTTGGAACTGGCA
PSE2260 seq splicing (1651)
GAGCCTTCTGGTGGAGAAGATCAACCTGTTTGCCATGTTTGGAACTGGCA
2529 2578
PSE2260 (2529) TCGCCATGAGCACCTGGGTCTGGACCAAGGCCACGCTGCTCATCTGGAGG
PSE2260 seq splicing (1701)
TCGCCATGAGCACCTGGGTCTGGACCAAGGCCACGCTGCTCATCTGGAGG
2579 2628
PSE2260 (2579) CGTACCTGGTGCAGGTTGACTGGGCAGAGTGACGATGAGCCAAAGCGGAT
PSE2260 seq splicing (1751)
CGTACCTGGTGCAGGTTGACTGGGCAGAGTGACGATGAGCCAAAGCGGAT
2629 2678
PSE2260 (2629) CAAGAAGAGCAAGATGATTGCCAAGGCCTTCTCTAAGCGGCACGAGCTCC
PSE2260 seq splicing (1801)
CAAGAAGAGCAAGATGATTGCCAAGGCCTTCTCTAAGCGGCACGAGCTCC
2679 2728
PSE2260 (2679) TGCAGAACCCAGGCCAGGAGCTGTCCTTCAGCATGCACACTGTGTCCCAC
PSE2260 seq splicing (1851)
TGCAGAACCCAGGCCAGGAGCTGTCCTTCAGCATGCACACTGTGTCCCAC
2729 2778
PSE2260 (2729) GACGGGCCCGTGGCGGGCTTGGCCTTTGACCTCAATGAGCCCTCAGCTGA
PSE2260 seq splicing (1901)
GACGGGCCCGTGGCGGGCTTGGCCTTTGACCTCAATGAGCCCTCAGCTGA
2779 2828
PSE2260 (2779) TGTCTCCTCTGCCTGGGCCCAGCATGTCACCAAGATGGTGGCTCGGAGAG
PSE2260 seq splicing (1951)
TGTCTCCTCTGCCTGGGCCCAGCATGTCACCAAGATGGTGGCTCGGAGAG
2829 2878
PSE2260 (2829) GAGCCATACTGCCCCAGGATATTTCTGTCACCCCTGTGGCAACTCCAGTG
PSE2260 seq splicing (2001)
GAGCCATACTGCCCCAGGATATTTCTGTCACCCCTGTGGCAACTCCAGTG
2879 2928
PSE2260 (2879) CCCCCAGAGGAACAAGCCAACCTGTGGCTGGTTGAGGCAGAGATCTCCCC
PSE2260 seq splicing (2051)
CCCCCAGAGGAACAAGCCAACCTGTGGCTGGTTGAGGCAGAGATCTCCCC
2929 2978
PSE2260 (2929) AGAGCTGCAGAAGCGCCTGGGCCGGAAGAAGAAGAGGAGGAAGAGGAAGA
PSE2260 seq splicing (2101)
AGAGCTGCAGAAGCGCCTGGGCCGGAAGAAGAAGAGGAGGAAGAGGAAGA
2979 3028
PSE2260 (2979) AGGAGGTGTGCCCGCTGGCGCCGCCCCCTGAGCTTCACCCCCCTGCCCCT

PSE2260 seq splicing (2151)
AGGAGGTGTGCCCGCTGGCGCCGCCCCCTGAGCTTCACCCCCCTGCCCCT

3029 3078
PSE2260 (3029) GCCCCCAGTACCATTCCTCGACTGCCTCAGCTGCCCCGGCAGAAATGCCT
PSE2260 seq splicing (2201)
GCCCCCAGTACCATTCCTCGACTGCCTCAGCTGCCCCGGCAGAAATGCCT

3079 3128
PSE2260 (3079) GGTGGCTGCAGGTGCCTGGGGAGCTGGGGACTCTTGCCGACAGGGAGCGT
PSE2260 seq splicing (2251)
GGTGGCTGCAGGTGCCTGGGGAGCTGGGGACTCTTGCCGACAGGGAGCGT

3129 3178
PSE2260 (3129) GGACCCTGGTCTCCAACCCATTCTGCCCAGAGCCCAGTCCCCCTCAGGAT
PSE2260 seq splicing (2301)
GGACCCTGGTCTCCAACCCATTCTGCCCAGAGCCCAGTCCCCCTCAGGAT

3179 3228
PSE2260 (3179) CCATTTCTGCCCAGTGCACCGGCCCCCGTGGCATGGGCTCATGGCCGCCG
PSE2260 seq splicing (2351)
CCATTTCTGCCCAGTGCACCGGCCCCCGTGGCATGGGCTCATGGCCGCCG

3229 3278
PSE2260 (3229) ACAGGGCCTGGGGCCTATTCACTCCCGCACCAACCTGATGGACACAGAAC
PSE2260 seq splicing (2401)
ACAGGGCCTGGGGCCTATTCACTCCCGCACCAACCTGATGGACACAGAAC

3279 3328
PSE2260 (3279) TCATGGATGCAGACTCGGACTTCGAACAAAAACTCATCTCAGAAGAGGAT
PSE2260 seq splicing (2451)
TCATGGATGCAGACTCGGACTTCGAACAAAAACTCATCTCAGAAGAGGAT

3329 3378
PSE2260 (3329) CTGGACTACAAGGATGACGATGACAAGGATTACAAAGACGACGATGATAA
PSE2260 seq splicing (2501)
CTGGACTACAAGGATGACGATGACAAGGATTACAAAGACGACGATGATAA

3379 3428
PSE2260 (3379) GGACTATAAGGATGATGACGACAAATAATCTAGAGGGCCCGTTTAAACCC
PSE2260 seq splicing (2551)
GGACTATAAGGATGATGACGACAAATAATCTAGAGGGCCCGTTTAAACCC

3429 3475
PSE2260 (3429) GCTGATCAGCCTCGACTGTGCCTTCTAGTTGCCAGCCATCTGTTGTT
PSE2260 seq splicing (2601)
GCTGATCAGCCTCGACTGTGCCTTCTAGTTGCCAGCCATCTGTTGTT

Smo W281C:

```
                       829                                               878
PSE2261    (829)  CTAGAGAACCCACTGCTTACTGGCTTATCGAAATTAATACGACTCACTAT
                                       PSE2261 seq splicing     (1)
                  CTAGAGAACCCACTGCTTACTGGCTTATCGAAATTAATACGACTCACTAT
                       879                                               928
PSE2261    (879)  AGGGAGACCCAAGCTGGCTAGCGTTTAAACTTAAGCTTGGTACCGAGCTC
                                       PSE2261 seq splicing    (51)
                  AGGGAGACCCAAGCTGGCTAGCGTTTAAACTTAAGCTTGGTACCGAGCTC
                       929                                               978
PSE2261    (929)  GGATCCGCCACCATGGCCGCTGCCCGCCCAGCGCGGGGGCCGGAGCTCCC
                                       PSE2261 seq splicing   (101)
                  GGATCCGCCACCATGGCCGCTGCCCGCCCAGCGCGGGGGCCGGAGCTCCC
                       979                                              1028
PSE2261    (979)  GCTCCTGGGGCTGCTGCTGCTGCTGCTGCTGGGGGACCCGGGCCGGGGGG
                                       PSE2261 seq splicing   (151)
                  GCTCCTGGGGCTGCTGCTGCTGCTGCTGCTGGGGGACCCGGGCCGGGGGG
                      1029                                              1078
PSE2261   (1029)  CGGCCTCGAGCGGGAACGCGACCGGGCCTGGGCCTCGGAGCGCGGGCGGG
                                       PSE2261 seq splicing   (201)
                  CGGCCTCGAGCGGGAACGCGACCGGGCCTGGGCCTCGGAGCGCGGGCGGG
                      1079                                              1128
PSE2261   (1079)  AGCGCGAGGAGGAGCGCGGCGGTGACTGGCCCTCCGCCGCCGCTGAGCCA
                                       PSE2261 seq splicing   (251)
                  AGCGCGAGGAGGAGCGCGGCGGTGACTGGCCCTCCGCCGCCGCTGAGCCA
                      1129                                              1178
PSE2261   (1129)  CTGCGGCCGGGCTGCCCCCTGCGAGCCGCTGCGCTACAACGTGTGCCTGG
                                       PSE2261 seq splicing   (301)
                  CTGCGGCCGGGCTGCCCCCTGCGAGCCGCTGCGCTACAACGTGTGCCTGG
                      1179                                              1228
PSE2261   (1179)  GCTCGGTGCTGCCCTACGGGGCCACCTCCACACTGCTGGCCGGAGACTCG
                                       PSE2261 seq splicing   (351)
                  GCTCGGTGCTGCCCTACGGGGCCACCTCCACACTGCTGGCCGGAGACTCG
                      1229                                              1278
PSE2261   (1229)  GACTCCCAGGAGGAAGCGCACGGCAAGCTCGTGCTCTGGTCGGGCCTCCG
                                       PSE2261 seq splicing   (401)
                  GACTCCCAGGAGGAAGCGCACGGCAAGCTCGTGCTCTGGTCGGGCCTCCG
                      1279                                              1328
PSE2261   (1279)  GAATGCCCCCCGCTGCTGGGCAGTGATCCAGCCCCTGCTGTGTGCCGTAT
                                       PSE2261 seq splicing   (451)
                  GAATGCCCCCCGCTGCTGGGCAGTGATCCAGCCCCTGCTGTGTGCCGTAT
                      1329                                              1378
PSE2261   (1329)  ACATGCCCAAGTGTGAGAATGACCGGGTGGAGCTGCCCAGCCGTACCCTC
```

PSE2261 seq splicing (501)
ACATGCCCAAGTGTGAGAATGACCGGGTGGAGCTGCCCAGCCGTACCCTC

1379 1428
PSE2261 (1379) TGCCAGGCCACCCGAGGCCCCTGTGCCATCGTGGAGAGGGAGCGGGGCTG
PSE2261 seq splicing (551)
TGCCAGGCCACCCGAGGCCCCTGTGCCATCGTGGAGAGGGAGCGGGGCTG

1429 1478
PSE2261 (1429) GCCTGACTTCCTGCGCTGCACTCCTGACCGCTTCCCTGAAGGCTGCACGA
PSE2261 seq splicing (601)
GCCTGACTTCCTGCGCTGCACTCCTGACCGCTTCCCTGAAGGCTGCACGA

1479 1528
PSE2261 (1479) ATGAGGTGCAGAACATCAAGTTCAACAGTTCAGGCCAGTGCGAAGTGCCC
PSE2261 seq splicing (651)
ATGAGGTGCAGAACATCAAGTTCAACAGTTCAGGCCAGTGCGAAGTGCCC

1529 1578
PSE2261 (1529) TTGGTTCGGACAGACAACCCCAAGAGCTGGTACGAGGACGTGGAGGGCTG
PSE2261 seq splicing (701)
TTGGTTCGGACAGACAACCCCAAGAGCTGGTACGAGGACGTGGAGGGCTG

1579 1628
PSE2261 (1579) CGGCATCCAGTGCCAGAACCCGCTCTTCACAGAGGCTGAGCACCAGGACA
PSE2261 seq splicing (751)
CGGCATCCAGTGCCAGAACCCGCTCTTCACAGAGGCTGAGCACCAGGACA

1629 1678
PSE2261 (1629) TGCACAGCTACATCGCGGCCTTCGGGGCCGTCACGGGCCTCTGCACGCTC
PSE2261 seq splicing (801)
TGCACAGCTACATCGCGGCCTTCGGGGCCGTCACGGGCCTCTGCACGCTC

1679 1728
PSE2261 (1679) TTCACCCTGGCCACATTCGTGGCTGACTGGCGGAACTCGAATCGCTACCC
PSE2261 seq splicing (851)
TTCACCCTGGCCACATTCGTGGCTGACTGGCGGAACTCGAATCGCTACCC

1729 1778
PSE2261 (1729) TGCTGTTATTCTCTTCTACGTCAATGCGTGCTTCTTTGTGGGCAGCATTG
PSE2261 seq splicing (901)
TGCTGTTATTCTCTTCTACGTCAATGCGTGCTTCTTTGTGGGCAGCATTG

1779 1828
PSE2261 (1779) GCTGCCTGGCCCAGTTCATGGATGGTGCCCGCCGAGAGATCGTCTGCCGT
PSE2261 seq splicing (951)
GCTGCCTGGCCCAGTTCATGGATGGTGCCCGCCGAGAGATCGTCTGCCGT

1829 1878
PSE2261 (1829) GCAGATGGCACCATGAGGCTTGGGGAGCCCACCTCCAATGAGACTCTGTC
PSE2261 seq splicing (1001)
GCAGATGGCACCATGAGGCTTGGGGAGCCCACCTCCAATGAGACTCTGTC

1879 1928
PSE2261 (1879) CTGCGTCATCATCTTTGTCATCGTGTACTACGCCCTGATGGCTGGTGTGG

```
                              PSE2261 seq splicing  (1051)
                  CTGCGTCATCATCTTTGTCATCGTGTACTACGCCCTGATGGCTGGTGTGG
                      1929                                          1978
PSE2261   (1929)  TTTGGTTTGTGGTCCTCACCTATGCCTGGCACACTTCCTTCAAAGCCCTG
                              PSE2261 seq splicing  (1101)
                  TTTGGTTTGTGGTCCTCACCTATGCCTGGCACACTTCCTTCAAAGCCCTG
                      1979                                          2028
PSE2261   (1979)  GGCACCACCTACCAGCCTCTCTCGGGCAAGACCTCCTACTTCCACCTGCT
                              PSE2261 seq splicing  (1151)
                  GGCACCACCTACCAGCCTCTCTCGGGCAAGACCTCCTACTTCCACCTGCT
                      2029                                          2078
PSE2261   (2029)  CACCTGGTCACTCCCCTTTGTCCTCACTGTGGCAATCCTTGCTGTGGCGC
                              PSE2261 seq splicing  (1201)
                  CACCTGGTCACTCCCCTTTGTCCTCACTGTGGCAATCCTTGCTGTGGCGC
                      2079                                          2128
PSE2261   (2079)  AGGTGGATGGGGACTCTGTGAGTGGGATTTGTTTTGTGGGCTACAAGAAC
                              PSE2261 seq splicing  (1251)
                  AGGTGGATGGGGACTCTGTGAGTGGCATTTGTTTTGTGGGCTACAAGAAC
                      2129                                          2178
PSE2261   (2129)  TACCGATACCGTGCGGGCTTCGTGCTGGCCCCAATCGGCCTGGTGCTCAT
                              PSE2261 seq splicing  (1301)
                  TACCGATACCGTGCGGGCTTCGTGCTGGCCCCAATCGGCCTGGTGCTCAT
                      2179                                          2228
PSE2261   (2179)  CGTGGGAGGCTACTTCCTCATCCGAGGAGTCATGACTCTGTTCTCCATCA
                              PSE2261 seq splicing  (1351)
                  CGTGGGAGGCTACTTCCTCATCCGAGGAGTCATGACTCTGTTCTCCATCA
                      2229                                          2278
PSE2261   (2229)  AGAGCAACCACCCCGGGCTGCTGAGTGAGAAGGCTGCCAGCAAGATCAAC
                              PSE2261 seq splicing  (1401)
                  AGAGCAACCACCCCGGGCTGCTGAGTGAGAAGGCTGCCAGCAAGATCAAC
                      2279                                          2328
PSE2261   (2279)  GAGACCATGCTGCGCCTGGGCATTTTTGGCTTCCTGGCCTTTGGCTTTGT
                              PSE2261 seq splicing  (1451)
                  GAGACCATGCTGCGCCTGGGCATTTTTGGCTTCCTGGCCTTTGGCTTTGT
                      2329                                          2378
PSE2261   (2329)  GCTCATTACCTTCAGCTGCCACTTCTACGACTTCTTCAACCAGGCTGAGT
                              PSE2261 seq splicing  (1501)
                  GCTCATTACCTTCAGCTGCCACTTCTACGACTTCTTCAACCAGGCTGAGT
                      2379                                          2428
PSE2261   (2379)  GGGAGCGCAGCTTCCGGGACTATGTGCTATGTCAGGCCAATGTGACCATC
                              PSE2261 seq splicing  (1551)
                  GGGAGCGCAGCTTCCGGGACTATGTGCTATGTCAGGCCAATGTGACCATC
                      2429                                          2478
PSE2261   (2429)  GGGCTGCCCACCAAGCAGCCCATCCCTGACTGTGAGATCAAGAATCGCCC
```

PSE2261 seq splicing (1601)
GGGCTGCCCACCAAGCAGCCCATCCCTGACTGTGAGATCAAGAATCGCCC
2479 2528
PSE2261 (2479) GAGCCTTCTGGTGGAGAAGATCAACCTGTTTGCCATGTTTGGAACTGGCA
PSE2261 seq splicing (1651)
GAGCCTTCTGGTGGAGAAGATCAACCTGTTTGCCATGTTTGGAACTGGCA
2529 2578
PSE2261 (2529) TCGCCATGAGCACCTGGGTCTGGACCAAGGCCACGCTGCTCATCTGGAGG
PSE2261 seq splicing (1701)
TCGCCATGAGCACCTGGGTCTGGACCAAGGCCACGCTGCTCATCTGGAGG
2579 2628
PSE2261 (2579) CGTACCTGGTGCAGGTTGACTGGGCAGAGTGACGATGAGCCAAAGCGGAT
PSE2261 seq splicing (1751)
CGTACCTGGTGCAGGTTGACTGGGCAGAGTGACGATGAGCCAAAGCGGAT
2629 2678
PSE2261 (2629) CAAGAAGAGCAAGATGATTGCCAAGGCCTTCTCTAAGCGGCACGAGCTCC
PSE2261 seq splicing (1801)
CAAGAAGAGCAAGATGATTGCCAAGGCCTTCTCTAAGCGGCACGAGCTCC
2679 2728
PSE2261 (2679) TGCAGAACCCAGGCCAGGAGCTGTCCTTCAGCATGCACACTGTGTCCCAC
PSE2261 seq splicing (1851)
TGCAGAACCCAGGCCAGGAGCTGTCCTTCAGCATGCACACTGTGTCCCAC
2729 2778
PSE2261 (2729) GACGGGCCCGTGGCGGGCTTGGCCTTTGACCTCAATGAGCCCTCAGCTGA
PSE2261 seq splicing (1901)
GACGGGCCCGTGGCGGGCTTGGCCTTTGACCTCAATGAGCCCTCAGCTGA
2779 2828
PSE2261 (2779) TGTCTCCTCTGCCTGGGCCCAGCATGTCACCAAGATGGTGGCTCGGAGAG
PSE2261 seq splicing (1951)
TGTCTCCTCTGCCTGGGCCCAGCATGTCACCAAGATGGTGGCTCGGAGAG
2829 2878
PSE2261 (2829) GAGCCATACTGCCCCAGGATATTTCTGTCACCCCTGTGGCAACTCCAGTG
PSE2261 seq splicing (2001)
GAGCCATACTGCCCCAGGATATTTCTGTCACCCCTGTGGCAACTCCAGTG
2879 2928
PSE2261 (2879) CCCCCAGAGGAACAAGCCAACCTGTGGCTGGTTGAGGCAGAGATCTCCCC
PSE2261 seq splicing (2051)
CCCCCAGAGGAACAAGCCAACCTGTGGCTGGTTGAGGCAGAGATCTCCCC
2929 2978
PSE2261 (2929) AGAGCTGCAGAAGCGCCTGGGCCGGAAGAAGAAGAGGAGGAAGAGGAAGA
PSE2261 seq splicing (2101)
AGAGCTGCAGAAGCGCCTGGGCCGGAAGAAGAAGAGGAGGAAGAGGAAGA
2979 3028
PSE2261 (2979) AGGAGGTGTGCCCGCTGGCGCCGCCCCCTGAGCTTCACCCCCCTGCCCCT

PSE2261 seq splicing (2151)
AGGAGGTGTGCCCGCTGGCGCCGCCCCCTGAGCTTCACCCCCCTGCCCCT

3029 3078
PSE2261 (3029) GCCCCCAGTACCATTCCTCGACTGCCTCAGCTGCCCCGGCAGAAATGCCT
PSE2261 seq splicing (2201)
GCCCCCAGTACCATTCCTCGACTGCCTCAGCTGCCCCGGCAGAAATGCCT

3079 3128
PSE2261 (3079) GGTGGCTGCAGGTGCCTGGGGAGCTGGGGACTCTTGCCGACAGGGAGCGT
PSE2261 seq splicing (2251)
GGTGGCTGCAGGTGCCTGGGGAGCTGGGGACTCTTGCCGACAGGGAGCGT

3129 3178
PSE2261 (3129) GGACCCTGGTCTCCAACCCATTCTGCCCAGAGCCCAGTCCCCCTCAGGAT
PSE2261 seq splicing (2301)
GGACCCTGGTCTCCAACCCATTCTGCCCAGAGCCCAGTCCCCCTCAGGAT

3179 3228
PSE2261 (3179) CCATTTCTGCCCAGTGCACCGGCCCCCGTGGCATGGGCTCATGGCCGCCG
PSE2261 seq splicing (2351)
CCATTTCTGCCCAGTGCACCGGCCCCCGTGGCATGGGCTCATGGCCGCCG

3229 3278
PSE2261 (3229) ACAGGGCCTGGGGCCTATTCACTCCCGCACCAACCTGATGGACACAGAAC
PSE2261 seq splicing (2401)
ACAGGGCCTGGGGCCTATTCACTCCCGCACCAACCTGATGGACACAGAAC

3279 3328
PSE2261 (3279) TCATGGATGCAGACTCGGACTTCGAACAAAAACTCATCTCAGAAGAGGAT
PSE2261 seq splicing (2451)
TCATGGATGCAGACTCGGACTTCGAACAAAAACTCATCTCAGAAGAGGAT

3329 3378
PSE2261 (3329) CTGGACTACAAGGATGACGATGACAAGGATTACAAAGACGACGATGATAA
PSE2261 seq splicing (2501)
CTGGACTACAAGGATGACGATGACAAGGATTACAAAGACGACGATGATAA

3379 3428
PSE2261 (3379) GGACTATAAGGATGATGACGACAAATAATCTAGAGGGCCCGTTTAAACCC
PSE2261 seq splicing (2551)
GGACTATAAGGATGATGACGACAAATAATCTAGAGGGCCCGTTTAAACCC

3429 3475
PSE2261 (3429) GCTGATCAGCCTCGACTGTGCCTTCTAGTTGCCAGCCATCTGTTGTT
PSE2261 seq splicing (2601)
GCTGATCAGCCTCGACTGTGCCTTCTAGTTGCCAGCCATCTGTTGTT

Smo L412F:

```
                      829                                          878
PSE2262   (829)  CTAGAGAACCCACTGCTTACTGGCTTATCGAAATTAATACGACTCACTAT
                               PSE2262 seq splicing     (1)
                 CTAGAGAACCCACTGCTTACTGGCTTATCGAAATTAATACGACTCACTAT
                      879                                          928
PSE2262   (879)  AGGGAGACCCAAGCTGGCTAGCGTTTAAACTTAAGCTTGGTACCGAGCTC
                               PSE2262 seq splicing    (51)
                 AGGGAGACCCAAGCTGGCTAGCGTTTAAACTTAAGCTTGGTACCGAGCTC
                      929                                          978
PSE2262   (929)  GGATCCGCCACCATGGCCGCTGCCCGCCCAGCGCGGGGGCCGGAGCTCCC
                               PSE2262 seq splicing   (101)
                 GGATCCGCCACCATGGCCGCTGCCCGCCCAGCGCGGGGGCCGGAGCTCCC
                      979                                         1028
PSE2262   (979)  GCTCCTGGGGCTGCTGCTGCTGCTGCTGCTGGGGGACCCGGGCCGGGGGG
                               PSE2262 seq splicing   (151)
                 GCTCCTGGGGCTGCTGCTGCTGCTGCTGCTGGGGGACCCGGGCCGGGGGG
                     1029                                         1078
PSE2262  (1029)  CGGCCTCGAGCGGGAACGCGACCGGGCCTGGGCCTCGGAGCGCGGGCGGG
                               PSE2262 seq splicing   (201)
                 CGGCCTCGAGCGGGAACGCGACCGGGCCTGGGCCTCGGAGCGCGGGCGGG
                     1079                                         1128
PSE2262  (1079)  AGCGCGAGGAGGAGCGCGGCGGTGACTGGCCCTCCGCCGCCGCTGAGCCA
                               PSE2262 seq splicing   (251)
                 AGCGCGAGGAGGAGCGCGGCGGTGACTGGCCCTCCGCCGCCGCTGAGCCA
                     1129                                         1178
PSE2262  (1129)  CTGCGGCCGGGCTGCCCCCTGCGAGCCGCTGCGCTACAACGTGTGCCTGG
                               PSE2262 seq splicing   (301)
                 CTGCGGCCGGGCTGCCCCCTGCGAGCCGCTGCGCTACAACGTGTGCCTGG
                     1179                                         1228
PSE2262  (1179)  GCTCGGTGCTGCCCTACGGGGCCACCTCCACACTGCTGGCCGGAGACTCG
                               PSE2262 seq splicing   (351)
                 GCTCGGTGCTGCCCTACGGGGCCACCTCCACACTGCTGGCCGGAGACTCG
                     1229                                         1278
PSE2262  (1229)  GACTCCCAGGAGGAAGCGCACGGCAAGCTCGTGCTCTGGTCGGGCCTCCG
                               PSE2262 seq splicing   (401)
                 GACTCCCAGGAGGAAGCGCACGGCAAGCTCGTGCTCTGGTCGGGCCTCCG
                     1279                                         1328
PSE2262  (1279)  GAATGCCCCCCGCTGCTGGGCAGTGATCCAGCCCCTGCTGTGTGCCGTAT
                               PSE2262 seq splicing   (451)
                 GAATGCCCCCCGCTGCTGGGCAGTGATCCAGCCCCTGCTGTGTGCCGTAT
                     1329                                         1378
PSE2262  (1329)  ACATGCCCAAGTGTGAGAATGACCGGGTGGAGCTGCCCAGCCGTACCCTC
```

PSE2262 seq splicing (501)
ACATGCCCAAGTGTGAGAATGACCGGGTGGAGCTGCCCAGCCGTACCCTC
1379 1428
PSE2262 (1379) TGCCAGGCCACCCGAGGCCCCTGTGCCATCGTGGAGAGGGAGCGGGGCTG
PSE2262 seq splicing (551)
TGCCAGGCCACCCGAGGCCCCTGTGCCATCGTGGAGAGGGAGCGGGGCTG
1429 1478
PSE2262 (1429) GCCTGACTTCCTGCGCTGCACTCCTGACCGCTTCCCTGAAGGCTGCACGA
PSE2262 seq splicing (601)
GCCTGACTTCCTGCGCTGCACTCCTGACCGCTTCCCTGAAGGCTGCACGA
1479 1528
PSE2262 (1479) ATGAGGTGCAGAACATCAAGTTCAACAGTTCAGGCCAGTGCGAAGTGCCC
PSE2262 seq splicing (651)
ATGAGGTGCAGAACATCAAGTTCAACAGTTCAGGCCAGTGCGAAGTGCCC
1529 1578
PSE2262 (1529) TTGGTTCGGACAGACAACCCCAAGAGCTGGTACGAGGACGTGGAGGGCTG
PSE2262 seq splicing (701)
TTGGTTCGGACAGACAACCCCAAGAGCTGGTACGAGGACGTGGAGGGCTG
1579 1628
PSE2262 (1579) CGGCATCCAGTGCCAGAACCCGCTCTTCACAGAGGCTGAGCACCAGGACA
PSE2262 seq splicing (751)
CGGCATCCAGTGCCAGAACCCGCTCTTCACAGAGGCTGAGCACCAGGACA
1629 1678
PSE2262 (1629) TGCACAGCTACATCGCGGCCTTCGGGGCCGTCACGGGCCTCTGCACGCTC
PSE2262 seq splicing (801)
TGCACAGCTACATCGCGGCCTTCGGGGCCGTCACGGGCCTCTGCACGCTC
1679 1728
PSE2262 (1679) TTCACCCTGGCCACATTCGTGGCTGACTGGCGGAACTCGAATCGCTACCC
PSE2262 seq splicing (851)
TTCACCCTGGCCACATTCGTGGCTGACTGGCGGAACTCGAATCGCTACCC
1729 1778
PSE2262 (1729) TGCTGTTATTCTCTTCTACGTCAATGCGTGCTTCTTTGTGGGCAGCATTG
PSE2262 seq splicing (901)
TGCTGTTATTCTCTTCTACGTCAATGCGTGCTTCTTTGTGGGCAGCATTG
1779 1828
PSE2262 (1779) GCTGGCTGGCCCAGTTCATGGATGGTGCCCGCCGAGAGATCGTCTGCCGT
PSE2262 seq splicing (951)
GCTGGCTGGCCCAGTTCATGGATGGTGCCCGCCGAGAGATCGTCTGCCGT
1829 1878
PSE2262 (1829) GCAGATGGCACCATGAGGCTTGGGGAGCCCACCTCCAATGAGACTCTGTC
PSE2262 seq splicing (1001)
GCAGATGGCACCATGAGGCTTGGGGAGCCCACCTCCAATGAGACTCTGTC
1879 1928
PSE2262 (1879) CTGCGTCATCATCTTTGTCATCGTGTACTACGCCCTGATGGCTGGTGTGG

PSE2262 seq splicing (1051)
CTGCGTCATCATCTTTGTCATCGTGTACTACGCCCTGATGGCTGGTGTGG

1929 1978
PSE2262 (1929) TTTGGTTTGTGGTCCTCACCTATGCCTGGCACACTTCCTTCAAAGCCCTG
PSE2262 seq splicing (1101)
TTTGGTTTGTGGTCCTCACCTATGCCTGGCACACTTCCTTCAAAGCCCTG

1979 2028
PSE2262 (1979) GGCACCACCTACCAGCCTCTCTCGGGCAAGACCTCCTACTTCCACCTGCT
PSE2262 seq splicing (1151)
GGCACCACCTACCAGCCTCTCTCGGGCAAGACCTCCTACTTCCACCTGCT

2029 2078
PSE2262 (2029) CACCTGGTCACTCCCCTTTGTCCTCACTGTGGCAATCCTTGCTGTGGCGC
PSE2262 seq splicing (1201)
CACCTGGTCACTCCCCTTTGTCCTCACTGTGGCAATCCTTGCTGTGGCGC

2079 2128
PSE2262 (2079) AGGTGGATGGGGACTCTGTGAGTGGGATTTGTTTTGTGGGCTACAAGAAC
PSE2262 seq splicing (1251)
AGGTGGATGGGGACTCTGTGAGTGGCATTTGTTTTGTGGGCTACAAGAAC

2129 2178
PSE2262 (2129) TACCGATACCGTGCGGGCTTCGTGCTGGCCCCAATCGGCCTGGTGTTCAT
PSE2262 seq splicing (1301)
TACCGATACCGTGCGGGCTTCGTGCTGGCCCCAATCGGCCTGGTGTTCAT

2179 2228
PSE2262 (2179) CGTGGGAGGCTACTTCCTCATCCGAGGAGTCATGACTCTGTTCTCCATCA
PSE2262 seq splicing (1351)
CGTGGGAGGCTACTTCCTCATCCGAGGAGTCATGACTCTGTTCTCCATCA

2229 2278
PSE2262 (2229) AGAGCAACCACCCCGGGCTGCTGAGTGAGAAGGCTGCCAGCAAGATCAAC
PSE2262 seq splicing (1401)
AGAGCAACCACCCCGGGCTGCTGAGTGAGAAGGCTGCCAGCAAGATCAAC

2279 2328
PSE2262 (2279) GAGACCATGCTGCGCCTGGGCATTTTTGGCTTCCTGGCCTTTGGCTTTGT
PSE2262 seq splicing (1451)
GAGACCATGCTGCGCCTGGGCATTTTTGGCTTCCTGGCCTTTGGCTTTGT

2329 2378
PSE2262 (2329) GCTCATTACCTTCAGCTGCCACTTCTACGACTTCTTCAACCAGGCTGAGT
PSE2262 seq splicing (1501)
GCTCATTACCTTCAGCTGCCACTTCTACGACTTCTTCAACCAGGCTGAGT

2379 2428
PSE2262 (2379) GGGAGCGCAGCTTCCGGGACTATGTGCTATGTCAGGCCAATGTGACCATC
PSE2262 seq splicing (1551)
GGGAGCGCAGCTTCCGGGACTATGTGCTATGTCAGGCCAATGTGACCATC

2429 2478
PSE2262 (2429) GGGCTGCCCACCAAGCAGCCCATCCCTGACTGTGAGATCAAGAATCGCCC

PSE2262 seq splicing (1601)
GGGCTGCCCACCAAGCAGCCCATCCCTGACTGTGAGATCAAGAATCGCCC
2479 2528
PSE2262 (2479) GAGCCTTCTGGTGGAGAAGATCAACCTGTTTGCCATGTTTGGAACTGGCA
PSE2262 seq splicing (1651)
GAGCCTTCTGGTGGAGAAGATCAACCTGTTTGCCATGTTTGGAACTGGCA
2529 2578
PSE2262 (2529) TCGCCATGAGCACCTGGGTCTGGACCAAGGCCACGCTGCTCATCTGGAGG
PSE2262 seq splicing (1701)
TCGCCATGAGCACCTGGGTCTGGACCAAGGCCACGCTGCTCATCTGGAGG
2579 2628
PSE2262 (2579) CGTACCTGGTGCAGGTTGACTGGGCAGAGTGACGATGAGCCAAAGCGGAT
PSE2262 seq splicing (1751)
CGTACCTGGTGCAGGTTGACTGGGCAGAGTGACGATGAGCCAAAGCGGAT
2629 2678
PSE2262 (2629) CAAGAAGAGCAAGATGATTGCCAAGGCCTTCTCTAAGCGGCACGAGCTCC
PSE2262 seq splicing (1801)
CAAGAAGAGCAAGATGATTGCCAAGGCCTTCTCTAAGCGGCACGAGCTCC
2679 2728
PSE2262 (2679) TGCAGAACCCAGGCCAGGAGCTGTCCTTCAGCATGCACACTGTGTCCCAC
PSE2262 seq splicing (1851)
TGCAGAACCCAGGCCAGGAGCTGTCCTTCAGCATGCACACTGTGTCCCAC
2729 2778
PSE2262 (2729) GACGGGCCCGTGGCGGGCTTGGCCTTTGACCTCAATGAGCCCTCAGCTGA
PSE2262 seq splicing (1901)
GACGGGCCCGTGGCGGGCTTGGCCTTTGACCTCAATGAGCCCTCAGCTGA
2779 2828
PSE2262 (2779) TGTCTCCTCTGCCTGGGCCCAGCATGTCACCAAGATGGTGGCTCGGAGAG
PSE2262 seq splicing (1951)
TGTCTCCTCTGCCTGGGCCCAGCATGTCACCAAGATGGTGGCTCGGAGAG
2829 2878
PSE2262 (2829) GAGCCATACTGCCCCAGGATATTTCTGTCACCCCTGTGGCAACTCCAGTG
PSE2262 seq splicing (2001)
GAGCCATACTGCCCCAGGATATTTCTGTCACCCCTGTGGCAACTCCAGTG
2879 2928
PSE2262 (2879) CCCCCAGAGGAACAAGCCAACCTGTGGCTGGTTGAGGCAGAGATCTCCCC
PSE2262 seq splicing (2051)
CCCCCAGAGGAACAAGCCAACCTGTGGCTGGTTGAGGCAGAGATCTCCCC
2929 2978
PSE2262 (2929) AGAGCTGCAGAAGCGCCTGGGCCGGAAGAAGAAGAGGAGGAAGAGGAAGA
PSE2262 seq splicing (2101)
AGAGCTGCAGAAGCGCCTGGGCCGGAAGAAGAAGAGGAGGAAGAGGAAGA
2979 3028
PSE2262 (2979) AGGAGGTGTGCCCGCTGGCGCCGCCCCCTGAGCTTCACCCCCCTGCCCCT

PSE2262 seq splicing (2151) AGGAGGTGTGCCCGCTGGCGCCGCCCCCTGAGCTTCACCCCCCTGCCCCT

3029 3078

PSE2262 (3029) GCCCCCAGTACCATTCCTCGACTGCCTCAGCTGCCCCGGCAGAAATGCCT

PSE2262 seq splicing (2201) GCCCCCAGTACCATTCCTCGACTGCCTCAGCTGCCCCGGCAGAAATGCCT

3079 3128

PSE2262 (3079) GGTGGCTGCAGGTGCCTGGGGAGCTGGGGACTCTTGCCGACAGGGAGCGT

PSE2262 seq splicing (2251) GGTGGCTGCAGGTGCCTGGGGAGCTGGGGACTCTTGCCGACAGGGAGCGT

3129 3178

PSE2262 (3129) GGACCCTGGTCTCCAACCCATTCTGCCCAGAGCCCAGTCCCCCTCAGGAT

PSE2262 seq splicing (2301) GGACCCTGGTCTCCAACCCATTCTGCCCAGAGCCCAGTCCCCCTCAGGAT

3179 3228

PSE2262 (3179) CCATTTCTGCCCAGTGCACCGGCCCCCGTGGCATGGGCTCATGGCCGCCG

PSE2262 seq splicing (2351) CCATTTCTGCCCAGTGCACCGGCCCCCGTGGCATGGGCTCATGGCCGCCG

3229 3278

PSE2262 (3229) ACAGGGCCTGGGGCCTATTCACTCCCGCACCAACCTGATGGACACAGAAC

PSE2262 seq splicing (2401) ACAGGGCCTGGGGCCTATTCACTCCCGCACCAACCTGATGGACACAGAAC

3279 3328

PSE2262 (3279) TCATGGATGCAGACTCGGACTTCGAACAAAAACTCATCTCAGAAGAGGAT

PSE2262 seq splicing (2451) TCATGGATGCAGACTCGGACTTCGAACAAAAACTCATCTCAGAAGAGGAT

3329 3378

PSE2262 (3329) CTGGACTACAAGGATGACGATGACAAGGATTACAAAGACGACGATGATAA

PSE2262 seq splicing (2501) CTGGACTACAAGGATGACGATGACAAGGATTACAAAGACGACGATGATAA

3379 3428

PSE2262 (3379) GGACTATAAGGATGATGACGACAAATAATCTAGAGGGCCCGTTTAAACCC

PSE2262 seq splicing (2551) GGACTATAAGGATGATGACGACAAATAATCTAGAGGGCCCGTTTAAACCC

3429 3449

PSE2262 (3429) GCTGATCAGCCTCGACTGTGC

PSE2262 seq splicing (2601) GCTGATCAGCCTCGACTGTGC

Smo F460L:

```
                          829                                              878
PSE2263   (829)  CTAGAGAACCCACTGCTTACTGGCTTATCGAAATTAATACGACTCACTAT
                            PSE2263 seq splicing     (1)
                 CTAGAGAACCCACTGCTTACTGGCTTATCGAAATTAATACGACTCACTAT
                          879                                              928
PSE2263   (879)  AGGGAGACCCAAGCTGGCTAGCGTTTAAACTTAAGCTTGGTACCGAGCTC
                            PSE2263 seq splicing    (51)
                 AGGGAGACCCAAGCTGGCTAGCGTTTAAACTTAAGCTTGGTACCGAGCTC
                          929                                              978
PSE2263   (929)  GGATCCGCCACCATGGCCGCTGCCCGCCCAGCGCGGGGGCCGGAGCTCCC
                            PSE2263 seq splicing   (101)
                 GGATCCGCCACCATGGCCGCTGCCCGCCCAGCGCGGGGGCCGGAGCTCCC
                          979                                             1028
PSE2263   (979)  GCTCCTGGGGCTGCTGCTGCTGCTGCTGCTGGGGGACCCGGGCCGGGGGG
                            PSE2263 seq splicing   (151)
                 GCTCCTGGGGCTGCTGCTGCTGCTGCTGCTGGGGGACCCGGGCCGGGGGG
                          1029                                            1078
PSE2263  (1029)  CGGCCTCGAGCGGGAACGCGACCGGGCCTGGGCCTCGGAGCGCGGGCGGG
                            PSE2263 seq splicing   (201)
                 CGGCCTCGAGCGGGAACGCGACCGGGCCTGGGCCTCGGAGCGCGGGCGGG
                          1079                                            1128
PSE2263  (1079)  AGCGCGAGGAGGAGCGCGGCGGTGACTGGCCCTCCGCCGCCGCTGAGCCA
                            PSE2263 seq splicing   (251)
                 AGCGCGAGGAGGAGCGCGGCGGTGACTGGCCCTCCGCCGCCGCTGAGCCA
                          1129                                            1178
PSE2263  (1129)  CTGCGGCCGGGCTGCCCCCTGCGAGCCGCTGCGCTACAACGTGTGCCTGG
                            PSE2263 seq splicing   (301)
                 CTGCGGCCGGGCTGCCCCCTGCGAGCCGCTGCGCTACAACGTGTGCCTGG
                          1179                                            1228
PSE2263  (1179)  GCTCGGTGCTGCCCTACGGGGCCACCTCCACACTGCTGGCCGGAGACTCG
                            PSE2263 seq splicing   (351)
                 GCTCGGTGCTGCCCTACGGGGCCACCTCCACACTGCTGGCCGGAGACTCG
                          1229                                            1278
PSE2263  (1229)  GACTCCCAGGAGGAAGCGCACGGCAAGCTCGTGCTCTGGTCGGGCCTCCG
                            PSE2263 seq splicing   (401)
                 GACTCCCAGGAGGAAGCGCACGGCAAGCTCGTGCTCTGGTCGGGCCTCCG
                          1279                                            1328
PSE2263  (1279)  GAATGCCCCCCGCTGCTGGGCAGTGATCCAGCCCCTGCTGTGTGCCGTAT
                            PSE2263 seq splicing   (451)
                 GAATGCCCCCCGCTGCTGGGCAGTGATCCAGCCCCTGCTGTGTGCCGTAT
                          1329                                            1378
PSE2263  (1329)  ACATGCCCAAGTGTGAGAATGACCGGGTGGAGCTGCCCAGCCGTACCCTC
```

PSE2263 seq splicing (501)
ACATGCCCAAGTGTGAGAATGACCGGGTGGAGCTGCCCAGCCGTACCCTC
1379 1428
PSE2263 (1379) TGCCAGGCCACCCGAGGCCCCTGTGCCATCGTGGAGAGGGAGCGGGGCTG
PSE2263 seq splicing (551)
TGCCAGGCCACCCGAGGCCCCTGTGCCATCGTGGAGAGGGAGCGGGGCTG
1429 1478
PSE2263 (1429) GCCTGACTTCCTGCGCTGCACTCCTGACCGCTTCCCTGAAGGCTGCACGA
PSE2263 seq splicing (601)
GCCTGACTTCCTGCGCTGCACTCCTGACCGCTTCCCTGAAGGCTGCACGA
1479 1528
PSE2263 (1479) ATGAGGTGCAGAACATCAAGTTCAACAGTTCAGGCCAGTGCGAAGTGCCC
PSE2263 seq splicing (651)
ATGAGGTGCAGAACATCAAGTTCAACAGTTCAGGCCAGTGCGAAGTGCCC
1529 1578
PSE2263 (1529) TTGGTTCGGACAGACAACCCCAAGAGCTGGTACGAGGACGTGGAGGGCTG
PSE2263 seq splicing (701)
TTGGTTCGGACAGACAACCCCAAGAGCTGGTACGAGGACGTGGAGGGCTG
1579 1628
PSE2263 (1579) CGGCATCCAGTGCCAGAACCCGCTCTTCACAGAGGCTGAGCACCAGGACA
PSE2263 seq splicing (751)
CGGCATCCAGTGCCAGAACCCGCTCTTCACAGAGGCTGAGCACCAGGACA
1629 1678
PSE2263 (1629) TGCACAGCTACATCGCGGCCTTCGGGGCCGTCACGGGCCTCTGCACGCTC
PSE2263 seq splicing (801)
TGCACAGCTACATCGCGGCCTTCGGGGCCGTCACGGGCCTCTGCACGCTC
1679 1728
PSE2263 (1679) TTCACCCTGGCCACATTCGTGGCTGACTGGCGGAACTCGAATCGCTACCC
PSE2263 seq splicing (851)
TTCACCCTGGCCACATTCGTGGCTGACTGGCGGAACTCGAATCGCTACCC
1729 1778
PSE2263 (1729) TGCTGTTATTCTCTTCTACGTCAATGCGTGCTTCTTTGTGGGCAGCATTG
PSE2263 seq splicing (901)
TGCTGTTATTCTCTTCTACGTCAATGCGTGCTTCTTTGTGGGCAGCATTG
1779 1828
PSE2263 (1779) GCTGGCTGGCCCAGTTCATGGATGGTGCCCGCCGAGAGATCGTCTGCCGT
PSE2263 seq splicing (951)
GCTGGCTGGCCCAGTTCATGGATGGTGCCCGCCGAGAGATCGTCTGCCGT
1829 1878
PSE2263 (1829) GCAGATGGCACCATGAGGCTTGGGGAGCCCACCTCCAATGAGACTCTGTC
PSE2263 seq splicing (1001)
GCAGATGGCACCATGAGGCTTGGGGAGCCCACCTCCAATGAGACTCTGTC
1879 1928
PSE2263 (1879) CTGCGTCATCATCTTTGTCATCGTGTACTACGCCCTGATGGCTGGTGTGG

PSE2263 seq splicing (1051)
CTGCGTCATCATCTTTGTCATCGTGTACTACGCCCTGATGGCTGGTGTGG

1929 2028 1978

PSE2263 (1929) TTTGGTTTGTGGTCCTCACCTATGCCTGGCACACTTCCTTCAAAGCCCTG
PSE2263 seq splicing (1101) TTTGGTTTGTGGTCCTCACCTATGCCTGGCACACTTCCTTCAAAGCCCTG

1979 2028

PSE2263 (1979) GGCACCACCTACCAGCCTCTCTCGGGCAAGACCTCCTACTTCCACCTGCT
PSE2263 seq splicing (1151) GGCACCACCTACCAGCCTCTCTCGGGCAAGACCTCCTACTTCCACCTGCT

2029 2078

PSE2263 (2029) CACCTGGTCACTCCCCTTTGTCCTCACTGTGGCAATCCTTGCTGTGGCGC
PSE2263 seq splicing (1201) CACCTGGTCACTCCCCTTTGTCCTCACTGTGGCAATCCTTGCTGTGGCGC

2079 2128

PSE2263 (2079) AGGTGGATGGGGACTCTGTGAGTGGGATTTGTTTTGTGGGCTACAAGAAC
PSE2263 seq splicing (1251) AGGTGGATGGGGACTCTGTGAGTGGCATTTGTTTTGTGGGCTACAAGAAC

2129 2178

PSE2263 (2129) TACCGATACCGTGCGGGCTTCGTGCTGGCCCCAATCGGCCTGGTGCTCAT
PSE2263 seq splicing (1301) TACCGATACCGTGCGGGCTTCGTGCTGGCCCCAATCGGCCTGGTGCTCAT

2179 2228

PSE2263 (2179) CGTGGGAGGCTACTTCCTCATCCGAGGAGTCATGACTCTGTTCTCCATCA
PSE2263 seq splicing (1351) CGTGGGAGGCTACTTCCTCATCCGAGGAGTCATGACTCTGTTCTCCATCA

2229 2278

PSE2263 (2229) AGAGCAACCACCCCGGGCTGCTGAGTGAGAAGGCTGCCAGCAAGATCAAC
PSE2263 seq splicing (1401) AGAGCAACCACCCCGGGCTGCTGAGTGAGAAGGCTGCCAGCAAGATCAAC

2279 2328

PSE2263 (2279) GAGACCATGCTGCGCCTGGGCATTTTTGGCTTCCTGGCCTTGGGCTTTGT
PSE2263 seq splicing (1451) GAGACCATGCTGCGCCTGGGCATTTTTGGCTTCCTGGCCTTGGGCTTTGT

2329 2378

PSE2263 (2329) GCTCATTACCTTCAGCTGCCACTTCTACGACTTCTTCAACCAGGCTGAGT
PSE2263 seq splicing (1501) GCTCATTACCTTCAGCTGCCACTTCTACGACTTCTTCAACCAGGCTGAGT

2379 2428

PSE2263 (2379) GGGAGCGCAGCTTCCGGGACTATGTGCTATGTCAGGCCAATGTGACCATC
PSE2263 seq splicing (1551) GGGAGCGCAGCTTCCGGGACTATGTGCTATGTCAGGCCAATGTGACCATC

2429 2478

PSE2263 (2429) GGGCTGCCCACCAAGCAGCCCATCCCTGACTGTGAGATCAAGAATCGCCC

PSE2263 seq splicing (1601)
GGGCTGCCCACCAAGCAGCCCATCCCTGACTGTGAGATCAAGAATCGCCC

2479 2528
PSE2263 (2479) GAGCCTTCTGGTGGAGAAGATCAACCTGTTTGCCATGTTTGGAACTGGCA
PSE2263 seq splicing (1651) GAGCCTTCTGGTGGAGAAGATCAACCTGTTTGCCATGTTTGGAACTGGCA

2529 2578
PSE2263 (2529) TCGCCATGAGCACCTGGGTCTGGACCAAGGCCACGCTGCTCATCTGGAGG
PSE2263 seq splicing (1701) TCGCCATGAGCACCTGGGTCTGGACCAAGGCCACGCTGCTCATCTGGAGG

2579 2628
PSE2263 (2579) CGTACCTGGTGCAGGTTGACTGGGCAGAGTGACGATGAGCCAAAGCGGAT
PSE2263 seq splicing (1751) CGTACCTGGTGCAGGTTGACTGGGCAGAGTGACGATGAGCCAAAGCGGAT

2629 2678
PSE2263 (2629) CAAGAAGAGCAAGATGATTGCCAAGGCCTTCTCTAAGCGGCACGAGCTCC
PSE2263 seq splicing (1801) CAAGAAGAGCAAGATGATTGCCAAGGCCTTCTCTAAGCGGCACGAGCTCC

2679 2728
PSE2263 (2679) TGCAGAACCCAGGCCAGGAGCTGTCCTTCAGCATGCACACTGTGTCCCAC
PSE2263 seq splicing (1851) TGCAGAACCCAGGCCAGGAGCTGTCCTTCAGCATGCACACTGTGTCCCAC

2729 2778
PSE2263 (2729) GACGGGCCCGTGGCGGGCTTGGCCTTTGACCTCAATGAGCCCTCAGCTGA
PSE2263 seq splicing (1901) GACGGGCCCGTGGCGGGCTTGGCCTTTGACCTCAATGAGCCCTCAGCTGA

2779 2828
PSE2263 (2779) TGTCTCCTCTGCCTGGGCCCAGCATGTCACCAAGATGGTGGCTCGGAGAG
PSE2263 seq splicing (1951) TGTCTCCTCTGCCTGGGCCCAGCATGTCACCAAGATGGTGGCTCGGAGAG

2829 2878
PSE2263 (2829) GAGCCATACTGCCCCAGGATATTTCTGTCACCCCTGTGGCAACTCCAGTG
PSE2263 seq splicing (2001) GAGCCATACTGCCCCAGGATATTTCTGTCACCCCTGTGGCAACTCCAGTG

2879 2928
PSE2263 (2879) CCCCCAGAGGAACAAGCCAACCTGTGGCTGGTTGAGGCAGAGATCTCCCC
PSE2263 seq splicing (2051) CCCCCAGAGGAACAAGCCAACCTGTGGCTGGTTGAGGCAGAGATCTCCCC

2929 2978
PSE2263 (2929) AGAGCTGCAGAAGCGCCTGGGCCGGAAGAAGAAGAGGAGGAAGAGGAAGA
PSE2263 seq splicing (2101) AGAGCTGCAGAAGCGCCTGGGCCGGAAGAAGAAGAGGAGGAAGAGGAAGA

2979 3028
PSE2263 (2979) AGGAGGTGTGCCCGCTGGCGCCGCCCCCTGAGCTTCACCCCCCTGCCCCT

PSE2263 seq splicing (2151)

AGGAGGTGTGCCCGCTGGCGCCGCCCCCTGAGCTTCACCCCCCTGCCCCT

3029 3078

PSE2263 (3029) GCCCCCAGTACCATTCCTCGACTGCCTCAGCTGCCCCGGCAGAAATGCCT

PSE2263 seq splicing (2201)

GCCCCCAGTACCATTCCTCGACTGCCTCAGCTGCCCCGGCAGAAATGCCT

3079 3128

PSE2263 (3079) GGTGGCTGCAGGTGCCTGGGGAGCTGGGGACTCTTGCCGACAGGGAGCGT

PSE2263 seq splicing (2251)

GGTGGCTGCAGGTGCCTGGGGAGCTGGGGACTCTTGCCGACAGGGAGCGT

3129 3178

PSE2263 (3129) GGACCCTGGTCTCCAACCCATTCTGCCCAGAGCCCAGTCCCCCTCAGGAT

PSE2263 seq splicing (2301)

GGACCCTGGTCTCCAACCCATTCTGCCCAGAGCCCAGTCCCCCTCAGGAT

3179 3228

PSE2263 (3179) CCATTTCTGCCCAGTGCACCGGCCCCCGTGGCATGGGCTCATGGCCGCCG

PSE2263 seq splicing (2351)

CCATTTCTGCCCAGTGCACCGGCCCCCGTGGCATGGGCTCATGGCCGCCG

3229 3278

PSE2263 (3229) ACAGGGCCTGGGGCCTATTCACTCCCGCACCAACCTGATGGACACAGAAC

PSE2263 seq splicing (2401)

ACAGGGCCTGGGGCCTATTCACTCCCGCACCAACCTGATGGACACAGAAC

3279 3328

PSE2263 (3279) TCATGGATGCAGACTCGGACTTCGAACAAAAACTCATCTCAGAAGAGGAT

PSE2263 seq splicing (2451)

TCATGGATGCAGACTCGGACTTCGAACAAAAACTCATCTCAGAAGAGGAT

3329 3378

PSE2263 (3329) CTGGACTACAAGGATGACGATGACAAGGATTACAAAGACGACGATGATAA

PSE2263 seq splicing (2501)

CTGGACTACAAGGATGACGATGACAAGGATTACAAAGACGACGATGATAA

3379 3428

PSE2263 (3379) GGACTATAAGGATGATGACGACAAATAATCTAGAGGGCCCGTTTAAACCC

PSE2263 seq splicing (2551)

GGACTATAAGGATGATGACGACAAATAATCTAGAGGGCCCGTTTAAACCC

3429 3475

PSE2263 (3429) GCTGATCAGCCTCGACTGTGCCTTCTAGTTGCCAGCCATCTGTTGTT

PSE2263 seq splicing (2601)

GCTGATCAGCCTCGACTGTGCCTTCTAGTTGCCAGCCATCTGTTGTT
